# Cause of Death During Renal Cell Carcinoma Survivorship: A Contemporary, Population-Based Analysis

**DOI:** 10.3389/fonc.2022.864132

**Published:** 2022-06-02

**Authors:** Dong-Dong Yu, Wei-Kang Chen, Chen-Yu Wu, Wan-Ting Wu, Xiao Xin, Yu-Li Jiang, Peng Li, Ming-Hua Zhang

**Affiliations:** ^1^Department of Urology, Huzhou Central Hospital, Affiliated Central Hospital Huzhou University, Huzhou, China; ^2^Department of Reproductive Endocrinology, Women’s Hospital, School of Medicine, Zhejiang University, Hangzhou, China; ^3^Department of Orthopaedic Surgery, Second Affiliated Hospital of Wenzhou Medical University, Wenzhou, China; ^4^Department of Clinical Medicine, Huzhou University, Huzhou, China

**Keywords:** cause of death, renal cell cancer, standardized mortality ratios (SMRs), noncancer cause of death, Surveillance, Epidemiology, and End Results (SEER)

## Abstract

**Background:**

As the survival rates of patients with renal cell carcinoma (RCC) continue to increase, noncancer causes of death cannot be ignored. The cause-specific mortality in patients with RCC is not well understood.

**Objective:**

Our study aimed to explore the mortality patterns of contemporary RCC survivors.

**Methods:**

We performed a retrospective cohort study involving patients with RCC from the Surveillance, Epidemiology, and End Results (SEER) database. We used standardized mortality ratios (SMRs) to compare the death rates in patients with RCC with those in the general population.

**Results:**

A total of 106,118 patients with RCC, including 39,630 who died (27%), were included in our study. Overall, compared with the general US population, noncancer SMRs were increased 1.25-fold (95% confidence intervals [CI], 1.22 to 1.27; observed, 11,235), 1.19-fold (95% CI, 1.14 to 1.24; observed, 2,014), and 2.24-fold (95% CI, 2.11 to 2.38; observed, 1,110) for stage I/II, III, and IV RCC, respectively. The proportion of noncancer causes of death increased with the extension of survival time. A total of 4,273 men with stage I/II disease (23.13%) died of RCC; however, patients who died from other causes were 3.2 times more likely to die from RCC (*n* = 14,203 [76.87%]). Heart disease was the most common noncancer cause of death (*n* = 3,718 [20.12%]; SMR, 1.23; 95% CI, 1.19–1.27). In patients with stage III disease, 3,912 (25.98%) died from RCC, and 2,014 (13.37%) died from noncancer causes. Most patients (94.99%) with stage IV RCC died within 5 years of initial diagnosis. Although RCC was the leading cause of death (*n* = 12,310 [84.65%]), patients with stage IV RCC also had a higher risk of noncancer death than the general population (2.24; 95% CI, 2.11–2.38).

**Conclusions:**

Non-RCC death causes account for more than 3/4 of RCC survivors among patients with stage I/II disease. Patients with stage IV are most likely to die of RCC; however, there is an increased risk of dying from septicemia, and suicide cannot be ignored. These data provide the latest and most comprehensive assessment of the causes of death in patients with RCC.

## Introduction

Renal cell carcinoma (RCC) is one of the top 10 most prevalent cancers and accounts for 4% of all new malignancies in the United States (U.S.) (1, 2). Approximately 1.7% of people are diagnosed with kidney cancer at some point in their lives (1, 2). As treatment has improved, the death rate from kidney cancer has decreased. It is estimated that there will be 793,530 cancer survivors in the U.S. by 2030 (1–3). Therefore, understanding the real causes of death could help prioritize death risk during survivorship and may provide a roadmap for reducing the mortality burden after RCC.

The causes of death from prostate cancer, colon cancer, testicular cancer, and other cancers have been well described (4–9). Several studies have described the causes of death in patients with RCC. However, these studies were not compared with the risk in the general population, either based on small sample studies, or limited to secondary primary tumors, and the classification of the cause of death is not detailed enough (10–12). To address these limitations, we assessed contemporary, population-based data on the causes of death during RCC survivorship. We present our results based on patient characteristics and the AJCC 6th stage, and the risk of death from each cause was compared with that of the standard population.

## Materials and Methods

### Data Source

This was a retrospective, observational, cohort study. We used data from the National Cancer Institute’s Surveillance, Epidemiology, and End Results (SEER) 18 registries, November 2020 submission (2000 to 2018) for SMRs, which covers approximately 34.6% of the U.S. population.

### Patients

**Figure 1:**
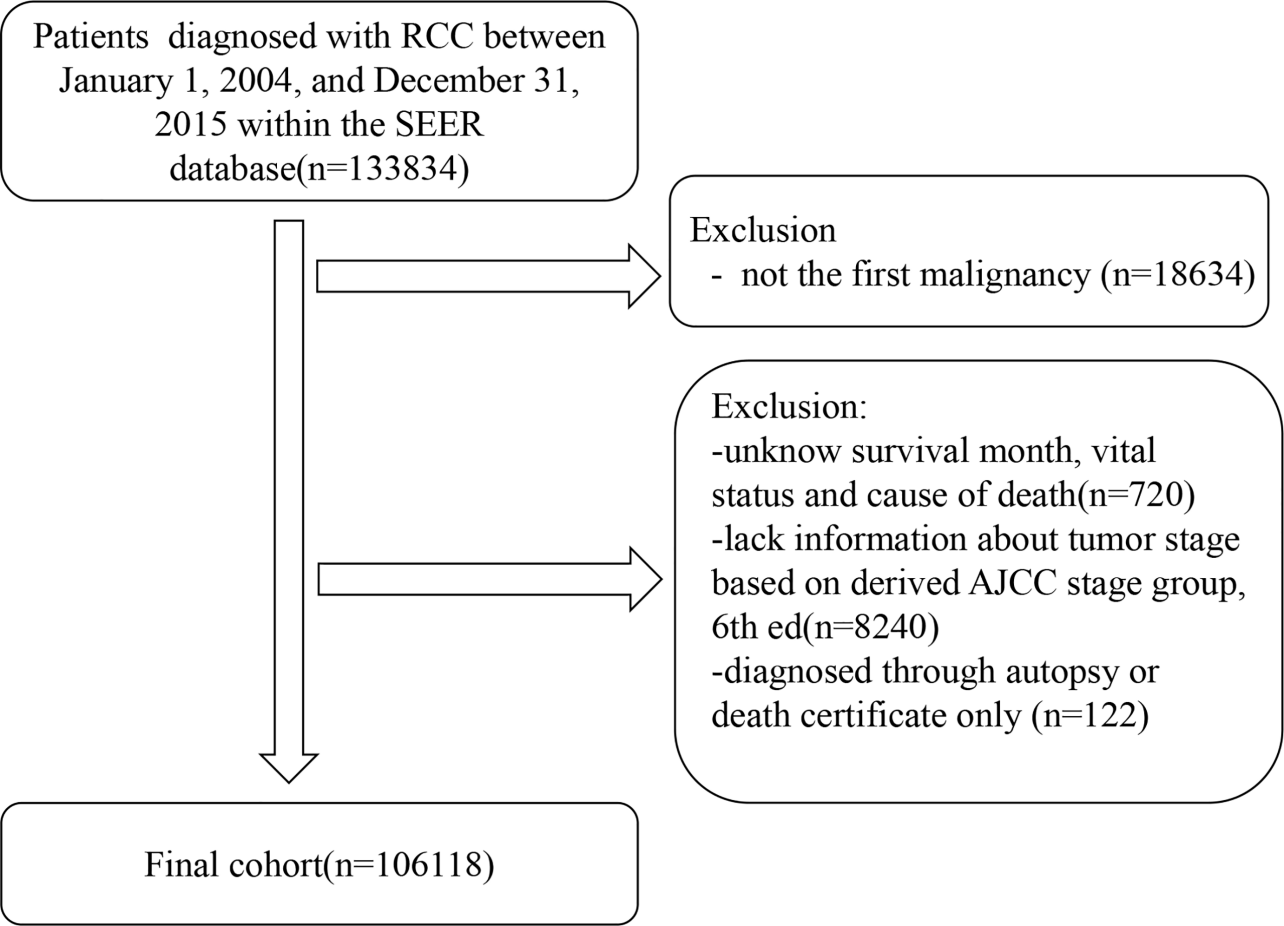
Flowchart of participant selection.

We included all patients diagnosed with RCC as their first malignant tumor between January 1, 2004, and December 31, 2015. Patients with an unknown follow-up time, vital status, and staging information (I, II, III, or IV) were excluded. We excluded patients diagnosed through autopsy or death certificates only. **Figure 1** shows the inclusion and exclusion criteria of the present study.

### Exposures for Stratification

We included the following covariates: stage of disease based on the derived AJCC stage group, 6th ed (I/II vs. III vs. IV;), sex (male or female), age (<65 or ≥65 years old), race (white, black, or other), year of diagnosis (2004–2007, 2008–2011, or 2012–2015), histology type (clear cell, papillary, chromophobe, or other), surgery (yes or no), radiation (yes or no), chemotherapy (yes or no), and marital status (married, never married, or other).

### Outcome Assessments

The outcome variable of interest was overall survival after RCC diagnosis. The SEER cause of death code was based on the International Statistical Classification of Diseases and Related Health Problems 10th, 1999 (ICD‐10).

### Ethics Statement

We were granted permission from the National Cancer Institute USA to access the SEER dataset for research purposes only (reference number: 20025-Nov2020). All data from the SEER database were de-identified, and the extracted data did not require informed consent.

### Statistical Analyses

We used standardized mortality ratios (SMRs), defined as the observed number of deaths divided by the expected number. The expected numbers of deaths were calculated based on age- and sex-specific mortality rates in a standard population. The follow-up time began from the date of first diagnosis to the date of death, loss to follow-up (date of the last visit), or December 2018, whichever came first. The 95% confidence intervals (CIs) for SMRs were estimated using exact methods. All SMRs were generated using the SEER*Stat version 8.3.9.2.

## Results

### Baseline Characteristics

Of the 106,118 patients with RCC, 39,630 (37.35%) died. The median follow-up duration was 68 months (Q1–Q3, 38–109 months). The number of male patients (*n* = 67,020 [63.16%]) was 1.71 times higher than that of female patients (*n* = 39,098 [36.84%]). Most patients (*n* = 74,733 [70.42%]) had stage I/II disease, whereas only 14.19% (*n* = 15,058) and 15.39% (*n* = 16,327) had stage III and IV disease, respectively. The majority of the patients (*n* = 83,132 [78.34%]) had clear cell histology type, and 87,350 (82.31%) were white. Most patients received cancer-directed surgery (*n* = 91,742 [86.45%]). The basic characteristics of the patients with RCC and the number of deaths at different follow-up times are shown in **Table 1**.

**Table 1:** Patient characteristics and death by time after diagnosis.

Stage	Group	All patients Diagnosed With RCC, No.	Timing of Death After Diagnosis, No. (%)				
			All Years	<1 year	1 to <5 years	5 to <10 years	≥10 years
All	Stage						
	I/II	74,733 (70.4%)	18,476 (46.62%)	2,164 (17.87%)	8,106 (48.86%)	6,402 (73.80%)	1,804 (80.04%)
	III	15,058 (14.2%)	6,612 (16.68%)	1,224 (10.11%)	3,378 (20.36%)	1,648 (19.00%)	362 (16.06%)
	IV	16,327 (15.4%)	14,542 (36.69%)	8,724 (72.03%)	5,105 (30.77%)	625 (7.20%)	88 (3.90%)
I/II	Sex						
	Male	45,641 (61.07%)	11,676 (63.20%)	1,352 (62.48%)	51,89 (64.01%)	4,017 (62.75%)	1,118 (61.97%)
	Female	28,859 (38.62%)	6,800 (36.80%)	812 (37.52%)	2,917 (35.99%)	2,385 (37.25%)	686 (38.03%)
	Age, years						
	<65	45,874 (61.38%)	6,766 (36.62%)	629 (29.07%)	2,976 (36.71%)	2,402 (37.52%)	759 (42.07%)
	≥65	28,859 (38.62%)	11,710 (63.38%)	1,535 (70.93%)	5,130 (63.29%)	4,000 (62.48%)	1,045 (57.93%)
	Race						
	White	60,931 (81.53%)	15,005 (81.21%)	1,711 (79.07%)	6,547 (80.77%)	5,281 (82.49%)	1,466 (81.26%)
	Black	9,421 (12.61%)	2,570 (13.91%)	344 (15.90%)	1,173 (14.47%)	820 (12.81%)	233 (12.92%)
	Other	4,381 (5.86%)	901 (4.88%)	109 (5.04%)	386 (4.76%)	301 (4.70%)	105 (5.82%)
	Marital status						
	Married	45,378 (60.72%)	9,864 (53.39%)	985 (45.52%)	4,191 (51.70%)	3,611 (56.40%)	1,077 (59.70%)
	Never married	11,458 (15.33%)	2,505 (13.56%)	332 (15.34%)	1,172 (14.46%)	773 (12.07%)	228 (12.64%)
	Other	17,897 (23.95%)	6,107 (33.05%)	847 (39.14%)	2,743 (33.84%)	2,018 (31.52%)	499 (27.66%)
	Surgery						
	Yes	69,766 (93.35%)	15,433 (83.53%)	1,274 (58.87%)	6,544 (80.73%)	5,884 (91.91%)	1,731 (95.95%)
	No	4,967 (6.65%)	3,043 (16.47%)	890 (41.13%)	1,562 (19.27%)	518 (8.09%)	73 (4.05%)
	Radiation						
	Yes	197 (0.26%)	125 (0.68%)	20 (0.92%)	62 (0.76%)	35 (0.55%)	8 (0.44%)
	No	74,536 (99.74%)	18,351 (99.32%)	2,144 (99.08%)	8,044 (99.24%)	6367, (99.45%)	1,796 (99.56%)
	Chemotherapy						
	Yes	586 (0.78%)	303 (1.64%)	62 (2.87%)	169 (2.08%)	63 (0.98%)	9 (0.50%)
	No	74,147 (99.22%)	18,173 (98.36%)	2,102 (97.13%)	7,937 (97.92%)	6,339 (99.02%)	1,795 (99.50%)
	Year of diagnosis						
	2004–2007	20,681 (27.67%)	8,079 (43.73%)	688 (31.79%)	2,528 (31.19%)	3,129 (48.88%)	1,734 (96.12%)
	2008–2011	25,486 (34.10%)	6,544 (35.42%)	720 (33.27%)	2,841 (35.05%)	2,913 (45.50%)	70 (3.88%)
	2012–2015	28,566 (38.22%)	3,853 (20.85%)	756 (34.94%)	2,737 (33.77%)	360 (5.62%)	0 (0.00%)
	Histology						
	Clear cell	57,395 (76.80%)	14,477 (78.36%)	1,671 (77.22%)	6,320 (77.97%)	5,054 (78.94%)	1,432 (79.38%)
	Papillary	9,492 (12.70%)	2,220 (12.02%)	233 (10.77%)	1,006 (12.41%)	773 (12.07%)	208 (11.53%)
	Chromophobe	4,608 (6.17%)	641 (3.47%)	52 (2.40%)	245 (3.02%)	254 (3.97%)	90 (4.99%)
	Other	3,238 (4.33%)	1,138 (6.16%)	208 (9.61%)	535 (6.60%)	321 (5.01%)	74 (4.10%)
III	Sex						
	Male	10,315 (68.50%)	4,519 (68.35%)	790 (64.54%)	2,331 (69.01%)	1,145 (69.48%)	253 (69.89%)
	Female	4,743 (31.50%)	2,093 (31.65%)	434 (35.46%)	1,047 (30.99%)	503 (30.52%)	109 (30.11%)
	Age, years						
	<65	8,139 (54.05%)	2,805 (42.42%)	485 (39.62%)	1,450 (42.92%)	717 (43.51%)	153 (42.27%)
	≥65	6,919 (45.95%)	3,807 (57.58%)	739 (60.38%)	1,928 (57.08%)	931 (56.49%)	209 (57.73%)
	Race						
	White	12,919 (85.79%)	5,641 (85.31%)	981 (80.15%)	2,890 (85.55%)	1,442 (87.50%)	328 (90.61%)
	Black	1,180 (7.84%)	591 (8.94%)	164 (13.40%)	289 (8.56%)	121 (7.34%)	17 (4.70%)
	Other	959 (6.37%)	380 (5.75%)	79 (6.45%)	199 (5.89%)	85 (5.16%)	17 (4.70%)
	Marital status						
	Married	9,495 (63.06%)	3,944 (59.65%)	667 (54.49%)	1,993 (59.00%)	1,041 (63.17%)	243 (67.13%)
	Never married	2,099 (13.94%)	904 (13.67%)	172 (14.05%)	481 (14.24%)	207 (12.56%)	44 (12.15%)
	Other	3,464 (23.00%)	1,764 (26.68%)	385 (31.45%)	904 (26.76%)	400 (24.27%)	75 (20.72%)
	Surgery						
	Yes	14,567 (96.74%)	6,190 (93.62%)	1,008 (82.35%)	3,209 (95.00%)	1,615 (98.00%)	358 (98.90%)
	No	491 (3.26%)	422 (6.38%)	216 (17.65%)	169 (5.00%)	33 (2.00%)	4 (1.10%)
	Radiation						
	Yes	231 (1.53%)	183 (2.77%)	47 (3.84%)	105 (3.11%)	26 (1.58%)	5 (1.38%)
	No	14,827 (98.47%)	6,429 (97.23%)	1,177 (96.16%)	3,273 (96.89%)	1,622 (98.42%)	357 (98.62%)
	Chemotherapy						
	Yes	1,002 (6.65%)	634 (9.59%)	167 (13.64%)	360 (10.66%)	95 (5.76%)	12 (3.31%)
	No	14,056 (93.35%)	5,978 (90.41%)	1,057 (86.36%)	3,018 (89.34%)	1,553 (94.24%)	350 (96.69%)
	Year of diagnosis						
	2004–2007	4,310 (28.62%)	2,697 (40.79%)	414 (33.82%)	1,110 (32.86%)	830 (50.36%)	343 (94.75%)
	2008–2011	4,974 (33.03%)	2,303 (34.83%)	418 (34.15%)	1,151 (34.07%)	715 (43.39%)	19 (5.25%)
	2012–2015	5,774 (38.35%)	1,612 (24.38%)	392 (32.03%)	1,117 (33.07%)	103 (6.25%)	0 (0.00%)
	Histology						
	Clear cell	12,400 (82.35%)	5,457 (82.53%)	938 (76.63%)	2,785 (82.45%)	1,429 (86.71%)	305 (84.25%)
	Papillary	1,072 (7.12%)	442 (6.68%)	82 (6.70%)	241 (7.13%)	90 (5.46%)	29 (8.01%)
	Chromophobe	697 (4.63%)	161 (2.43%)	17 (1.39%)	78 (2.31%)	52 (3.16%)	14 (3.87%)
	Other	889 (5.90%)	552 (8.35%)	187 (15.28%)	274 (8.11%)	77 (4.67%)	14 (3.87%)
IV	Sex						
	Male	11,064 (67.77%)	9,822 (67.54%)	5,721 (65.58%)	3,598 (70.48%)	437 (69.92%)	66 (75.00%)
	Female	5,263 (32.23%)	4,720 (32.46%)	3,003 (34.42%)	1,507 (29.52%)	188 (30.08%)	22 (25.00%)
	Age, years						
	<65	8,846 (54.18%)	7,679 (52.81%)	4,351 (49.87%)	2,893 (56.67%)	380 (60.80%)	55 (62.50%)
	≥65	7,481 (45.82%)	6,863 (47.19%)	4,373 (50.13%)	2,212 (43.33%)	245 (39.20%)	33 (37.50%)
	Race						
	White	13,500 (82.69%)	12,003 (82.54%)	7,137 (81.81%)	4,255 (83.35%)	538 (86.08%)	73 (82.95%)
	Black	1,709 (10.47%)	1,563 (10.75%)	1,034 (11.85%)	483 (9.46%)	36 (5.76%)	10 (11.36%)
	Other	1,118 (6.85%)	976 (6.71%)	553 (6.34%)	367 (7.19%)	51 (8.16%)	5 (5.68%)
	Marital status						
	Married	9,495 (58.16%)	8,374 (57.58%)	4,845 (55.54%)	3,074 (60.22%)	396 (63.36%)	59 (67.05%)
	Never married	2,580 (15.80%)	2,282 (15.69%)	1,433 (16.43%)	761 (14.91%)	81 (12.96%)	7 (7.95%)
	Other^1^	4,252 (26.04%)	3,886 (26.72%)	2,446 (28.04%)	1,270 (24.88%)	148 (23.68%)	22 (25.00%)
	Surgery						
	Yes	7,409 (45.38%)	8,504 (58.48%)	2,549 (29.22%)	2,926 (57.32%)	489 (78.24%)	74 (84.09%)
	No	8,918 (54.62%)	6,038 (41.52%)	6,175 (70.78%)	2,179 (42.68%)	136 (21.76%)	14 (15.91%)
	Radiation						
	Yes	4,529 (27.74%)	4,273 (29.38%)	2,678 (30.70%)	1,471 (28.81%)	112 (17.92%)	12 (13.64%)
	No	11,798 (72.26%)	10,269 (70.62%)	6,046 (69.30%)	3,634 (71.19%)	513 (82.08%)	76 (86.36%)
	Chemotherapy						
	Yes	8,572 (52.50%)	7,039 (48.40%)	3,935 (45.11%)	2,857 (55.96%)	221 (35.36%)	26 (29.55%)
	No	7,755 (47.50%)	7,503 (51.60%)	4,789 (54.89%)	2,248 (44.04%)	404 (64.64%)	62 (70.45%)
	Year of diagnosis						
	2004–2007	4,960 (30.38%)	4,678 (32.17%)	2,742 (31.43%)	1,537 (30.11%)	313 (50.08%)	86 (97.73%)
	2008–2011	5,383 (32.97%)	4,940 (33.97%)	2,916 (33.43%)	1,753 (34.34%)	269 (43.04%)	2 (2.27%)
	2012–2015	5,984 (36.65%)	4,924 (33.86%)	3,066 (35.14%)	1,815 (35.55%)	43 (6.88%)	0 (0.00%)
	Histology						
	Clear cell	13,337 (81.69%)	11,875 (81.66%)	6,988 (80.10%)	4,297 (84.17%)	522 (83.52%)	68 (77.27%)
	Papillary	698 (4.28%)	612 (4.21%)	304 (3.48%)	264 (5.17%)	37 (5.92%)	7 (7.95%)
	Chromophobe	145 (0.89%)	112 (0.77%)	46 (0.53%)	47 (0.92%)	14 (2.24%)	5 (5.68%)
	Other	2,147 (13.15%)	1,943 (13.36%)	1,386 (15.89%)	497 (9.74%)	52 (8.32%)	8 (9.09%)

Other^1^ includes divorced, separated, widowed, and unmarried or domestic partner.

### Causes of Death for Patients With Stage I/II RCC

Most deaths in patients with stage I or stage II disease occurred either within 1–5 years (*n* = 8,116 [43.93%]) or 5–10 years (*n* = 6,459 [34.96%]) (**Table 1**). In the stage I/II patient cohort, deaths from RCC accounted for 23.13% of all deaths (*n* = 4,273) and the proportion decreased gradually with the extension of survival time (**Table 2**). Noncancer causes of death (*n* = 11,235 [60.81%]) and non-RCC deaths (*n* = 2,853 [15.44%]) were 3.94-fold more frequent (60.81% vs. 15.44%; **Table 2**). Heart disease was the most common noncancer cause of death (*n* = 3,718 [20.12% of all-cause deaths]), and the proportion increased with the extension of survival time (**Figure 2A**). The most common causes of non-RCC cancer deaths were respiratory system and digestive system cancers (*n* = 803 [4.35%] for respiratory system cancer and *n* = 815 [4.41%] for digestive system cancer). Over the whole follow-up period, the risk of death was greater than that in the general population (SMR, 1.54; 95% CI, 1.52–1.56), with the highest risk observed within the first year after RCC diagnosis (SMR, 1.81; 95% CI, 1.74–1.89), the risk levels gradually became stable (SMR, 1.51 for 1 to <5 years; SMR, 1.52 for 5 to <10 years; SMR, 1.51 for ≥10 years). The risk of almost all deaths increased except for breast tumors (SMR, 0.38; 95% CI, 0.28–0.5) and Alzheimer’s disease (SMR, 0.84; 95% CI, 0.75–0.93) (**Table 1**). Ten or more years after the diagnosis of RCC, RCC-specific mortality was still elevated (SMR, 46.01; 95% CI, 40.95–51.52).

**Table 2:** Causes of death for patients with stage I/II RCC.

	Timing of Death After Diagnosis									
	All Years		<1 year		1 to <5 years		5 to <10 years		≥10 years	
Cause of Death	No. (%)	SMR (95% CI)	No. (%)	SMR (95% CI)	No. (%)	SMR (95% CI)	No. (%)	SMR (95% CI)	No. (%)	SMR (95% CI)
All causes of death	18,476 (100.00%)	1.54# (1.52–1.56)	2,069 (100.00%)	1.81# (1.74–1.89)	8,116 (100.00%)	1.51# (1.48–1.54)	6,459 (100.00%)	1.52# (1.48–1.56)	1,832 (100.00%)	1.51# (1.44–1.58)
All malignant cancers	7,126 (38.57%)	2.45# (2.4–2.51)	844 (40.79%)	2.83# (2.64–3.03)	3,416 (42.09%)	2.53# (2.45–2.62)	2,234 (34.59%)	2.25# (2.16–2.34)	632 (34.50%)	2.39# (2.21–2.58)
Oral cavity/pharynx/eye/endocrine	51 (0.28%)	0.84 (0.62–1.1)	5 (0.24%)	0.81 (0.26–1.9)	25 (0.31%)	0.89 (0.57–1.31)	15 (0.23%)	0.72 (0.4–1.18)	6 (0.33%)	1.07 (0.39–2.32)
Digestive system	815 (4.41%)	1.08# (1.01–1.16)	80 (3.87%)	1.04 (0.83–1.3)	327 (4.03%)	0.93 (0.84–1.04)	304 (4.71%)	1.17# (1.04–1.31)	104 (5.68%)	1.50# (1.23–1.82)
Respiratory system	803 (4.35%)	0.97 (0.9–1.04)	51 (2.46%)	0.57# (0.42–0.74)	361 (4.45%)	0.92 (0.82–1.02)	307 (4.75%)	1.11 (0.99–1.24)	84 (4.59%)	1.2 (0.96–1.49)
Bones and joints/soft tissue/skin	70 (0.38%)	0.81 (0.63–1.02)	9 (0.43%)	1.04 (0.48–1.98)	26 (0.32%)	0.65# (0.43–0.96)	25 (0.39%)	0.83 (0.54–1.23)	10 (0.55%)	1.23 (0.59–2.26)
Breast	51 (0.28%)	0.38# (0.28–0.5)	2 (0.10%)	0.14# (0.02–0.51)	13 (0.16%)	0.21# (0.11–0.35)	25 (0.39%)	0.55# (0.36–0.82)	11 (0.60%)	0.93 (0.47–1.67)
Genital system	183 (0.99%)	0.61# (0.53–0.71)	16 (0.77%)	0.55# (0.32–0.9)	81 (1.00%)	0.60# (0.48–0.74)	58 (0.90%)	0.55# (0.42–0.71)	28 (1.53%)	0.94 (0.63–1.36)
Kidney and renal pelvis	4,273 (23.13%)	59.77# (57.99–61.59)	584 (28.23%)	79.88# (73.53–86.63)	2,189 (26.97%)	66.01# (63.27–68.83)	1,200 (18.58%)	49.00# (46.26–51.85)	300 (16.38%)	46.01# (40.95–51.52)
Other urinary organs	98 (0.53%)	1.04 (0.85–1.27)	7 (0.34%)	0.81 (0.33–1.67)	56 (0.69%)	1.34# (1.01–1.74)	29 (0.45%)	0.86 (0.57–1.23)	6 (0.33%)	0.61 (0.22–1.33)
Brain/other nervous system	98 (0.53%)	1.48# (1.2–1.81)	7 (0.34%)	1.03 (0.41–2.11)	46 (0.57%)	1.49# (1.09–1.99)	36 (0.56%)	1.61# (1.12–2.22)	9 (0.49%)	1.52 (0.7–2.89)
Lymph/blood	315 (1.70%)	1.1 (0.98–1.23)	45 (2.17%)	1.58# (1.15–2.11)	129 (1.59%)	0.99 (0.82–1.17)	110 (1.70%)	1.1 (0.91–1.33)	31 (1.69%)	1.14 (0.78–1.62)
Miscellaneous malignant cancer	369 (2.00%)	1.69# (1.52–1.87)	38 (1.84%)	1.71# (1.21–2.35)	163 (2.01%)	1.62# (1.38–1.89)	125 (1.94%)	1.66# (1.38–1.98)	43 (2.35%)	2.10# (1.52–2.83)
*In situ*, benign or unknown behavior neoplasm	115 (0.62%)	1.51# (1.25–1.81)	14 (0.68%)	1.98# (1.08–3.33)	51 (0.63%)	1.51# (1.12–1.98)	43 (0.67%)	1.57# (1.13–2.11)	7 (0.38%)	0.89 (0.36–1.83)
Noncancer	11,235 (60.81%)	1.25# (1.22–1.27)	1,211 (58.53%)	1.45# (1.37–1.53)	4,649 (57.28%)	1.16# (1.13–1.2)	4,182 (64.75%)	1.29# (1.25–1.33)	1,193 (65.12%)	1.27# (1.2–1.34)
Infections	446 (2.41%)	1.50# (1.36–1.64)	59 (2.85%)	1.96# (1.49–2.53)	193 (2.38%)	1.41# (1.22–1.62)	155 (2.40%)	1.51# (1.28–1.77)	39 (2.13%)	1.4 (1–1.91)
Diabetes mellitus	721 (3.90%)	1.91# (1.77–2.05)	92 (4.45%)	2.45# (1.98–3.01)	309 (3.81%)	1.78# (1.59–1.99)	257 (3.98%)	1.95# (1.72–2.21)	63 (3.44%)	1.74# (1.34–2.22)
Alzheimer’s (ICD-9 and 10 only)	363 (1.96%)	0.84# (0.75–0.93)	20 (0.97%)	0.64# (0.39–0.98)	114 (1.40%)	0.66# (0.54–0.79)	170 (2.63%)	1 (0.86–1.17)	59 (3.22%)	1.01 (0.77–1.3)
Diseases of heart	3,718 (20.12%)	1.23# (1.19–1.27)	411 (19.86%)	1.42# (1.29–1.56)	1,567 (19.31%)	1.16# (1.1–1.22)	1,350 (20.90%)	1.26# (1.19–1.33)	390 (21.29%)	1.26# (1.14–1.39)
Hypertension without heart disease	320 (1.73%)	2.25# (2.01–2.52)	30 (1.45%)	2.46# (1.66–3.51)	121 (1.49%)	1.97# (1.64–2.36)	126 (1.95%)	2.40# (2–2.86)	43 (2.35%)	2.70# (1.96–3.64)
Cerebrovascular diseases	710 (3.84%)	1.11# (1.03–1.19)	75 (3.62%)	1.26 (0.99–1.58)	317 (3.91%)	1.12# (1–1.25)	237 (3.67%)	1.03 (0.9–1.17)	81 (4.42%)	1.18 (0.94–1.47)
Diseases of arteries	154 (0.83%)	1.21# (1.02–1.41)	25 (1.21%)	1.91# (1.23–2.82)	57 (0.70%)	0.97 (0.74–1.26)	59 (0.91%)	1.35# (1.03–1.74)	13 (0.71%)	1.08 (0.57–1.84)
Pneumonia and influenza	1,022 (5.53%)	1.01 (0.95–1.08)	94 (4.54%)	1 (0.81–1.22)	414 (5.10%)	0.92 (0.83–1.01)	419 (6.49%)	1.16# (1.05–1.28)	95 (5.19%)	0.92 (0.74–1.12)
Digestive	231 (1.25%)	1.41# (1.23–1.6)	31 (1.50%)	1.78# (1.21–2.53)	110 (1.36%)	1.42# (1.16–1.71)	76 (1.18%)	1.39# (1.1–1.74)	14 (0.76%)	1 (0.55–1.68)
Nephritis/nephrotic syndrome/nephrosis	716 (3.88%)	2.95# (2.74–3.18)	78 (3.77%)	3.43# (2.71–4.29)	287 (3.54%)	2.65# (2.35–2.97)	266 (4.12%)	3.07# (2.71–3.46)	85 (4.64%)	3.42# (2.73–4.23)
Pregnancy/childbirth/puerperium	28 (0.15%)	2.27# (1.51–3.28)	1 (0.05%)	0.74 (0.02–4.15)	11 (0.14%)	1.87 (0.93–3.34)	12 (0.19%)	2.94# (1.52–5.14)	4 (0.22%)	3.83# (1.04–9.8)
Symptoms, signs, and ill-defined conditions	150 (0.81%)	1.16 (0.98–1.36)	15 (0.72%)	1.23 (0.69–2.04)	65 (0.80%)	1.12 (0.86–1.43)	60 (0.93%)	1.29 (0.98–1.65)	10 (0.55%)	0.82 (0.39–1.5)
Accidents/adverse effects/homicide/legal intervention	468 (2.53%)	1.13# (1.03–1.24)	50 (2.42%)	1.21 (0.9–1.59)	210 (2.59%)	1.1 (0.96–1.26)	159 (2.46%)	1.11 (0.95–1.3)	49 (2.67%)	1.24 (0.91–1.63)
Suicide/self-inflicted injury	105 (0.57%)	1.01 (0.82–1.22)	8 (0.39%)	0.68 (0.3–1.35)	50 (0.62%)	0.99 (0.73–1.3)	38 (0.59%)	1.12 (0.8–1.54)	9 (0.49%)	1.08 (0.5–2.06)
Other cause of death	2,083 (11.27%)	1.10# (1.06–1.15)	222 (10.73%)	1.38# (1.2–1.57)	824 (10.15%)	1.01 (0.95–1.09)	798 (12.35%)	1.14# (1.06–1.22)	239 (13.05%)	1.14 (1–1.29)

#p < 0.05; SMRs, standardized mortality ratios; CI, confidence interval.

**Figure 2:**
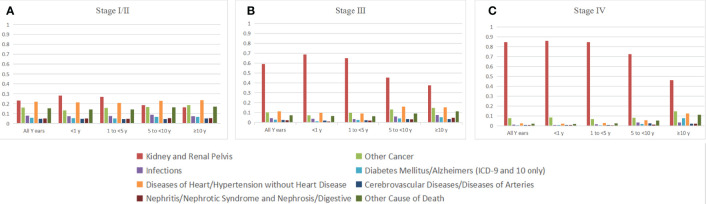
Causes of death by stage after the diagnosis of RCC; **(A)** stage I/II, **(B)**stage III,**(C)** stage RCC.

### Causes of Death for Patients With Stage III RCC

Of the 15,058 patients with stage III RCC, 6,612 (43.91%) died within the follow-up period, 4,574 (30.38%) of whom died from malignant tumors, 3,912 (25.98%) died from RCC, and 2,014 (13.37%) died from noncancer causes. Heart disease was the most common noncancer cause of death (*n* = 708 [10.71% of all deaths]), and miscellaneous malignant cancer was the most common cause of other cancer deaths (*n* = 162 [2.45%]) (**Figure 2B**). Over the entire follow-up period, the mortality rate was greater than that of the general population (SMR, 2.93; 95% CI, 2.86–3.00). The SMR was the highest in the first year of diagnosis (SMR, 4.54; 95% CI, 4.29–4.81), and the risk levels gradually declined (SMR, 3.15, 1 to <5 years; SMR, 2.30 for 5 to <10 years; SMR, 1.88 for ≥10 years).

### Causes of Death for Patients With Stage IV RCC

Most patients (94.99%) with stage IV RCC died within 5 years of the initial diagnosis (8,499 [58.44%] within the first year and 5,315 [36.55%] between 1 and 5 years; **Table 1**). RCC was the leading cause of death (*n* = 12,310 [84.65%]), with other cancers (*n* = 1,122 [7.72%]) and noncancer causes (*n* = 1,110 [7.63%]) accounting for only a small proportion (**Table 4**). Men with stage IV RCC were at a higher risk than the general population (SMR, 21.84; 95% CI, 21.49–22.20). The risk of death from RCC was substantial over all follow-up years (SMR, 2,889.25; 95% CI, 2,838.43–2,940.75) (**Figure 2C**). Heart disease was the most common noncancer cause of death (*n* = 328 [2.23%]). In contrast to other stages, stage IV patients also had a higher risk of suicide (SMR, 3.46; 95% CI, (2.17–5.24).

**Table 3:** Causes of death for patients with stage III RCC.

	Timing of Death After Diagnosis									
	All Years		<1 year		1 to <5 years		5 to <10 years		≥10 years	
Cause of Death	No. (%)	SMR (95% CI)	No. (%)	SMR (95% CI)	No. (%)	SMR (95% CI)	No. (%)	SMR (95% CI)	No. (%)	SMR (95% CI)
All causes of death	6,612 (100.00%)	2.93# (2.86–3)	1,174 (100.00%)	4.54# (4.29–4.81)	3,400 (100.00%)	3.15# (3.05–3.26)	1,672 (100.00%)	2.30# (2.19–2.41)	366 (100.00%)	1.88# (1.69–2.08)
All malignant cancers	4,574 (69.18%)	8.31# (8.07–8.55)	888 (75.64%)	13.07# (12.23–13.96)	2,530 (74.41%)	9.33# (8.97–9.7)	968 (57.89%)	5.70# (5.35–6.07)	188 (51.37%)	4.52# (3.89–5.21)
Oral cavity/pharynx/eye/endocrine	8 (0.12%)	0.69 (0.3–1.36)	2 (0.17%)	1.42 (0.17–5.13)	0 (0.00%)	0.00# (0–0.65)	4 (0.24%)	1.12 (0.3–2.86)	2 (0.55%)	2.25 (0.27–8.13)
Digestive system	142 (2.15%)	1 (0.84–1.18)	18 (1.53%)	1.03 (0.61–1.63)	69 (2.03%)	0.98 (0.77–1.25)	46 (2.75%)	1.05 (0.77–1.39)	9 (2.46%)	0.83 (0.38–1.58)
Respiratory system	176 (2.66%)	1.1 (0.94–1.28)	11 (0.94%)	0.53# (0.26–0.94)	88 (2.59%)	1.1 (0.88–1.35)	60 (3.59%)	1.26 (0.96–1.62)	17 (4.64%)	1.54 (0.9–2.47)
Bones and joints/soft tissue/skin	17 (0.26%)	0.99 (0.57–1.58)	3 (0.26%)	1.46 (0.3–4.26)	9 (0.26%)	1.07 (0.49–2.03)	4 (0.24%)	0.74 (0.2–1.89)	1 (0.27%)	0.74 (0.02–4.1)
Breast	13 (0.20%)	0.65 (0.35–1.12)	1 (0.09%)	0.39 (0.01–2.2)	2 (0.06%)	0.20# (0.02–0.74)	7 (0.42%)	1.16 (0.47–2.4)	3 (0.82%)	2.02 (0.42–5.91)
Genital system	26 (0.39%)	0.47# (0.3–0.68)	1 (0.09%)	0.15# (0–0.86)	10 (0.29%)	0.37# (0.18–0.69)	14 (0.84%)	0.78 (0.43–1.32)	1 (0.27%)	0.21 (0.01–1.2)
Kidney and renal pelvis	3,912 (59.17%)	279.28# (270.6–288.17)	806 (68.65%)	468.31# (436.54–501.79)	2,211 (65.03%)	320.15# (306.94–333.78)	758 (45.33%)	175.32# (163.06–188.26)	137 (37.43%)	129.65# (108.85–153.27)
Other urinary organs	44 (0.67%)	2.28# (1.65–3.05)	2 (0.17%)	0.93 (0.11–3.37)	28 (0.82%)	3.06# (2.03–4.42)	13 (0.78%)	2.05# (1.09–3.5)	1 (0.27%)	0.59 (0.01–3.27)
Brain/other nervous system	22 (0.33%)	1.76# (1.1–2.66)	0 (0.00%)	0 (0–2.37)	14 (0.41%)	2.24# (1.23–3.76)	7 (0.42%)	1.83 (0.74–3.78)	1 (0.27%)	1.09 (0.03–6.08)
Lymph/blood	52 (0.79%)	0.93 (0.69–1.22)	8 (0.68%)	1.2 (0.52–2.36)	27 (0.79%)	0.99 (0.66–1.45)	13 (0.78%)	0.74 (0.39–1.26)	4 (1.09%)	0.9 (0.24–2.3)
Miscellaneous malignant cancer	162 (2.45%)	3.86# (3.29–4.51)	36 (3.07%)	7.05# (4.94–9.76)	72 (2.12%)	3.51# (2.75–4.42)	42 (2.51%)	3.22# (2.32–4.36)	12 (3.28%)	3.66# (1.89–6.4)
*In situ*, benign or unknown behavior neoplasm	24 (0.36%)	1.60# (1.03–2.38)	3 (0.26%)	1.8 (0.37–5.26)	9 (0.26%)	1.27 (0.58–2.41)	9 (0.54%)	1.83 (0.84–3.48)	3 (0.82%)	2.28 (0.47–6.66)
Noncancer	2,014 (30.46%)	1.19# (1.14–1.24)	283 (24.11%)	1.50# (1.33–1.68)	861 (25.32%)	1.08# (1.01–1.15)	695 (41.57%)	1.26# (1.17–1.35)	175 (47.81%)	1.15 (0.99–1.33)
Infections	95 (1.44%)	1.78# (1.44–2.17)	25 (2.13%)	3.89# (2.52–5.74)	32 (0.94%)	1.23 (0.84–1.73)	28 (1.67%)	1.67# (1.11–2.41)	10 (2.73%)	2.34# (1.12–4.31)
Diabetes mellitus	130 (1.97%)	1.87# (1.57–2.22)	13 (1.11%)	1.57 (0.84–2.69)	61 (1.79%)	1.81# (1.38–2.32)	43 (2.57%)	1.97# (1.42–2.65)	13 (3.55%)	2.33# (1.24–3.99)
Alzheimer’s (ICD-9 and 10 only)	61 (0.92%)	0.75# (0.58–0.97)	3 (0.26%)	0.42 (0.09–1.23)	26 (0.76%)	0.74 (0.49–1.09)	25 (1.50%)	0.86 (0.56–1.27)	7 (1.91%)	0.72 (0.29–1.48)
Diseases of heart	708 (10.71%)	1.23# (1.14–1.32)	109 (9.28%)	1.64# (1.35–1.98)	298 (8.76%)	1.09 (0.97–1.22)	249 (14.89%)	1.34# (1.18–1.52)	52 (14.21%)	1.03 (0.77–1.35)
Hypertension without heart disease	41 (0.62%)	1.63# (1.17–2.2)	4 (0.34%)	1.54 (0.42–3.93)	13 (0.38%)	1.12 (0.6–1.92)	20 (1.20%)	2.34# (1.43–3.61)	4 (1.09%)	1.63 (0.44–4.16)
Cerebrovascular diseases	146 (2.21%)	1.23# (1.04–1.44)	20 (1.70%)	1.5 (0.92–2.32)	65 (1.91%)	1.16 (0.9–1.48)	50 (2.99%)	1.29 (0.95–1.69)	11 (3.01%)	1 (0.5–1.79)
Diseases of arteries	22 (0.33%)	0.89 (0.56–1.35)	1 (0.09%)	0.33 (0.01–1.83)	12 (0.35%)	1 (0.52–1.75)	7 (0.42%)	0.92 (0.37–1.9)	2 (0.55%)	1.02 (0.12–3.69)
Pneumonia and influenza	191 (2.89%)	0.97 (0.84–1.12)	19 (1.62%)	0.86 (0.52–1.34)	81 (2.38%)	0.87 (0.69–1.08)	73 (4.37%)	1.15 (0.9–1.44)	18 (4.92%)	1.06 (0.63–1.67)
Digestive	48 (0.73%)	1.60# (1.18–2.13)	5 (0.43%)	1.3 (0.42–3.04)	21 (0.62%)	1.39 (0.86–2.12)	17 (1.02%)	1.92# (1.12–3.07)	5 (1.37%)	2.43 (0.79–5.67)
Nephritis/nephrotic syndrome/nephrosis	94 (1.42%)	2.10# (1.69–2.57)	6 (0.51%)	1.19 (0.44–2.6)	43 (1.26%)	2.02# (1.46–2.72)	33 (1.97%)	2.26# (1.55–3.17)	12 (3.28%)	3.07# (1.59–5.36)
Pregnancy/childbirth/puerperium	6 (0.09%)	2.84# (1.04–6.17)	2 (0.17%)	7.26 (0.88–26.23)	3 (0.09%)	2.8 (0.58–8.19)	1 (0.06%)	1.61 (0.04–8.95)	0 (0.00%)	0 (0–25.11)
Symptoms, signs, and ill-defined conditions	35 (0.53%)	1.47# (1.03–2.05)	8 (0.68%)	3.01# (1.3–5.92)	19 (0.56%)	1.68# (1.01–2.63)	8 (0.48%)	1.02 (0.44–2.01)	0 (0.00%)	0 (0–1.87)
Accidents/adverse effects/homicide/legal intervention	64 (0.97%)	0.86 (0.66–1.1)	8 (0.68%)	0.91 (0.39–1.8)	34 (1.00%)	0.94 (0.65–1.32)	20 (1.20%)	0.86 (0.53–1.33)	2 (0.55%)	0.33 (0.04–1.18)
Suicide/self-inflicted injury	13 (0.20%)	0.67 (0.36–1.15)	1 (0.09%)	0.39 (0.01–2.17)	10 (0.29%)	1.01 (0.48–1.86)	2 (0.12%)	0.36 (0.04–1.29)	0 (0.00%)	0 (0–2.93)
Other cause of death	360 (5.44%)	1.02 (0.91–1.13)	59 (5.03%)	1.62# (1.23–2.09)	143 (4.21%)	0.88 (0.74–1.03)	119 (7.12%)	0.99 (0.82–1.18)	39 (10.66%)	1.14 (0.81–1.56)

#p < 0.05; SMRs, standardized mortality ratios; CI, confidence interval.

**Table 4:** Causes of death for patients with stage IV RCC.

	Timing of Death After Diagnosis									
	All Years		<1 year		1 to <5 years		5 to <10 years		≥10 years	
Cause of Death	No. (%)	SMR (95% CI)	No. (%)	SMR (95% CI)	No. (%)	SMR (95% CI)	No. (%)	SMR (95% CI)	No. (%)	SMR (95% CI)
All causes of death	14,542 (100.00%)	21.84# (21.49–22.2)	8,499 (100.00%)	45.20# (44.24–46.17)	5,315 (100.00%)	16.25# (15.82–16.69)	639 (100.00%)	5.23# (4.84–5.66)	89 (100.00%)	3.11# (2.5–3.83)
All malignant cancers	13,396 (92.12%)	80.42# (79.07–81.8)	7,988 (93.99%)	165.57# (161.95–169.24)	4,841 (91.08%)	58.04# (56.42–59.7)	514 (80.44%)	17.91# (16.39–19.52)	53 (59.55%)	8.54# (6.4–11.17)
Oral cavity/pharynx/eye/endocrine	12 (0.08%)	3.34# (1.73–5.84)	9 (0.11%)	8.92# (4.08–16.93)	2 (0.04%)	1.1 (0.13–3.97)	0 (0.00%)	0 (0–5.91)	1 (1.12%)	7.41 (0.19–41.29)
Digestive system	105 (0.72%)	2.40# (1.97–2.91)	59 (0.69%)	4.73# (3.6–6.1)	38 (0.71%)	1.73# (1.22–2.37)	6 (0.94%)	0.79 (0.29–1.72)	2 (2.25%)	1.23 (0.15–4.46)
Respiratory system	226 (1.55%)	4.60# (4.02–5.24)	142 (1.67%)	9.71# (8.18–11.44)	71 (1.34%)	2.87# (2.24–3.62)	10 (1.56%)	1.24 (0.59–2.27)	3 (3.37%)	1.83 (0.38–5.34)
Bones and joints/soft tissue/skin	39 (0.27%)	7.64# (5.43–10.45)	25 (0.29%)	17.40# (11.26–25.69)	11 (0.21%)	4.30# (2.15–7.7)	3 (0.47%)	3.32 (0.68–9.71)	0 (0.00%)	0 (0–17.97)
Breast	7 (0.05%)	1.17 (0.47–2.42)	3 (0.04%)	1.64 (0.34–4.8)	3 (0.06%)	1.03 (0.21–3)	0 (0.00%)	0 (0–3.62)	1 (1.12%)	5 (0.13–27.83)
Genital system	42 (0.29%)	2.54# (1.83–3.44)	25 (0.29%)	5.30# (3.43–7.83)	15 (0.28%)	1.85# (1.03–3.05)	2 (0.31%)	0.67 (0.08–2.43)	0 (0.00%)	0 (0–5.25)
Kidney and renal pelvis	12,310 (84.65%)	2,889.25# (2,838.43–2,940.75)	7,308 (85.99%)	6,003.28# (5,866.42–6,142.52)	4,498 (84.63%)	2,095.36# (2,034.57–2,157.51)	463 (72.46%)	628.07# (572.16–687.97)	41 (46.07%)	257.12# (184.51–348.81)
Other urinary organs	88 (0.61%)	16.05# (12.87–19.77)	59 (0.69%)	39.25# (29.88–50.64)	28 (0.53%)	10.39# (6.9–15.01)	1 (0.16%)	0.97 (0.02–5.43)	0 (0.00%)	0 (0–14.31)
Brain/other nervous system	21 (0.14%)	5.45# (3.37–8.33)	11 (0.13%)	10.09# (5.03–18.05)	7 (0.13%)	3.56# (1.43–7.35)	3 (0.47%)	4.54 (0.94–13.28)	0 (0.00%)	0 (0–26.47)
Lymph/blood	73 (0.50%)	4.45# (3.48–5.59)	34 (0.40%)	7.23# (5–10.1)	29 (0.55%)	3.55# (2.38–5.1)	7 (1.10%)	2.42 (0.97–4.98)	3 (3.37%)	4.56 (0.94–13.34)
Miscellaneous malignant cancer	473 (3.25%)	37.57# (34.26–41.11)	313 (3.68%)	86.16# (76.88–96.25)	139 (2.62%)	22.13# (18.61–26.13)	19 (2.97%)	8.68# (5.22–13.55)	2 (2.25%)	4.11 (0.5–14.85)
*In situ*, benign, or unknown behavior neoplasm	36 (0.25%)	8.44# (5.91–11.68)	21 (0.25%)	17.67# (10.94–27)	14 (0.26%)	6.71# (3.67–11.25)	0 (0.00%)	0 (0–4.63)	1 (1.12%)	5.18 (0.13–28.88)
Noncancer	1,110 (7.63%)	2.24# (2.11–2.38)	490 (5.77%)	3.54# (3.23–3.86)	460 (8.65%)	1.90# (1.73–2.09)	125 (19.56%)	1.35# (1.12–1.61)	35 (39.33%)	1.58# (1.1–2.19)
Infections	65 (0.45%)	3.95# (3.05–5.04)	33 (0.39%)	6.93# (4.77–9.74)	25 (0.47%)	3.06# (1.98–4.52)	6 (0.94%)	2.08 (0.76–4.52)	1 (1.12%)	1.58 (0.04–8.83)
Diabetes mellitus	57 (0.39%)	2.69# (2.04–3.48)	21 (0.25%)	3.49# (2.16–5.33)	22 (0.41%)	2.08# (1.31–3.16)	9 (1.41%)	2.38# (1.09–4.52)	5 (5.62%)	5.95# (1.93–13.88)
Alzheimer’s (ICD-9 and 10 only)	21 (0.14%)	0.98 (0.61–1.5)	5 (0.06%)	0.92 (0.3–2.14)	12 (0.23%)	1.21 (0.63–2.12)	2 (0.31%)	0.43 (0.05–1.55)	2 (2.25%)	1.48 (0.18–5.34)
Diseases of heart	328 (2.26%)	1.92# (1.72–2.14)	157 (1.85%)	3.22# (2.74–3.76)	125 (2.35%)	1.50# (1.25–1.79)	35 (5.48%)	1.12 (0.78–1.56)	11 (12.36%)	1.48 (0.74–2.65)
Hypertension without heart disease	22 (0.15%)	2.96# (1.86–4.48)	8 (0.09%)	4.02# (1.74–7.92)	13 (0.24%)	3.63# (1.93–6.2)	1 (0.16%)	0.67 (0.02–3.73)	0 (0.00%)	0 (0–10.12)
Cerebrovascular diseases	87 (0.60%)	2.51# (2.01–3.09)	31 (0.36%)	3.13# (2.13–4.45)	40 (0.75%)	2.39# (1.71–3.25)	14 (2.19%)	2.16# (1.18–3.62)	2 (2.25%)	1.26 (0.15–4.56)
Diseases of arteries	18 (0.12%)	2.43# (1.44–3.84)	7 (0.08%)	3.15# (1.27–6.5)	9 (0.17%)	2.48# (1.14–4.71)	2 (0.31%)	1.56 (0.19–5.64)	0 (0.00%)	0 (0–12.91)
Pneumonia and influenza	123 (0.85%)	2.20# (1.83–2.62)	51 (0.60%)	3.24# (2.41–4.26)	55 (1.03%)	2.01# (1.51–2.61)	15 (2.35%)	1.45 (0.81–2.4)	2 (2.25%)	0.81 (0.1–2.94)
Digestive	15 (0.10%)	1.55 (0.87–2.55)	4 (0.05%)	1.45 (0.39–3.7)	11 (0.21%)	2.20# (1.1–3.93)	0 (0.00%)	0 (0–2.28)	0 (0.00%)	0 (0–11.65)
Nephritis/nephrotic syndrome/nephrosis	49 (0.34%)	3.70# (2.74–4.89)	22 (0.26%)	5.90# (3.7–8.93)	18 (0.34%)	2.79# (1.65–4.41)	7 (1.10%)	2.83# (1.14–5.83)	2 (2.25%)	3.44 (0.42–12.43)
Pregnancy/childbirth/puerperium	8 (0.06%)	11.54# (4.98–22.74)	3 (0.04%)	14.90# (3.07–43.54)	5 (0.09%)	14.11# (4.58–32.93)	0 (0.00%)	0 (0–32.33)	0 (0.00%)	0 (0–158.67)
Symptoms, signs, and ill-defined conditions	33 (0.23%)	4.65# (3.2–6.53)	19 (0.22%)	9.43# (5.68–14.73)	13 (0.24%)	3.79# (2.02–6.48)	1 (0.16%)	0.73 (0.02–4.09)	0 (0.00%)	0 (0–13.05)
Accidents/adverse effects/homicide/legal intervention	50 (0.34%)	2.20# (1.63–2.9)	16 (0.19%)	2.49# (1.42–4.04)	27 (0.51%)	2.36# (1.56–3.44)	4 (0.63%)	1 (0.27–2.57)	3 (3.37%)	3.24 (0.67–9.48)
Suicide/self-inflicted injury	22 (0.15%)	3.46# (2.17–5.24)	12 (0.14%)	6.55# (3.39–11.45)	9 (0.17%)	2.73# (1.25–5.18)	1 (0.16%)	0.97 (0.02–5.43)	0 (0.00%)	0 (0–18.28)
Other cause of death	212 (1.46%)	2.12# (1.84–2.43)	101 (1.19%)	3.77# (3.07–4.58)	76 (1.43%)	1.57# (1.24–1.97)	28 (4.38%)	1.4 (0.93–2.03)	7 (7.87%)	1.42 (0.57–2.92)

#p < 0.05; SMRs, standardized mortality ratios; CI, confidence interval.

### Subgroup Analysis

Male and female patients had a similar risk of all-cause death, but female patients (SMR, 330.10; 95% CI, 322.25–338.08) had a higher risk of intentional deaths from RCC than male patients (SMR, 198.48; 95% CI, 195.18–201.83) (**Tables S1** and **S2**). Compared with patients aged <65 years, the risk of death of patients aged ≥65 years was higher than that of patients aged <65 years (**Tables S3** and **S4**). Patients of other races have a higher risk of all-cause death than black and white patients (SMR, 4.29; 95% CI, 4.12–4.47 for other races; SMR, 2.57; 95% CI, 2.54–2.59 for white; SMR, 2.85; 95% CI, 2.77–2.93 for black) (**Tables S5**–**S7**). Patients who have never been married have a higher risk of death than those who are married and in other marital statuses, regardless of whether it is a cancer or a noncancer factor (**Tables S8**–**S10**). Noncancer causes of death were lower in patients who underwent surgery than in those who did not (**Tables S11** and **S12**). In contrast, patients who received chemotherapy or radiotherapy showed an increase in noncancer causes of death (**Tables S13**–**S16**). There was no significant difference in SMR between the different years of diagnosis (**Tables S17**–**S19**). The prognosis of patients with other histological types was the worst, and the prognosis of patients with chromophobe histological type was the best (SMR, 1.08; 95% CI, 1.01–1.16 for chromophobe histological type; SMR,4.37; 95% CI, 4.23–4.51 for other histological types) (**Tables S20**–**S23**).

## Discussion

In the U.S., there are more than 320,000 RCC survivors, and the number is still increasing. It is of vital importance, during survivorship, to optimize healthcare management. We stratified our results according to patient characteristics and stage. In the patient cohort with stage I**/**II RCC, non-RCC causes of death were 3.32-fold more frequent (76.87% vs. 23.13%, respectively). In patients with stage III/IV disease, 59.17% (*n* = 3,912) and 84.65% (*n* = 12,310) of the patients died from RCC, respectively, and the risk of death due to most non-RCC causes was still higher than that in the general population. These results mirror those of previous studies (11).

Our study demonstrates the causes of death in RCC patients with different stages and characteristics, which may help clinicians. For example, the prevention and control of noncancer causes and screening for non-kidney cancer can be emphasized.

In patients with RCC, the most common noncancer death was heart disease. In our study, patients with stage IV disease had a higher SMR in heart disease than those with stage I/II and III (SMR, 1.92; 95% CI, 1.72–2.14 for stage IV; SMR, 1.23; 95% CI, 1.14–1.32 for stage III; SMR, 1.23; 95% CI, 1.19–1.27 for stage I/II). In the past decade, several tyrosine kinases and vascular endothelial growth factor inhibitors have been used for first-line therapies in patients with metastatic RCC (13, 14). These drugs greatly improved the survival rate of patients with metastatic RCC. However, cardiotoxicity cannot be ignored (15–17).

Hemocytopenia due to antineoplastic therapy is a common occurrence in clinic (18–21). Neutropenia is independently associated with septicemia (22, 23). In addition, the process of tumor metastasis may damage the immune system, resulting in a higher infection risk in cancer patients (24). In this study, patients with stage IV RCC had a markedly higher risk of death from septicemia, especially those who died within a year (SMR, 3.95; 95% CI, 3.05–5.04 for infections; SMR, 2.20; 95% CI, 1.83–2.62 for pneumonia and influenza). In our study, patients with stage IV disease were at a higher risk of suicide. This outcome is similar to that of Guo et al. (25).

In this study, we found that patients who underwent direct cancer surgery had a lower SMR than those who did not. It seems possible that these results are due to patients undergoing surgery having fewer comorbidity and better health conditions than those who did not.

Patients who were never married or in other marital statuses had a higher risk of all-cause death, kidney cancer-specific death, and other noncancer deaths than married patients. The fact that marriage provides social support may partly explain this finding (14, 26, 27).

However, this study has some limitations. First, our study design was retrospective, which inevitably resulted in a selection bias. We also performed our best to reduce bias. We used strict screening criteria to reduce the selection bias and use SMR to control for age, sex, and ethnic differences, rather than direct mortality to reduce confusion bias. Second, the SEER database lacks important information on treatment strategies and comorbid states, which may cause bias. Third, most of our participants were white, and whether our conclusion can be extended to other races, it still needs to be further verified. Finally, there may be potential misclassification of the cause of death in the SEER database. However, previous studies have shown that this variable is accurate in most situations (28).

## Conclusions

In summary, this study provides the latest and most comprehensive assessment of the causes of death in patients with RCC. Causes of death varied according to patient demographics. Non-RCC causes of death account for more than 3/4 of RCC survivors among patients with stage I/II disease. Patients with stage IV are most likely to die of RCC; however, there is an increased risk of dying from septicemia, and suicide cannot be ignored. Therefore, attention should be paid not only to antineoplastic therapy, but also to the occurrence of other risks.

## Data Availability Statement

The raw data supporting the conclusions of this article will be made available by the authors, without undue reservation.

## Author Contributions

(i) Conception and design: D-DY, W-KC, and C-YW. (ii) Administrative support: M-HZ and PL. (iii) Provision of study materials or patients: All authors. (iv) Collection and assembly of data: D-DY, W-KC, and C-YW. (v) Data analysis and interpretation: All authors. (vi) Manuscript writing: All authors. (vii) Final approval of manuscript: All authors.

## Conflict of Interest

The authors declare that the research was conducted in the absence of any commercial or financial relationships that could be construed as a potential conflict of interest.

## Publisher’s Note

All claims expressed in this article are solely those of the authors and do not necessarily represent those of their affiliated organizations, or those of the publisher, the editors and the reviewers. Any product that may be evaluated in this article, or claim that may be made by its manufacturer, is not guaranteed or endorsed by the publisher.
