# Supplementary material for: Cause of Death During Renal Cell Carcinoma Survivorship: A Contemporary, Population-Based Analysis
Source: Front Oncol. 2022 Jun 2;12:864132. doi: 10.3389/fonc.2022.864132 (PMC9201523; doi:10.3389/fonc.2022.864132)
Supplement: Supplementary file 1 [file Table_1.docx]

Supplementary Table 1. Standardized-mortality ratios (SMRs) for each cause of death following RCC diagnosis in male patients.

|  | | Timing of Death After Diagnosis | | | | | | | | | | | | | | |
| --- | --- | --- | --- | --- | --- | --- | --- | --- | --- | --- | --- | --- | --- | --- | --- | --- |
|  | | All Y ears | | | <1 y | | | 1 to <5 y | | | 5 to <10 y | | | ≥10 y | | |
| All Causes of Death | 26,017 | | 2.68#(2.65-2.72) | 7,621 | | 7.26#(7.1-7.43) | 11,276 | | 2.54#(2.49-2.58) | 5,660 | | 1.72#(1.68-1.77) | 1,460 | | 1.59#(1.51-1.67) |  |
| All Malignant Cancers | 16,892 | | 6.83#(6.72-6.93) | 6,316 | | 22.21#(21.67-22.77) | 7,419 | | 6.37#(6.22-6.51) | 2,555 | | 3.15#(3.03-3.27) | 602 | | 2.83#(2.6-3.06) |  |
| Oral Cavity/Pharynx/eye/endocrine | 51 | | 0.87(0.65-1.14) | 11 | | 1.64(0.82-2.94) | 19 | | 0.68(0.41-1.07) | 15 | | 0.78(0.43-1.28) | 6 | | 1.18(0.43-2.57) |  |
| Digestive System | 746 | | 1.11#(1.03-1.19) | 106 | | 1.38#(1.13-1.67) | 311 | | 0.98(0.88-1.1) | 246 | | 1.12(0.98-1.27) | 83 | | 1.44#(1.15-1.79) |  |
| Respiratory System | 859 | | 1.17#(1.09-1.25) | 142 | | 1.58#(1.33-1.87) | 377 | | 1.07(0.96-1.18) | 266 | | 1.14#(1.01-1.29) | 74 | | 1.29#(1.01-1.61) |  |
| Bones and Joints/soft tissue/skin | 92 | | 1.09(0.88-1.34) | 23 | | 2.45#(1.55-3.68) | 36 | | 0.92(0.64-1.27) | 23 | | 0.82(0.52-1.23) | 10 | | 1.34(0.64-2.46) |  |
| Breast | 5 | | 1.4(0.46-3.28) | 2 | | 5.02(0.61-18.13) | 2 | | 1.2(0.15-4.34) | 1 | | 0.85(0.02-4.71) | 0 | | 0(0-11.82) |  |
| Genital System | 158 | | 0.62#(0.53-0.73) | 25 | | 0.94(0.61-1.38) | 62 | | 0.54#(0.42-0.69) | 49 | | 0.56#(0.42-0.75) | 22 | | 0.88(0.55-1.33) |  |
| Kidney and Renal Pelvis | 13,773 | | 198.48#(195.18-201.83) | 5,661 | | 710.25#(691.87-729) | 6,103 | | 186.32#(181.67-191.05) | 1,678 | | 73.81#(70.32-77.43) | 331 | | 55.80#(49.95-62.14) |  |
| Other Urinary Organs | 159 | | 1.62#(1.38-1.9) | 40 | | 3.95#(2.82-5.38) | 79 | | 1.79#(1.42-2.23) | 36 | | 1.06(0.74-1.47) | 4 | | 0.41(0.11-1.05) |  |
| Brain/Other Nervous System | 89 | | 1.54#(1.23-1.89) | 9 | | 1.34(0.61-2.55) | 40 | | 1.45#(1.04-1.97) | 35 | | 1.86#(1.3-2.59) | 5 | | 1.03(0.33-2.41) |  |
| lymphandblood | 303 | | 1.19#(1.06-1.33) | 60 | | 2.12#(1.61-2.72) | 135 | | 1.14(0.96-1.35) | 83 | | 0.98(0.78-1.21) | 25 | | 1.09(0.71-1.61) |  |
| Miscellaneous Malignant Cancer | 657 | | 3.44#(3.19-3.72) | 237 | | 10.90#(9.56-12.38) | 255 | | 2.85#(2.51-3.23) | 123 | | 1.96#(1.63-2.33) | 42 | | 2.50#(1.8-3.38) |  |
| In situ, benign or unknown behavior neoplasm | 113 | | 1.73#(1.43-2.08) | 27 | | 3.98#(2.62-5.79) | 46 | | 1.56#(1.14-2.08) | 35 | | 1.55#(1.08-2.15) | 5 | | 0.78(0.25-1.82) |  |
| Noncancer | 9,012 | | 1.26#(1.23-1.29) | 1,278 | | 1.69#(1.59-1.78) | 3,811 | | 1.17#(1.14-1.21) | 3,070 | | 1.25#(1.21-1.3) | 853 | | 1.22#(1.14-1.31) |  |
| infections | 395 | | 1.64#(1.48-1.81) | 80 | | 2.90#(2.3-3.61) | 173 | | 1.53#(1.31-1.77) | 112 | | 1.41#(1.16-1.7) | 30 | | 1.42(0.96-2.03) |  |
| Diabetes Mellitus | 570 | | 1.80#(1.65-1.95) | 80 | | 2.29#(1.82-2.86) | 242 | | 1.64#(1.44-1.86) | 192 | | 1.81#(1.56-2.08) | 56 | | 1.93#(1.46-2.5) |  |
| Alzheimers (ICD-9 and 10 only) | 206 | | 0.83#(0.72-0.95) | 11 | | 0.53#(0.27-0.95) | 78 | | 0.77#(0.61-0.96) | 79 | | 0.84(0.66-1.05) | 38 | | 1.18(0.83-1.62) |  |
| Diseases of Heart | 3,105 | | 1.22#(1.18-1.26) | 464 | | 1.68#(1.53-1.84) | 1,325 | | 1.14#(1.08-1.2) | 1,056 | | 1.23#(1.15-1.3) | 260 | | 1.07(0.94-1.21) |  |
| Hypertension without Heart Disease | 222 | | 2.20#(1.92-2.5) | 23 | | 2.33#(1.48-3.5) | 91 | | 2.04#(1.64-2.5) | 74 | | 2.06#(1.62-2.59) | 34 | | 3.18#(2.2-4.45) |  |
| Cerebrovascular Diseases | 549 | | 1.20#(1.1-1.31) | 80 | | 1.66#(1.31-2.06) | 236 | | 1.15#(1.01-1.3) | 169 | | 1.08(0.92-1.26) | 64 | | 1.40#(1.08-1.79) |  |
| Diseases of Arteries | 111 | | 1.07(0.88-1.29) | 17 | | 1.39(0.81-2.23) | 48 | | 0.98(0.73-1.31) | 36 | | 1.06(0.74-1.47) | 10 | | 1.1(0.53-2.02) |  |
| Pneumonia and Influenza | 778 | | 0.97(0.91-1.04) | 99 | | 1.17(0.95-1.42) | 313 | | 0.86#(0.77-0.96) | 304 | | 1.11(0.99-1.24) | 62 | | 0.8(0.62-1.03) |  |
| Digestive | 207 | | 1.34#(1.17-1.54) | 26 | | 1.41(0.92-2.06) | 100 | | 1.34#(1.09-1.63) | 65 | | 1.33#(1.03-1.7) | 16 | | 1.32(0.75-2.14) |  |
| Nephritis\ Nephrotic Syndrome\Nephrosis | 546 | | 2.77#(2.54-3.01) | 68 | | 3.30#(2.56-4.18) | 226 | | 2.53#(2.21-2.89) | 190 | | 2.80#(2.41-3.22) | 62 | | 3.21#(2.46-4.12) |  |
| Pregnancy\Childbirth\Puerperium | 18 | | 1.87#(1.11-2.96) | 2 | | 1.72(0.21-6.21) | 9 | | 1.93(0.88-3.67) | 5 | | 1.65(0.53-3.85) | 2 | | 2.59(0.31-9.37) |  |
| Symptoms, Signs and Ill-Defined Conditions | 135 | | 1.48#(1.24-1.75) | 29 | | 2.95#(1.97-4.23) | 57 | | 1.36#(1.03-1.76) | 43 | | 1.37(0.99-1.85) | 6 | | 0.73(0.27-1.59) |  |
| Accidents\Adverse Effects\Homicide\Legal intervention | 410 | | 1.11#(1-1.22) | 47 | | 1.12(0.82-1.48) | 201 | | 1.15(1-1.32) | 124 | | 1.02(0.85-1.22) | 38 | | 1.16(0.82-1.6) |  |
| Suicide\Self-Inflicted Injury | 116 | | 1.02(0.84-1.22) | 19 | | 1.35(0.81-2.1) | 60 | | 1.07(0.82-1.38) | 31 | | 0.87(0.59-1.24) | 6 | | 0.7(0.26-1.52) |  |
| Other Cause of Death | 1,644 | | 1.17#(1.11-1.23) | 233 | | 1.70#(1.49-1.93) | 652 | | 1.05(0.97-1.13) | 590 | | 1.18#(1.09-1.28) | 169 | | 1.14(0.97-1.33) |  |

Supplementary Table 2. Standardized-mortality ratios (SMRs) for each cause of death following RCC diagnosis in Female patients.

|  | | Timing of Death After Diagnosis | | | | | | | | | | | | | |
| --- | --- | --- | --- | --- | --- | --- | --- | --- | --- | --- | --- | --- | --- | --- | --- |
|  | | All Y ears | | | <1 y | | | 1 to <5 y | | | 5 to <10 y | | | ≥10 y | |
| All Causes of Death | 13,613 | | 2.61#(2.57-2.66) | 4,121 | | 7.66#(7.43-7.9) | 5,555 | | 2.38#(2.32-2.44) | 3,110 | | 1.71#(1.65-1.77) | 827 | | 1.59#(1.48-1.7) |
| All Malignant Cancers | 8,204 | | 7.16#(7-7.31) | 3,404 | | 26.16#(25.28-27.05) | 3,368 | | 6.28#(6.07-6.49) | 1,161 | | 3.05#(2.88-3.23) | 271 | | 2.74#(2.42-3.08) |
| Oral Cavity/Pharynx/eye/endocrine | 20 | | 1.16(0.71-1.8) | 5 | | 2.66(0.86-6.2) | 8 | | 1.01(0.44-1.99) | 4 | | 0.69(0.19-1.77) | 3 | | 1.91(0.39-5.58) |
| Digestive System | 316 | | 1.17#(1.04-1.31) | 51 | | 1.71#(1.27-2.24) | 123 | | 0.98(0.82-1.17) | 110 | | 1.21(0.99-1.46) | 32 | | 1.32(0.9-1.87) |
| Respiratory System | 346 | | 1.13#(1.01-1.25) | 62 | | 1.73#(1.32-2.21) | 143 | | 0.98(0.83-1.16) | 111 | | 1.11(0.91-1.34) | 30 | | 1.2(0.81-1.71) |
| Bones and Joints/soft tissue/skin | 34 | | 1.37(0.95-1.92) | 14 | | 5.09#(2.79-8.55) | 10 | | 0.87(0.42-1.6) | 9 | | 1.08(0.49-2.05) | 1 | | 0.45(0.01-2.51) |
| Breast | 66 | | 0.42#(0.33-0.54) | 4 | | 0.22#(0.06-0.56) | 16 | | 0.22#(0.12-0.35) | 31 | | 0.61#(0.41-0.86) | 15 | | 1.14(0.64-1.88) |
| Genital System | 93 | | 0.78#(0.63-0.96) | 17 | | 1.26(0.73-2.01) | 44 | | 0.79(0.57-1.06) | 25 | | 0.64#(0.41-0.94) | 7 | | 0.7(0.28-1.44) |
| Kidney and Renal Pelvis | 6,722 | | 330.10#(322.25-338.08) | 3,037 | | 1,332.74#(1285.76-1381) | 2,795 | | 295.42#(284.56-306.58) | 743 | | 108.96#(101.27-117.09) | 147 | | 81.45#(68.81-95.73) |
| Other Urinary Organs | 71 | | 3.37#(2.63-4.25) | 28 | | 12.86#(8.55-18.59) | 33 | | 3.49#(2.4-4.9) | 7 | | 0.95(0.38-1.97) | 3 | | 1.45(0.3-4.23) |
| Brain/Other Nervous System | 52 | | 2.12#(1.58-2.78) | 9 | | 3.26#(1.49-6.19) | 27 | | 2.35#(1.55-3.41) | 11 | | 1.35(0.68-2.42) | 5 | | 2.37(0.77-5.52) |
| lymphandblood | 137 | | 1.31#(1.1-1.55) | 27 | | 2.34#(1.54-3.41) | 50 | | 1.04(0.77-1.37) | 47 | | 1.34(0.98-1.78) | 13 | | 1.39(0.74-2.37) |
| Miscellaneous Malignant Cancer | 347 | | 4.21#(3.78-4.68) | 150 | | 16.32#(13.82-19.16) | 119 | | 3.12#(2.58-3.73) | 63 | | 2.28#(1.75-2.92) | 15 | | 2.02#(1.13-3.33) |
| In situ, benign or unknown behavior neoplasm | 62 | | 2.05#(1.58-2.63) | 11 | | 3.52#(1.76-6.29) | 28 | | 2.07#(1.37-2.99) | 17 | | 1.61(0.94-2.58) | 6 | | 2.02(0.74-4.41) |
| Noncancer | 5,347 | | 1.32#(1.29-1.36) | 706 | | 1.75#(1.62-1.88) | 2,159 | | 1.21#(1.16-1.26) | 1,932 | | 1.35#(1.29-1.41) | 550 | | 1.31#(1.21-1.43) |
| infections | 211 | | 1.67#(1.45-1.91) | 37 | | 2.70#(1.9-3.72) | 77 | | 1.32#(1.05-1.66) | 77 | | 1.79#(1.41-2.24) | 20 | | 1.72#(1.05-2.66) |
| Diabetes Mellitus | 338 | | 2.23#(2-2.48) | 46 | | 2.72#(1.99-3.63) | 150 | | 2.14#(1.81-2.51) | 117 | | 2.30#(1.91-2.76) | 25 | | 1.84#(1.19-2.71) |
| Alzheimers (ICD-9 and 10 only) | 239 | | 0.83#(0.73-0.95) | 17 | | 0.73(0.42-1.16) | 74 | | 0.64#(0.5-0.8) | 118 | | 1.08(0.89-1.29) | 30 | | 0.8(0.54-1.14) |
| Diseases of Heart | 1,649 | | 1.35#(1.28-1.41) | 213 | | 1.67#(1.45-1.91) | 665 | | 1.22#(1.12-1.31) | 578 | | 1.35#(1.24-1.47) | 193 | | 1.56#(1.35-1.8) |
| Hypertension without Heart Disease | 161 | | 2.19#(1.87-2.56) | 19 | | 2.74#(1.65-4.27) | 56 | | 1.76#(1.33-2.29) | 73 | | 2.74#(2.15-3.44) | 13 | | 1.61(0.86-2.76) |
| Cerebrovascular Diseases | 394 | | 1.16#(1.05-1.29) | 46 | | 1.34(0.98-1.79) | 186 | | 1.25#(1.07-1.44) | 132 | | 1.11(0.93-1.31) | 30 | | 0.84(0.57-1.2) |
| Diseases of Arteries | 83 | | 1.49#(1.19-1.85) | 16 | | 2.59#(1.48-4.21) | 30 | | 1.18(0.8-1.68) | 32 | | 1.70#(1.16-2.4) | 5 | | 0.96(0.31-2.24) |
| Pneumonia and Influenza | 558 | | 1.21#(1.11-1.31) | 65 | | 1.37#(1.06-1.75) | 237 | | 1.15#(1-1.3) | 203 | | 1.26#(1.09-1.44) | 53 | | 1.16(0.87-1.51) |
| Digestive | 87 | | 1.76#(1.41-2.17) | 14 | | 2.52#(1.38-4.23) | 42 | | 1.81#(1.3-2.44) | 28 | | 1.71#(1.14-2.48) | 3 | | 0.71(0.15-2.06) |
| Nephritis\ Nephrotic Syndrome\Nephrosis | 313 | | 3.02#(2.7-3.38) | 38 | | 3.50#(2.48-4.8) | 122 | | 2.60#(2.16-3.11) | 116 | | 3.24#(2.67-3.88) | 37 | | 3.69#(2.6-5.09) |
| Pregnancy\Childbirth\Puerperium | 24 | | 4.34#(2.78-6.45) | 4 | | 6.09#(1.66-15.6) | 10 | | 3.77#(1.81-6.92) | 8 | | 4.50#(1.94-8.86) | 2 | | 4.51(0.55-16.29) |
| Symptoms, Signs and Ill-Defined Conditions | 83 | | 1.21(0.96-1.5) | 13 | | 1.86(0.99-3.18) | 40 | | 1.3(0.93-1.76) | 26 | | 1.06(0.69-1.55) | 4 | | 0.63(0.17-1.62) |
| Accidents\Adverse Effects\Homicide\Legal intervention | 172 | | 1.22#(1.04-1.41) | 27 | | 1.86#(1.23-2.71) | 70 | | 1.1(0.86-1.39) | 59 | | 1.2(0.91-1.55) | 16 | | 1.14(0.65-1.85) |
| Suicide\Self-Inflicted Injury | 24 | | 1.5(0.96-2.24) | 2 | | 1.02(0.12-3.68) | 9 | | 1.15(0.52-2.17) | 10 | | 2.01(0.96-3.69) | 3 | | 2.59(0.53-7.56) |
| Other Cause of Death | 1011 | | 1.08#(1.02-1.15) | 149 | | 1.70#(1.44-2) | 391 | | 0.97(0.87-1.07) | 355 | | 1.04(0.94-1.16) | 116 | | 1.15(0.95-1.38) |

Supplementary Table 3. Standardized-mortality ratios (SMRs) for each cause of death following RCC diagnosis in patients aged ＜ 65 years.

|  | | | Timing of Death After Diagnosis | | | | | | | | | | | | |  |
| --- | --- | --- | --- | --- | --- | --- | --- | --- | --- | --- | --- | --- | --- | --- | --- | --- |
|  | | | All Years | | | <1 y | 1 to <5 y | | | 5 to <10 y | | | ≥10 y | | |  |
| All Causes of Death | 17,250 | 4.47#(4.41-4.54) | | 5,263 | 15.26#(14.85-15.68) | | | 7,468 | 4.62#(4.52-4.73) | | 3,538 | 2.50#(2.42-2.59) | | 981 | 2.04#(1.91-2.17) | |
| All Malignant Cancers | 12,410 | 10.33#(10.15-10.51) | | 4,631 | 43.68#(42.43-44.96) | | | 5,414 | 10.74#(10.45-11.03) | | 1,895 | 4.26#(4.07-4.45) | | 470 | 3.22#(2.94-3.53) | |
| Oral Cavity/Pharynx/eye/endocrine | 37 | 1.13(0.79-1.56) | | 7 | 2.3(0.92-4.73) | | | 14 | 0.99(0.54-1.66) | | 11 | 0.93(0.46-1.66) | | 5 | 1.36(0.44-3.17) | |
| Digestive System | 431 | 1.25#(1.13-1.37) | | 50 | 1.63#(1.21-2.15) | | | 152 | 1.04(0.88-1.22) | | 170 | 1.33#(1.14-1.55) | | 59 | 1.43#(1.09-1.85) | |
| Respiratory System | 459 | 1.29#(1.17-1.41) | | 64 | 2.03#(1.56-2.59) | | | 192 | 1.28#(1.11-1.48) | | 151 | 1.14(0.97-1.34) | | 52 | 1.22(0.91-1.6) | |
| Bones and Joints/soft tissue/skin | 54 | 1.44#(1.08-1.87) | | 18 | 5.01#(2.97-7.93) | | | 19 | 1.17(0.7-1.83) | | 11 | 0.81(0.41-1.46) | | 6 | 1.41(0.52-3.06) | |
| Breast | 30 | 0.49#(0.33-0.7) | | 1 | 0.17#(0-0.92) | | | 7 | 0.26#(0.1-0.54) | | 14 | 0.65(0.36-1.09) | | 8 | 1.24(0.54-2.45) | |
| Genital System | 89 | 0.97(0.78-1.19) | | 17 | 2.38#(1.39-3.81) | | | 38 | 1.04(0.74-1.43) | | 25 | 0.7(0.45-1.04) | | 9 | 0.7(0.32-1.33) | |
| Kidney and Renal Pelvis | 10,625 | 328.22#(322.01-334.52) | | 4,256 | 1,447.89#(1404.72-1492.06) | | | 4,741 | 344.17#(334.44-354.11) | | 1,358 | 114.40#(108.4-120.65) | | 270 | 71.31#(63.06-80.35) | |
| Other Urinary Organs | 61 | 2.43#(1.86-3.12) | | 20 | 10.59#(6.47-16.35) | | | 26 | 2.67#(1.75-3.92) | | 14 | 1.43(0.78-2.39) | | 1 | 0.27(0.01-1.5) | |
| Brain/Other Nervous System | 63 | 1.66#(1.28-2.13) | | 7 | 1.96(0.79-4.03) | | | 27 | 1.64#(1.08-2.38) | | 23 | 1.68#(1.07-2.53) | | 6 | 1.42(0.52-3.1) | |
| lymphandblood | 138 | 1.45#(1.22-1.72) | | 26 | 3.23#(2.11-4.73) | | | 51 | 1.32(0.98-1.74) | | 39 | 1.09(0.78-1.49) | | 22 | 1.74#(1.09-2.64) | |
| Miscellaneous Malignant Cancer | 423 | 4.97#(4.5-5.46) | | 165 | 21.79#(18.59-25.38) | | | 147 | 4.12#(3.48-4.84) | | 79 | 2.51#(1.99-3.13) | | 32 | 3.05#(2.08-4.3) | |
| In situ, benign or unknown behavior neoplasm | 54 | 2.86#(2.15-3.73) | | 15 | 10.00#(5.6-16.49) | | | 24 | 3.25#(2.08-4.84) | | 12 | 1.65(0.86-2.89) | | 3 | 1.08(0.22-3.16) | |
| Noncancer | 4,786 | 1.82#(1.76-1.87) | | 617 | 2.60#(2.4-2.81) | | | 2,030 | 1.84#(1.76-1.92) | | 1,631 | 1.70#(1.61-1.78) | | 508 | 1.52#(1.39-1.66) | |
| infections | 282 | 2.22#(1.97-2.5) | | 44 | 3.34#(2.43-4.48) | | | 123 | 2.19#(1.82-2.61) | | 91 | 2.08#(1.67-2.55) | | 24 | 1.78#(1.14-2.64) | |
| Diabetes Mellitus | 380 | 2.54#(2.29-2.81) | | 44 | 3.41#(2.48-4.58) | | | 145 | 2.33#(1.97-2.74) | | 144 | 2.59#(2.18-3.05) | | 47 | 2.50#(1.84-3.33) | |
| Alzheimers (ICD-9 and 10 only) | 20 | 0.96(0.59-1.48) | | 0 | 0(0-5.14) | | | 6 | 1.19(0.44-2.58) | | 9 | 1.01(0.46-1.91) | | 5 | 0.82(0.27-1.92) | |
| Diseases of Heart | 1,466 | 1.68#(1.6-1.77) | | 172 | 2.20#(1.88-2.55) | | | 636 | 1.74#(1.61-1.89) | | 507 | 1.59#(1.46-1.74) | | 151 | 1.38#(1.17-1.62) | |
| Hypertension without Heart Disease | 129 | 3.48#(2.9-4.13) | | 21 | 7.08#(4.38-10.82) | | | 52 | 3.48#(2.6-4.56) | | 41 | 2.91#(2.09-3.95) | | 15 | 2.93#(1.64-4.84) | |
| Cerebrovascular Diseases | 240 | 1.71#(1.5-1.95) | | 28 | 2.40#(1.59-3.47) | | | 104 | 1.86#(1.52-2.25) | | 78 | 1.49#(1.17-1.85) | | 30 | 1.52#(1.02-2.17) | |
| Diseases of Arteries | 78 | 2.34#(1.85-2.91) | | 11 | 3.87#(1.93-6.92) | | | 34 | 2.48#(1.72-3.47) | | 25 | 2.01#(1.3-2.96) | | 8 | 1.82(0.79-3.59) | |
| Pneumonia and Influenza | 372 | 1.46#(1.32-1.62) | | 43 | 2.39#(1.73-3.22) | | | 138 | 1.44#(1.21-1.7) | | 152 | 1.50#(1.27-1.76) | | 39 | 0.99(0.7-1.35) | |
| Digestive | 193 | 1.60#(1.39-1.85) | | 26 | 2.06#(1.34-3.01) | | | 97 | 1.76#(1.43-2.14) | | 59 | 1.44#(1.09-1.85) | | 11 | 0.97(0.48-1.73) | |
| Nephritis\ Nephrotic Syndrome\Nephrosis | 311 | 4.97#(4.43-5.55) | | 34 | 6.82#(4.73-9.53) | | | 133 | 5.36#(4.49-6.35) | | 103 | 4.31#(3.52-5.22) | | 41 | 4.63#(3.32-6.28) | |
| Pregnancy\Childbirth\Puerperium | 34 | 3.76#(2.6-5.26) | | 5 | 4.88#(1.58-11.39) | | | 14 | 3.27#(1.79-5.49) | | 12 | 4.06#(2.1-7.09) | | 3 | 3.85(0.79-11.25) | |
| Symptoms, Signs and Ill-Defined Conditions | 81 | 2.41#(1.91-3) | | 17 | 4.98#(2.9-7.98) | | | 37 | 2.52#(1.77-3.47) | | 23 | 1.96#(1.24-2.94) | | 4 | 1.07(0.29-2.73) | |
| Accidents\Adverse Effects\Homicide\Legal intervention | 286 | 1.24#(1.1-1.39) | | 33 | 1.25(0.86-1.76) | | | 137 | 1.26#(1.05-1.48) | | 85 | 1.14(0.91-1.4) | | 31 | 1.49#(1.01-2.12) | |
| Suicide\Self-Inflicted Injury | 84 | 1(0.8-1.24) | | 11 | 1.11(0.55-1.98) | | | 43 | 1.07(0.77-1.44) | | 25 | 0.94(0.61-1.39) | | 5 | 0.74(0.24-1.72) | |
| Other Cause of Death | 830 | 1.79#(1.67-1.92) | | 128 | 3.33#(2.78-3.96) | | | 331 | 1.78#(1.59-1.98) | | 277 | 1.60#(1.41-1.8) | | 94 | 1.46#(1.18-1.78) | |

Supplementary Table 4. Standardized-mortality ratios (SMRs) for each cause of death following RCC diagnosis in patients aged ≥ 65 years.

|  | | Timing of Death After Diagnosis | | | | | | | | | | | | | | |  |
| --- | --- | --- | --- | --- | --- | --- | --- | --- | --- | --- | --- | --- | --- | --- | --- | --- | --- |
|  | All Y ears | | | <1 y | | | 1 to <5 y | | | 5 to <10 y | | | ≥10 y | | |  |  |
| All Causes of Death | 22,380 | | 2.03#（2-2.05） | | 6,479 | 5.21#（5.09-5.34） | | 9,363 | 1.81#（1.78-1.85） | | 5,232 | 1.42#（1.38-1.46） | | 1,306 | 1.37#（1.29-1.44） | | |
| All Malignant Cancers | 12,686 | | 5.24#（5.15-5.34） | | 5,089 | 16.50#（16.05-16.96） | | 5,373 | 4.49#（4.37-4.61） | | 1,821 | 2.44#（2.33-2.55） | | 403 | 2.42#（2.19-2.67） | | |
| Oral Cavity/Pharynx/eye/endocrine | 34 | | 0.79（0.54-1.1） | | 9 | 1.63（0.75-3.09） | | 13 | 0.6（0.32-1.03） | | 8 | 0.6（0.26-1.19） | | 4 | 1.34（0.37-3.44） | | |
| Digestive System | 631 | | 1.06（0.98-1.15） | | 107 | 1.41#（1.16-1.7） | | 282 | 0.95（0.85-1.07） | | 186 | 1.02（0.87-1.17） | | 56 | 1.38#（1.04-1.79） | | |
| Respiratory System | 746 | | 1.09#（1.01-1.17） | | 140 | 1.49#（1.25-1.76） | | 328 | 0.94（0.84-1.05） | | 226 | 1.13（0.98-1.28） | | 52 | 1.3（0.97-1.7） | | |
| Bones and Joints/soft tissue/skin | 72 | | 1.01（0.79-1.27） | | 19 | 2.23#（1.34-3.47） | | 27 | 0.78（0.52-1.14） | | 21 | 0.92（0.57-1.41） | | 5 | 0.92（0.3-2.15） | | |
| Breast | 41 | | 0.41#（0.3-0.56） | | 5 | 0.40#（0.13-0.93） | | 11 | 0.23#（0.11-0.4） | | 18 | 0.58#（0.35-0.92） | | 7 | 1（0.4-2.06） | | |
| Genital System | 162 | | 0.58#（0.49-0.68） | | 25 | 0.76（0.49-1.12） | | 68 | 0.51#（0.39-0.64） | | 49 | 0.54#（0.4-0.72） | | 20 | 0.9（0.55-1.39） | | |
| Kidney and Renal Pelvis | 9,870 | | 172.00#（168.62-175.43） | | 4,442 | 607.68#（589.94-625.82） | | 4,157 | 146.16#（141.75-150.67） | | 1,063 | 60.12#（56.56-63.84） | | 208 | 52.65#（45.73-60.31） | | |
| Other Urinary Organs | 169 | | 1.80#（1.54-2.1） | | 48 | 4.61#（3.4-6.12） | | 86 | 1.96#（1.57-2.42） | | 29 | 0.92（0.62-1.32） | | 6 | 0.74（0.27-1.61） | | |
| Brain/Other Nervous System | 78 | | 1.75#（1.38-2.19） | | 11 | 1.87（0.93-3.34） | | 40 | 1.77#（1.26-2.41） | | 23 | 1.74#（1.1-2.6） | | 4 | 1.46（0.4-3.73） | | |
| lymphandblood | 302 | | 1.15#（1.02-1.28） | | 61 | 1.92#（1.47-2.46） | | 134 | 1.05（0.88-1.24） | | 91 | 1.08（0.87-1.32） | | 16 | 0.82（0.47-1.32） | | |
| Miscellaneous Malignant Cancer | 581 | | 3.09#（2.84-3.35） | | 222 | 9.51#（8.3-10.84） | | 227 | 2.47#（2.16-2.82） | | 107 | 1.81#（1.48-2.19） | | 25 | 1.82#（1.18-2.69） | | |
| In situ, benign or unknown behavior neoplasm | 121 | | 1.58#（1.31-1.89） | | 23 | 2.73#（1.73-4.1） | | 50 | 1.40#（1.04-1.85） | | 40 | 1.54#（1.1-2.1） | | 8 | 1.21（0.52-2.39） | | |
| Noncancer | 9,573 | | 1.12#（1.1-1.14） | | 1,367 | 1.48#（1.4-1.56） | | 3,940 | 1（0.97-1.03） | | 3,371 | 1.16#（1.12-1.2） | | 895 | 1.14#（1.07-1.22） | | |
| infections | 324 | | 1.35#（1.2-1.5） | | 73 | 2.59#（2.03-3.26） | | 127 | 1.1（0.92-1.31） | | 98 | 1.25#（1.01-1.52） | | 26 | 1.35（0.88-1.98） | | |
| Diabetes Mellitus | 528 | | 1.65#（1.51-1.8） | | 82 | 2.11#（1.68-2.62） | | 247 | 1.59#（1.4-1.8） | | 165 | 1.63#（1.39-1.89） | | 34 | 1.42（0.99-1.99） | | |
| Alzheimers (ICD-9 and 10 only) | 425 | | 0.83#（0.75-0.91） | | 28 | 0.65#（0.43-0.93） | | 146 | 0.69#（0.58-0.81） | | 188 | 0.97（0.83-1.12） | | 63 | 0.99（0.76-1.27） | | |
| Diseases of Heart | 3,288 | | 1.13#（1.1-1.17） | | 505 | 1.55#（1.42-1.69） | | 1,354 | 1.01（0.95-1.06） | | 1,127 | 1.16#（1.1-1.23） | | 302 | 1.17#（1.04-1.31） | | |
| Hypertension without Heart Disease | 254 | | 1.85#（1.63-2.09） | | 21 | 1.52（0.94-2.32） | | 95 | 1.54#（1.25-1.89） | | 106 | 2.19#（1.79-2.65） | | 32 | 2.35#（1.61-3.32） | | |
| Cerebrovascular Diseases | 703 | | 1.07（1-1.16） | | 98 | 1.38#（1.12-1.68） | | 318 | 1.06（0.95-1.19） | | 223 | 1（0.87-1.14） | | 64 | 1.04（0.8-1.33） | | |
| Diseases of Arteries | 116 | | 0.92（0.76-1.1） | | 22 | 1.42（0.89-2.15） | | 44 | 0.73#（0.53-0.98） | | 43 | 1.07（0.77-1.44） | | 7 | 0.71（0.28-1.46） | | |
| Pneumonia and Influenza | 964 | | 0.96（0.9-1.02） | | 121 | 1.06（0.88-1.27） | | 412 | 0.87#（0.79-0.96） | | 355 | 1.06（0.95-1.18） | | 76 | 0.91（0.72-1.14） | | |
| Digestive | 101 | | 1.22（0.99-1.48） | | 14 | 1.23（0.67-2.07） | | 45 | 1.05（0.77-1.41） | | 34 | 1.41（0.98-1.97） | | 8 | 1.61（0.69-3.17） | | |
| Nephritis\ Nephrotic Syndrome\Nephrosis | 548 | | 2.30#（2.11-2.5） | | 72 | 2.72#（2.13-3.42） | | 215 | 1.93#（1.68-2.21） | | 203 | 2.54#（2.2-2.92） | | 58 | 2.83#（2.15-3.66） | | |
| Pregnancy\Childbirth\Puerperium | 8 | | 1.31（0.56-2.58） | | 1 | 1.26（0.03-7.01） | | 5 | 1.65（0.54-3.85） | | 1 | 0.54（0.01-3） | | 1 | 2.3（0.06-12.8） | | |
| Symptoms, Signs and Ill-Defined Conditions | 137 | | 1.08（0.91-1.28） | | 25 | 1.86#（1.21-2.75） | | 60 | 1.03（0.79-1.33） | | 46 | 1.04（0.76-1.39） | | 6 | 0.56（0.2-1.21） | | |
| Accidents\Adverse Effects\Homicide\Legal intervention | 296 | | 1.06（0.94-1.18） | | 41 | 1.36（0.97-1.84） | | 134 | 1.04（0.87-1.23） | | 98 | 1.03（0.83-1.25） | | 23 | 0.89（0.56-1.33） | | |
| Suicide\Self-Inflicted Injury | 56 | | 1.2（0.91-1.56） | | 10 | 1.63（0.78-3） | | 26 | 1.1（0.72-1.62） | | 16 | 1.16（0.66-1.88） | | 4 | 1.34（0.37-3.44） | | |
| Other Cause of Death | 1,825 | | 0.97（0.93-1.02） | | 254 | 1.36#（1.2-1.54） | | 712 | 0.85#（0.79-0.91） | | 668 | 1（0.93-1.08） | | 191 | 1.03（0.89-1.19） | | |

Supplementary Table 5. Standardized-mortality ratios (SMRs) for each cause of death following RCC diagnosis in white patients.

|  | | Timing of Death After Diagnosis | | | | | | | | | | | | | | |
| --- | --- | --- | --- | --- | --- | --- | --- | --- | --- | --- | --- | --- | --- | --- | --- | --- |
|  | | All Y ears | | | <1 y | | | 1 to <5 y | | | 5 to <10 y | | | ≥10 y | | |
| All Causes of Death | 32,649 | | 2.57#（2.54-2.59） | 9,538 | | 7.10#（6.96-7.24） | 13,873 | | 2.41#（2.37-2.45） | 7,340 | | 1.68#（1.64-1.72） | 1,898 | | 1.53#（1.46-1.6） |  |
| All Malignant Cancers | 20,913 | | 6.81#（6.72-6.9） | 7,961 | | 22.73#（22.23-23.23） | 9,047 | | 6.28#（6.15-6.41） | 3,173 | | 3.13#（3.02-3.24） | 732 | | 2.73#（2.54-2.94） |  |
| Oral Cavity/Pharynx/eye/endocrine | 58 | | 0.91（0.69-1.17） | 11 | | 1.55（0.77-2.77） | 24 | | 0.8（0.52-1.2） | 14 | | 0.66（0.36-1.11） | 9 | | 1.57（0.72-2.99） |  |
| Digestive System | 870 | | 1.13#（1.05-1.2） | 129 | | 1.48#（1.24-1.76） | 359 | | 0.99（0.89-1.1） | 290 | | 1.13#（1.01-1.27） | 92 | | 1.35#（1.09-1.66） |  |
| Respiratory System | 1,017 | | 1.14#（1.07-1.21） | 168 | | 1.56#（1.34-1.82） | 438 | | 1.03（0.93-1.13） | 329 | | 1.15#（1.03-1.29） | 82 | | 1.15（0.91-1.43） |  |
| Bones and Joints/soft tissue/skin | 105 | | 1.03（0.84-1.25） | 29 | | 2.56#（1.72-3.68） | 41 | | 0.87（0.62-1.17） | 26 | | 0.76（0.5-1.12） | 9 | | 0.99（0.45-1.87） |  |
| Breast | 58 | | 0.44#（0.33-0.57） | 6 | | 0.39#（0.14-0.86） | 17 | | 0.27#（0.16-0.44） | 22 | | 0.51#（0.32-0.77） | 13 | | 1.15（0.61-1.97） |  |
| Genital System | 205 | | 0.68#（0.59-0.78） | 34 | | 1.04（0.72-1.46） | 84 | | 0.61#（0.49-0.75） | 65 | | 0.63#（0.49-0.81） | 22 | | 0.76（0.48-1.15） |  |
| Kidney and Renal Pelvis | 17,051 | | 216.98#（213.74-220.27） | 7,121 | | 796.24#（777.85-814.95） | 7,454 | | 202.09#（197.53-206.73） | 2,073 | | 79.99#（76.58-83.51） | 403 | | 58.94#（53.33-64.99） |  |
| Other Urinary Organs | 202 | | 1.87#（1.62-2.15） | 57 | | 5.12#（3.88-6.63） | 99 | | 2.04#（1.66-2.49） | 40 | | 1.07（0.76-1.45） | 6 | | 0.56（0.2-1.21） |  |
| Brain/Other Nervous System | 127 | | 1.68#（1.4-2） | 15 | | 1.73（0.97-2.85） | 60 | | 1.68#（1.28-2.16） | 42 | | 1.71#（1.23-2.31） | 10 | | 1.56（0.75-2.88） |  |
| lymphandblood | 384 | | 1.23#（1.11-1.36） | 72 | | 2.08#（1.63-2.62） | 161 | | 1.11（0.95-1.3） | 118 | | 1.13（0.93-1.35） | 33 | | 1.16（0.8-1.63） |  |
| Miscellaneous Malignant Cancer | 836 | | 3.56#（3.32-3.81） | 319 | | 12.08#（10.79-13.48） | 310 | | 2.84#（2.53-3.17） | 154 | | 1.97#（1.67-2.31） | 53 | | 2.51#（1.88-3.28） |  |
| In situ, benign or unknown behavior neoplasm | 147 | | 1.74#（1.47-2.04） | 30 | | 3.42#（2.31-4.89） | 62 | | 1.63#（1.25-2.09） | 46 | | 1.56#（1.14-2.08） | 9 | | 1.07（0.49-2.04） |  |
| Noncancer | 11,589 | | 1.21#（1.19-1.23） | 1,547 | | 1.57#（1.49-1.65） | 4,764 | | 1.11#（1.08-1.14） | 4,121 | | 1.24#（1.2-1.28） | 1,157 | | 1.20#（1.13-1.27） |  |
| infections | 453 | | 1.56#（1.42-1.71） | 79 | | 2.49#（1.97-3.1） | 182 | | 1.36#（1.17-1.57） | 153 | | 1.57#（1.33-1.83） | 39 | | 1.46#（1.04-1.99） |  |
| Diabetes Mellitus | 688 | | 1.89#（1.75-2.03） | 90 | | 2.25#（1.81-2.76） | 299 | | 1.77#（1.58-1.99） | 233 | | 1.90#（1.66-2.16） | 66 | | 1.96#（1.51-2.49） |  |
| Alzheimers (ICD-9 and 10 only) | 397 | | 0.82#（0.74-0.91） | 24 | | 0.60#（0.39-0.9） | 139 | | 0.71#（0.59-0.83） | 178 | | 0.97（0.83-1.12） | 56 | | 0.89（0.68-1.16） |  |
| Diseases of Heart | 3,834 | | 1.19#（1.16-1.23） | 535 | | 1.57#（1.44-1.71） | 1,576 | | 1.09#（1.03-1.14） | 1,345 | | 1.22#（1.16-1.29） | 378 | | 1.19#（1.07-1.32） |  |
| Hypertension without Heart Disease | 276 | | 2.06#（1.83-2.32） | 27 | | 2.15#（1.42-3.13） | 101 | | 1.74#（1.42-2.12） | 112 | | 2.31#（1.9-2.78） | 36 | | 2.44#（1.71-3.38） |  |
| Cerebrovascular Diseases | 765 | | 1.17#（1.09-1.25） | 101 | | 1.50#（1.22-1.82） | 336 | | 1.15#（1.03-1.29） | 245 | | 1.07（0.94-1.21） | 83 | | 1.22（0.97-1.51） |  |
| Diseases of Arteries | 161 | | 1.17（1-1.37） | 23 | | 1.45（0.92-2.18） | 65 | | 1.02（0.79-1.3） | 61 | | 1.34#（1.02-1.72） | 12 | | 0.97（0.5-1.69） |  |
| Pneumonia and Influenza | 1,161 | | 1.03（0.97-1.09） | 140 | | 1.19（1-1.4） | 479 | | 0.94（0.86-1.03） | 444 | | 1.14#（1.04-1.25） | 98 | | 0.89（0.72-1.08） |  |
| Digestive | 257 | | 1.44#（1.27-1.63） | 36 | | 1.73#（1.21-2.39） | 129 | | 1.51#（1.26-1.8） | 74 | | 1.29#（1.02-1.63） | 18 | | 1.24（0.73-1.96） |  |
| Nephritis\ Nephrotic Syndrome\Nephrosis | 608 | | 2.58#（2.38-2.79） | 80 | | 3.28#（2.6-4.09） | 242 | | 2.28#（2-2.59） | 213 | | 2.60#（2.26-2.97） | 73 | | 3.11#（2.44-3.91） |  |
| Pregnancy\Childbirth\Puerperium | 31 | | 2.35#（1.6-3.33） | 5 | | 3.17#（1.03-7.4） | 13 | | 2.05#（1.09-3.5） | 10 | | 2.38#（1.14-4.37） | 3 | | 2.8（0.58-8.17） |  |
| Symptoms, Signs and Ill-Defined Conditions | 169 | | 1.22#（1.04-1.42） | 26 | | 1.80#（1.18-2.64） | 82 | | 1.31#（1.04-1.62） | 54 | | 1.1（0.83-1.44） | 7 | | 0.55（0.22-1.13） |  |
| Accidents\Adverse Effects\Homicide\Legal intervention | 479 | | 1.09（1-1.2） | 56 | | 1.17（0.88-1.52） | 222 | | 1.1（0.96-1.25） | 157 | | 1.07（0.91-1.25） | 44 | | 1.08（0.78-1.45） |  |
| Suicide\Self-Inflicted Injury | 131 | | 1.07（0.9-1.28） | 19 | | 1.27（0.76-1.98） | 65 | | 1.09（0.84-1.39） | 39 | | 1.03（0.73-1.4） | 8 | | 0.87（0.37-1.71） |  |
| Other Cause of Death | 2,179 | | 1.07#（1.02-1.12） | 306 | | 1.58#（1.41-1.76） | 834 | | 0.94（0.88-1） | 803 | | 1.09#（1.02-1.17） | 236 | | 1.07（0.94-1.22） |  |

Supplementary Table 6. Standardized-mortality ratios (SMRs) for each cause of death following RCC diagnosis in black patients.

|  | | Timing of Death After Diagnosis | | | | | | | | | | | | | | |
| --- | --- | --- | --- | --- | --- | --- | --- | --- | --- | --- | --- | --- | --- | --- | --- | --- |
|  | | All Y ears | | | <1 y | | | 1 to <5 y | | | 5 to <10 y | | | ≥10 y | | |
| All Causes of Death | 4,724 | | 2.85#（2.77-2.93） | 1,490 | | 7.96#（7.56-8.37） | 1,985 | | 2.55#（2.44-2.66） | 988 | | 1.80#（1.69-1.92） | 261 | | 1.79#（1.58-2.02） |  |
| All Malignant Cancers | 2,607 | | 6.24#（6-6.48） | 1,155 | | 23.53#（22.19-24.93） | 1,041 | | 5.21#（4.89-5.53） | 328 | | 2.42#（2.17-2.7） | 83 | | 2.47#（1.97-3.06） |  |
| Oral Cavity/Pharynx/eye/endocrine | 7 | | 0.81（0.33-1.67） | 2 | | 1.89（0.23-6.84） | 3 | | 0.71（0.15-2.09） | 2 | | 0.73（0.09-2.65） | 0 | | 0（0-5.67） |  |
| Digestive System | 133 | | 1.09（0.92-1.3） | 23 | | 1.62#（1.03-2.44） | 48 | | 0.83（0.61-1.09） | 47 | | 1.19（0.87-1.58） | 15 | | 1.52（0.85-2.51） |  |
| Respiratory System | 133 | | 1.15（0.96-1.36） | 29 | | 2.04#（1.37-2.93） | 59 | | 1.05（0.8-1.35） | 33 | | 0.9（0.62-1.26） | 12 | | 1.38（0.71-2.41） |  |
| Bones and Joints/soft tissue/skin | 14 | | 2.82#（1.54-4.73） | 6 | | 10.31#（3.78-22.43） | 4 | | 1.68（0.46-4.3） | 3 | | 1.87（0.39-5.47） | 1 | | 2.52（0.06-14.05） |  |
| Breast | 10 | | 0.42#（0.2-0.78） | 0 | | 0（0-1.28） | 1 | | 0.09#（0-0.48） | 7 | | 0.94（0.38-1.94） | 2 | | 1.13（0.14-4.07） |  |
| Genital System | 38 | | 0.65#（0.46-0.89） | 8 | | 1.24（0.54-2.45） | 17 | | 0.62#（0.36-1） | 8 | | 0.41#（0.18-0.81） | 5 | | 0.96（0.31-2.24） |  |
| Kidney and Renal Pelvis | 2,082 | | 252.55#（241.82-263.64） | 1,020 | | 1,065.34#（1000.96-1132.78） | 829 | | 211.19#（197.05-226.06） | 190 | | 70.76#（61.05-81.56） | 43 | | 63.65#（46.07-85.74） |  |
| Other Urinary Organs | 19 | | 2.32#（1.4-3.63） | 5 | | 5.77#（1.87-13.47） | 12 | | 3.20#（1.65-5.59） | 1 | | 0.36（0.01-1.99） | 1 | | 1.31（0.03-7.28） |  |
| Brain/Other Nervous System | 8 | | 1.64（0.71-3.24） | 3 | | 5.33#（1.1-15.57） | 3 | | 1.29（0.27-3.76） | 2 | | 1.26（0.15-4.57） | 0 | | 0（0-9.49） |  |
| lymphandblood | 37 | | 1.09（0.77-1.5） | 9 | | 2.32#（1.06-4.4） | 14 | | 0.87（0.48-1.46） | 11 | | 0.98（0.49-1.75） | 3 | | 1.05（0.22-3.06） |  |
| Miscellaneous Malignant Cancer | 126 | | 4.27#（3.56-5.09） | 50 | | 14.29#（10.61-18.84） | 51 | | 3.62#（2.69-4.75） | 24 | | 2.53#（1.62-3.76） | 1 | | 0.42（0.01-2.34） |  |
| In situ, benign or unknown behavior neoplasm | 20 | | 2.69#（1.64-4.15） | 6 | | 7.48#（2.74-16.27） | 9 | | 2.63#（1.2-4.98） | 4 | | 1.59（0.43-4.07） | 1 | | 1.44（0.04-8.03） |  |
| Noncancer | 2,097 | | 1.70#（1.63-1.77） | 329 | | 2.40#（2.14-2.67） | 935 | | 1.63#（1.52-1.73） | 656 | | 1.60#（1.48-1.73） | 177 | | 1.59#（1.37-1.84） |  |
| infections | 129 | | 2.00#（1.67-2.38） | 29 | | 3.59#（2.41-5.16） | 60 | | 1.91#（1.46-2.46） | 32 | | 1.59#（1.09-2.25） | 8 | | 1.62（0.7-3.2） |  |
| Diabetes Mellitus | 155 | | 1.94#（1.65-2.28） | 25 | | 2.75#（1.78-4.06） | 64 | | 1.70#（1.31-2.17） | 56 | | 2.14#（1.62-2.78） | 10 | | 1.48（0.71-2.72） |  |
| Alzheimers (ICD-9 and 10 only) | 36 | | 0.97（0.68-1.35） | 4 | | 1.32（0.36-3.38） | 6 | | 0.39#（0.14-0.86） | 15 | | 1.08（0.61-1.79） | 11 | | 2.23#（1.11-3.98） |  |
| Diseases of Heart | 718 | | 1.66#（1.54-1.78） | 114 | | 2.30#（1.9-2.77） | 329 | | 1.62#（1.45-1.8） | 222 | | 1.56#（1.36-1.78） | 53 | | 1.40#（1.05-1.83） |  |
| Hypertension without Heart Disease | 85 | | 2.68#（2.14-3.32） | 10 | | 2.93#（1.41-5.39） | 38 | | 2.60#（1.84-3.57） | 28 | | 2.62#（1.74-3.78） | 9 | | 3.03#（1.38-5.75） |  |
| Cerebrovascular Diseases | 124 | | 1.22#（1.02-1.46） | 15 | | 1.33（0.75-2.2） | 63 | | 1.34#（1.03-1.71） | 38 | | 1.12（0.79-1.54） | 8 | | 0.85（0.37-1.67） |  |
| Diseases of Arteries | 28 | | 1.68#（1.12-2.43） | 8 | | 4.12#（1.78-8.11） | 12 | | 1.52（0.79-2.66） | 5 | | 0.92（0.3-2.16） | 3 | | 2.08（0.43-6.08） |  |
| Pneumonia and Influenza | 127 | | 1.36#（1.13-1.62） | 21 | | 2.10#（1.3-3.21） | 51 | | 1.18（0.88-1.56） | 44 | | 1.39#（1.01-1.86） | 11 | | 1.27（0.63-2.27） |  |
| Digestive | 21 | | 1.18（0.73-1.8） | 3 | | 1.35（0.28-3.96） | 8 | | 0.91（0.39-1.8） | 9 | | 1.63（0.74-3.09） | 1 | | 0.78（0.02-4.32） |  |
| Nephritis\ Nephrotic Syndrome\Nephrosis | 212 | | 4.01#（3.48-4.58） | 22 | | 3.74#（2.34-5.66） | 88 | | 3.56#（2.86-4.39） | 78 | | 4.43#（3.5-5.53） | 24 | | 5.08#（3.25-7.55） |  |
| Pregnancy\Childbirth\Puerperium | 8 | | 5.28#（2.28-10.4） | 1 | | 5.27（0.13-29.34） | 4 | | 5.34#（1.46-13.68） | 2 | | 4.27（0.52-15.43） | 1 | | 9.19（0.23-51.23） |  |
| Symptoms, Signs and Ill-Defined Conditions | 43 | | 2.53#（1.83-3.41） | 15 | | 7.51#（4.2-12.38） | 13 | | 1.6（0.85-2.74） | 12 | | 2.17#（1.12-3.79） | 3 | | 2.19（0.45-6.41） |  |
| Accidents\Adverse Effects\Homicide\Legal intervention | 71 | | 1.29#（1.01-1.63） | 10 | | 1.49（0.71-2.74） | 37 | | 1.38（0.97-1.9） | 18 | | 1.05（0.62-1.66） | 6 | | 1.4（0.51-3.05） |  |
| Suicide\Self-Inflicted Injury | 4 | | 0.88（0.24-2.26） | 2 | | 3.27（0.4-11.82） | 2 | | 0.87（0.11-3.15） | 0 | | 0（0-2.78） | 0 | | 0（0-12.71） |  |
| Other Cause of Death | 336 | | 1.48#（1.33-1.65） | 50 | | 2.13#（1.58-2.81） | 160 | | 1.55#（1.32-1.81） | 97 | | 1.24#（1-1.51） | 29 | | 1.31（0.88-1.88） |  |

Supplementary Table 7. Standardized-mortality ratios (SMRs) for each cause of death following RCC diagnosis in patients of other races.

|  | | Timing of Death After Diagnosis | | | | | | | | | | | | | | |
| --- | --- | --- | --- | --- | --- | --- | --- | --- | --- | --- | --- | --- | --- | --- | --- | --- |
|  | | All Y ears | | | <1 y | | | 1 to <5 y | | | 5 to <10 y | | | ≥10 y | | |
| All Causes of Death | 2,257 | | 4.29#（4.12-4.47） | 714 | | 12.68#（11.77-13.65） | 973 | | 4.05#（3.8-4.31） | 442 | | 2.44#（2.21-2.67） | 128 | | 2.66#（2.22-3.17） |  |
| All Malignant Cancers | 1,576 | | 11.99#（11.41-12.6） | 604 | | 39.93#（36.81-43.25） | 699 | | 11.24#（10.42-12.1） | 215 | | 4.95#（4.31-5.65） | 58 | | 5.46#（4.15-7.06） |  |
| Oral Cavity/Pharynx/eye/endocrine | 6 | | 1.71（0.63-3.72） | 3 | | 7.45#（1.54-21.77） | 0 | | 0（0-2.21） | 3 | | 2.6（0.54-7.61） | 0 | | 0（0-12.93） |  |
| Digestive System | 59 | | 1.25（0.95-1.62） | 5 | | 0.92（0.3-2.14） | 27 | | 1.21（0.8-1.76） | 19 | | 1.23（0.74-1.92） | 8 | | 2.13（0.92-4.2） |  |
| Respiratory System | 55 | | 1.62#（1.22-2.1） | 7 | | 1.74（0.7-3.59） | 23 | | 1.41（0.9-2.12） | 15 | | 1.35（0.76-2.23） | 10 | | 3.80#（1.82-6.99） |  |
| Bones and Joints/soft tissue/skin | 7 | | 3.56#（1.43-7.34） | 2 | | 8.86#（1.07-32） | 1 | | 1.07（0.03-5.96） | 3 | | 4.64（0.96-13.55） | 1 | | 6.35（0.16-35.36） |  |
| Breast | 3 | | 0.64（0.13-1.87） | 0 | | 0（0-6.79） | 0 | | 0（0-1.67） | 3 | | 1.95（0.4-5.69） | 0 | | 0（0-9.09） |  |
| Genital System | 8 | | 0.7（0.3-1.39） | 0 | | 0（0-3） | 5 | | 0.96（0.31-2.23） | 1 | | 0.26（0.01-1.43） | 2 | | 2.02（0.24-7.28） |  |
| Kidney and Renal Pelvis | 1,362 | | 464.88#（440.52-490.24） | 557 | | 1,598.45#（1468.44-1736.88） | 615 | | 437.15#（403.28-473.11） | 158 | | 166.32#（141.4-194.37） | 32 | | 142.54#（97.5-201.23） |  |
| Other Urinary Organs | 9 | | 3.13#（1.43-5.93） | 6 | | 20.19#（7.41-43.96） | 1 | | 0.77（0.02-4.28） | 2 | | 1.97（0.24-7.12） | 0 | | 0（0-13.99） |  |
| Brain/Other Nervous System | 6 | | 2.76#（1.01-6） | 0 | | 0（0-15.16） | 4 | | 3.89#（1.06-9.97） | 2 | | 2.77（0.34-10.02） | 0 | | 0（0-20.21） |  |
| lymphandblood | 19 | | 1.58（0.95-2.46） | 6 | | 4.45#（1.63-9.69） | 10 | | 1.77（0.85-3.26） | 1 | | 0.25（0.01-1.38） | 2 | | 1.99（0.24-7.18） |  |
| Miscellaneous Malignant Cancer | 42 | | 4.78#（3.45-6.46） | 18 | | 17.70#（10.49-27.97） | 13 | | 3.13#（1.67-5.35） | 8 | | 2.76#（1.19-5.43） | 3 | | 4.21（0.87-12.3） |  |
| In situ, benign or unknown behavior neoplasm | 8 | | 2.40#（1.04-4.74） | 2 | | 5.63（0.68-20.34） | 3 | | 1.97（0.41-5.75） | 2 | | 1.73（0.21-6.26） | 1 | | 3.38（0.09-18.82） |  |
| Noncancer | 673 | | 1.72#（1.59-1.85） | 108 | | 2.65#（2.17-3.2） | 271 | | 1.54#（1.36-1.73） | 225 | | 1.64#（1.44-1.87） | 69 | | 1.86#（1.45-2.35） |  |
| infections | 24 | | 1.80#（1.15-2.68） | 9 | | 5.87#（2.69-11.15） | 8 | | 1.28（0.55-2.51） | 4 | | 0.9（0.25-2.31） | 3 | | 2.74（0.57-8.02） |  |
| Diabetes Mellitus | 65 | | 2.66#（2.05-3.38） | 11 | | 4.15#（2.07-7.42） | 29 | | 2.57#（1.72-3.68） | 20 | | 2.40#（1.46-3.7） | 5 | | 2.29（0.74-5.35） |  |
| Alzheimers (ICD-9 and 10 only) | 12 | | 0.78（0.4-1.36） | 0 | | 0（0-3.21） | 7 | | 1.16（0.47-2.39） | 4 | | 0.65（0.18-1.67） | 1 | | 0.47（0.01-2.6） |  |
| Diseases of Heart | 202 | | 1.61#（1.39-1.84） | 28 | | 2.06#（1.37-2.98） | 85 | | 1.48#（1.18-1.83） | 67 | | 1.55#（1.2-1.97） | 22 | | 1.92#（1.2-2.9） |  |
| Hypertension without Heart Disease | 22 | | 2.40#（1.5-3.63） | 5 | | 5.94#（1.93-13.86） | 8 | | 2.03（0.88-4） | 7 | | 2.06（0.83-4.25） | 2 | | 1.97（0.24-7.13） |  |
| Cerebrovascular Diseases | 54 | | 1.43#（1.07-1.86） | 10 | | 2.49#（1.2-4.59） | 23 | | 1.35（0.86-2.02） | 18 | | 1.37（0.81-2.16） | 3 | | 0.82（0.17-2.4） |  |
| Diseases of Arteries | 5 | | 0.95（0.31-2.22） | 2 | | 3.29（0.4-11.89） | 1 | | 0.41（0.01-2.27） | 2 | | 1.14（0.14-4.13） | 0 | | 0（0-8.16） |  |
| Pneumonia and Influenza | 48 | | 1.21（0.89-1.61） | 3 | | 0.74（0.15-2.17） | 20 | | 1.13（0.69-1.74） | 19 | | 1.35（0.81-2.11） | 6 | | 1.6（0.59-3.48） |  |
| Digestive | 16 | | 2.08#（1.19-3.38） | 1 | | 1.06（0.03-5.93） | 5 | | 1.33（0.43-3.1） | 10 | | 4.12#（1.97-7.57） | 0 | | 0（0-6.52） |  |
| Nephritis\ Nephrotic Syndrome\Nephrosis | 39 | | 3.30#（2.35-4.52） | 4 | | 3.24（0.88-8.3） | 18 | | 3.37#（2-5.32） | 15 | | 3.64#（2.04-6） | 2 | | 1.81（0.22-6.53） |  |
| Pregnancy\Childbirth\Puerperium | 3 | | 6.80#（1.4-19.88） | 0 | | 0（0-68.45） | 2 | | 9.33#（1.13-33.7） | 1 | | 7.18（0.18-39.99） | 0 | | 0（0-110.13） |  |
| Symptoms, Signs and Ill-Defined Conditions | 6 | | 1.47（0.54-3.2） | 1 | | 2.36（0.06-13.12） | 2 | | 1.08（0.13-3.9） | 3 | | 2.08（0.43-6.08） | 0 | | 0（0-10.1） |  |
| Accidents\Adverse Effects\Homicide\Legal intervention | 32 | | 1.74#（1.19-2.46） | 8 | | 3.85#（1.66-7.59） | 12 | | 1.39（0.72-2.43） | 8 | | 1.31（0.57-2.58） | 4 | | 2.57（0.7-6.59） |  |
| Suicide\Self-Inflicted Injury | 5 | | 1.38（0.45-3.22） | 0 | | 0（0-7.81） | 2 | | 1.1（0.13-3.97） | 2 | | 1.83（0.22-6.61） | 1 | | 4.22（0.11-23.52） |  |
| Other Cause of Death | 140 | | 1.88#（1.58-2.22） | 26 | | 3.61#（2.36-5.3） | 49 | | 1.50#（1.11-1.98） | 45 | | 1.67#（1.22-2.23） | 20 | | 2.66#（1.62-4.1） |  |

Supplementary Table 8. Standardized-mortality ratios (SMRs) for each cause of death following RCC diagnosis in married patients.

|  | Timing of Death After Diagnosis | | | | | | | | | |
| --- | --- | --- | --- | --- | --- | --- | --- | --- | --- | --- |
|  | All Y ears | | <1 y | | 1 to <5 y | | 5 to <10 y | | ≥10 y | |
| All Causes of Death | 22,182 | 2.40#(2.37-2.43) | 6,288 | 6.84#(6.67-7.01) | 9,399 | 2.30#(2.26-2.35) | 5,096 | 1.57#(1.52-1.61) | 1,399 | 1.42#(1.35-1.5) |
| All Malignant Cancers | 14,718 | 6.26#(6.16-6.37) | 5,358 | 21.00#(20.44-21.57) | 6,433 | 5.96#(5.81-6.11) | 2,355 | 2.96#(2.84-3.09) | 572 | 2.60#(2.39-2.82) |
| Oral Cavity/Pharynx/eye/endocrine | 36 | 0.71#(0.5-0.98) | 10 | 1.83(0.88-3.37) | 12 | 0.51#(0.27-0.9) | 9 | 0.53#(0.24-1) | 5 | 1.05(0.34-2.45) |
| Digestive System | 613 | 1(0.93-1.09) | 87 | 1.32#(1.06-1.63) | 241 | 0.86#(0.75-0.97) | 213 | 1.03(0.9-1.18) | 72 | 1.26(0.98-1.58) |
| Respiratory System | 686 | 1(0.92-1.08) | 110 | 1.39#(1.14-1.67) | 281 | 0.87#(0.77-0.98) | 231 | 1.02(0.9-1.17) | 64 | 1.09(0.84-1.39) |
| Bones and Joints/soft tissue/skin | 79 | 1.07(0.85-1.34) | 19 | 2.43#(1.46-3.8) | 29 | 0.86(0.58-1.24) | 22 | 0.87(0.55-1.32) | 9 | 1.27(0.58-2.42) |
| Breast | 32 | 0.41#(0.28-0.57) | 1 | 0.12#(0-0.66) | 8 | 0.22#(0.1-0.44) | 17 | 0.63(0.37-1.01) | 6 | 0.78(0.28-1.69) |
| Genital System | 139 | 0.58#(0.49-0.68) | 18 | 0.74(0.44-1.17) | 54 | 0.50#(0.38-0.66) | 47 | 0.56#(0.41-0.74) | 20 | 0.8(0.49-1.24) |
| Kidney and Renal Pelvis | 12,075 | 200.54#(196.98-204.15) | 4,830 | 735.38#(714.78-756.41) | 5,373 | 193.52#(188.38-198.76) | 1,555 | 76.58#(72.82-80.48) | 317 | 56.87#(50.78-63.48) |
| Other Urinary Organs | 132 | 1.63#(1.36-1.93) | 34 | 4.31#(2.98-6.02) | 64 | 1.80#(1.38-2.29) | 31 | 1.07(0.73-1.52) | 3 | 0.34(0.07-1.01) |
| Brain/Other Nervous System | 87 | 1.58#(1.27-1.95) | 4 | 0.66(0.18-1.69) | 40 | 1.56#(1.12-2.13) | 33 | 1.80#(1.24-2.53) | 10 | 2.01(0.96-3.69) |
| lymphandblood | 277 | 1.18#(1.05-1.33) | 51 | 2.07#(1.54-2.73) | 116 | 1.1(0.9-1.31) | 80 | 0.99(0.79-1.23) | 30 | 1.31(0.88-1.87) |
| Miscellaneous Malignant Cancer | 562 | 3.17#(2.92-3.45) | 194 | 10.22#(8.83-11.76) | 215 | 2.66#(2.32-3.04) | 117 | 1.94#(1.61-2.33) | 36 | 2.11#(1.48-2.92) |
| In situ, benign or unknown behavior neoplasm | 108 | 1.77#(1.45-2.14) | 18 | 3.06#(1.81-4.83) | 46 | 1.73#(1.26-2.3) | 36 | 1.65#(1.15-2.28) | 8 | 1.21(0.52-2.39) |
| Noncancer | 7,356 | 1.08#(1.05-1.1) | 912 | 1.38#(1.3-1.48) | 2,920 | 0.98(0.95-1.02) | 2,705 | 1.11#(1.07-1.15) | 819 | 1.08#(1.01-1.16) |
| infections | 307 | 1.36#(1.21-1.52) | 63 | 2.64#(2.03-3.38) | 125 | 1.22#(1.02-1.45) | 95 | 1.22(0.99-1.5) | 24 | 1.08(0.69-1.61) |
| Diabetes Mellitus | 452 | 1.53#(1.4-1.68) | 59 | 1.93#(1.47-2.49) | 185 | 1.39#(1.2-1.61) | 160 | 1.57#(1.34-1.84) | 48 | 1.63#(1.2-2.17) |
| Alzheimers (ICD-9 and 10 only) | 238 | 0.82#(0.72-0.94) | 13 | 0.64(0.34-1.09) | 75 | 0.68#(0.54-0.86) | 107 | 0.93(0.77-1.13) | 43 | 0.98(0.71-1.32) |
| Diseases of Heart | 2,464 | 1.06#(1.02-1.1) | 317 | 1.37#(1.22-1.52) | 994 | 0.97(0.91-1.03) | 887 | 1.08#(1.01-1.15) | 266 | 1.06(0.94-1.2) |
| Hypertension without Heart Disease | 185 | 1.84#(1.59-2.13) | 14 | 1.59(0.87-2.67) | 66 | 1.56#(1.21-1.99) | 75 | 2.01#(1.58-2.52) | 30 | 2.46#(1.66-3.51) |
| Cerebrovascular Diseases | 482 | 1.04(0.95-1.13) | 66 | 1.49#(1.15-1.89) | 206 | 1.03(0.89-1.18) | 158 | 0.95(0.8-1.1) | 52 | 0.97(0.73-1.28) |
| Diseases of Arteries | 94 | 0.96(0.77-1.17) | 15 | 1.42(0.8-2.35) | 37 | 0.83(0.59-1.15) | 34 | 1.02(0.7-1.42) | 8 | 0.82(0.36-1.62) |
| Pneumonia and Influenza | 668 | 0.84#(0.78-0.91) | 74 | 0.96(0.75-1.21) | 269 | 0.78#(0.69-0.87) | 258 | 0.92(0.81-1.03) | 67 | 0.79#(0.61-1) |
| Digestive | 147 | 1.09(0.92-1.28) | 18 | 1.17(0.69-1.85) | 70 | 1.1(0.85-1.38) | 48 | 1.09(0.8-1.44) | 11 | 0.95(0.47-1.7) |
| Nephritis\ Nephrotic Syndrome\Nephrosis | 457 | 2.49#(2.26-2.72) | 41 | 2.31#(1.66-3.13) | 180 | 2.24#(1.92-2.59) | 170 | 2.58#(2.21-3) | 66 | 3.31#(2.56-4.21) |
| Pregnancy\Childbirth\Puerperium | 21 | 2.19#(1.35-3.34) | 1 | 0.91(0.02-5.05) | 10 | 2.20#(1.06-4.05) | 7 | 2.24(0.9-4.61) | 3 | 3.6(0.74-10.53) |
| Symptoms, Signs and Ill-Defined Conditions | 95 | 1.04(0.84-1.27) | 14 | 1.6(0.87-2.68) | 41 | 1.03(0.74-1.39) | 35 | 1.05(0.73-1.46) | 5 | 0.53(0.17-1.23) |
| Accidents\Adverse Effects\Homicide\Legal intervention | 276 | 0.86#(0.76-0.96) | 30 | 0.89(0.6-1.26) | 124 | 0.85(0.7-1.01) | 95 | 0.86(0.7-1.06) | 27 | 0.84(0.56-1.23) |
| Suicide\Self-Inflicted Injury | 75 | 0.84(0.66-1.05) | 8 | 0.75(0.32-1.47) | 32 | 0.74(0.51-1.04) | 28 | 0.98(0.65-1.42) | 7 | 0.98(0.39-2.02) |
| Other Cause of Death | 1,395 | 1(0.94-1.05) | 179 | 1.45#(1.24-1.68) | 506 | 0.86#(0.78-0.94) | 548 | 1.05(0.97-1.14) | 162 | 0.97(0.82-1.13) |

Supplementary Table 9. Standardized-mortality ratios (SMRs) for each cause of death following RCC diagnosis in never married patients.

|  | Timing of Death After Diagnosis | | | | | | | | | |
| --- | --- | --- | --- | --- | --- | --- | --- | --- | --- | --- |
|  | All Y ears | | <1 y | | 1 to <5 y | | 5 to <10 y | | ≥10 y | |
| All Causes of Death | 5,691 | 4.11#(4-4.21) | 1,875 | 12.23#(11.69-12.8) | 2,457 | 3.85#(3.69-4) | 1,076 | 2.30#(2.17-2.45) | 283 | 2.24#(1.98-2.51) |
| All Malignant Cancers | 3,589 | 10.05#(9.72-10.38) | 1,522 | 36.68#(34.86-38.57) | 1,509 | 8.91#(8.47-9.37) | 446 | 3.82#(3.47-4.19) | 112 | 3.80#(3.13-4.57) |
| Oral Cavity/Pharynx/eye/endocrine | 11 | 1.34(0.67-2.41) | 1 | 1.05(0.03-5.87) | 5 | 1.28(0.42-2.99) | 3 | 1.13(0.23-3.3) | 2 | 2.97(0.36-10.74) |
| Digestive System | 133 | 1.37#(1.15-1.62) | 16 | 1.43(0.82-2.33) | 58 | 1.26(0.96-1.63) | 42 | 1.32(0.95-1.78) | 17 | 2.10#(1.23-3.37) |
| Respiratory System | 147 | 1.44#(1.21-1.69) | 29 | 2.34#(1.57-3.36) | 63 | 1.28(0.98-1.64) | 44 | 1.34(0.98-1.8) | 11 | 1.39(0.69-2.49) |
| Bones and Joints/soft tissue/skin | 14 | 1.35(0.74-2.27) | 4 | 3.33(0.91-8.54) | 5 | 1.02(0.33-2.38) | 4 | 1.18(0.32-3.03) | 1 | 1.16(0.03-6.48) |
| Breast | 9 | 0.50#(0.23-0.95) | 1 | 0.47(0.01-2.63) | 2 | 0.23#(0.03-0.84) | 4 | 0.69(0.19-1.76) | 2 | 1.39(0.17-5.02) |
| Genital System | 48 | 1.38#(1.02-1.83) | 12 | 3.15#(1.63-5.5) | 22 | 1.37(0.86-2.08) | 12 | 1.02(0.53-1.79) | 2 | 0.64(0.08-2.32) |
| Kidney and Renal Pelvis | 2,991 | 338.10#(326.09-350.44) | 1,377 | 1,337.65#(1267.93-1410.22) | 1,254 | 298.30#(282.01-315.28) | 296 | 102.56#(91.21-114.94) | 64 | 88.02#(67.78-112.39) |
| Other Urinary Organs | 28 | 2.84#(1.89-4.11) | 4 | 3.82#(1.04-9.77) | 18 | 4.03#(2.39-6.37) | 4 | 1.17(0.32-3.01) | 2 | 2.14(0.26-7.72) |
| Brain/Other Nervous System | 17 | 1.93#(1.12-3.09) | 3 | 2.92(0.6-8.52) | 11 | 2.60#(1.3-4.65) | 3 | 1.05(0.22-3.08) | 0 | 0(0-5.2) |
| lymphandblood | 53 | 1.62#(1.21-2.12) | 13 | 3.49#(1.86-5.97) | 22 | 1.44(0.9-2.18) | 15 | 1.38(0.77-2.27) | 3 | 1.06(0.22-3.11) |
| Miscellaneous Malignant Cancer | 138 | 5.26#(4.42-6.21) | 62 | 20.45#(15.68-26.21) | 49 | 3.96#(2.93-5.24) | 19 | 2.20#(1.33-3.44) | 8 | 3.60#(1.56-7.1) |
| In situ, benign or unknown behavior neoplasm | 22 | 2.75#(1.72-4.16) | 9 | 10.51#(4.8-19.94) | 10 | 2.76#(1.32-5.07) | 3 | 1.09(0.22-3.17) | 0 | 0(0-4.87) |
| Noncancer | 2,080 | 2.04#(1.95-2.13) | 344 | 3.10#(2.78-3.45) | 938 | 2.01#(1.89-2.15) | 627 | 1.80#(1.67-1.95) | 171 | 1.78#(1.52-2.06) |
| infections | 126 | 3.15#(2.63-3.75) | 26 | 5.40#(3.53-7.92) | 51 | 2.67#(1.99-3.51) | 41 | 3.20#(2.3-4.34) | 8 | 2.47#(1.07-4.87) |
| Diabetes Mellitus | 144 | 2.98#(2.51-3.5) | 25 | 4.59#(2.97-6.78) | 63 | 2.77#(2.13-3.55) | 43 | 2.68#(1.94-3.61) | 13 | 3.10#(1.65-5.3) |
| Alzheimers (ICD-9 and 10 only) | 38 | 1.03(0.73-1.42) | 2 | 0.66(0.08-2.38) | 18 | 1.24(0.73-1.96) | 12 | 0.85(0.44-1.48) | 6 | 1.2(0.44-2.62) |
| Diseases of Heart | 642 | 1.90#(1.75-2.05) | 99 | 2.63#(2.14-3.21) | 287 | 1.85#(1.64-2.07) | 200 | 1.75#(1.52-2.01) | 56 | 1.78#(1.35-2.32) |
| Hypertension without Heart Disease | 64 | 3.87#(2.98-4.95) | 11 | 6.57#(3.28-11.76) | 29 | 3.94#(2.64-5.66) | 18 | 3.10#(1.84-4.9) | 6 | 3.58#(1.31-7.8) |
| Cerebrovascular Diseases | 98 | 1.43#(1.16-1.74) | 15 | 2.04#(1.14-3.36) | 43 | 1.39#(1.01-1.87) | 27 | 1.14(0.75-1.66) | 13 | 1.92#(1.02-3.28) |
| Diseases of Arteries | 30 | 2.16#(1.46-3.09) | 4 | 2.49(0.68-6.38) | 17 | 2.63#(1.53-4.22) | 7 | 1.53(0.61-3.15) | 2 | 1.64(0.2-5.91) |
| Pneumonia and Influenza | 174 | 1.65#(1.42-1.92) | 27 | 2.43#(1.6-3.53) | 76 | 1.60#(1.26-2) | 58 | 1.59#(1.21-2.06) | 13 | 1.29(0.69-2.21) |
| Digestive | 66 | 2.61#(2.02-3.32) | 9 | 2.96#(1.35-5.61) | 36 | 2.91#(2.04-4.03) | 17 | 2.14#(1.25-3.42) | 4 | 2.08(0.57-5.32) |
| Nephritis\ Nephrotic Syndrome\Nephrosis | 113 | 4.03#(3.32-4.85) | 24 | 7.95#(5.09-11.82) | 51 | 3.99#(2.97-5.24) | 32 | 3.34#(2.29-4.72) | 6 | 2.3(0.84-5) |
| Pregnancy\Childbirth\Puerperium | 11 | 5.39#(2.69-9.65) | 2 | 7.8(0.94-28.17) | 5 | 4.95#(1.61-11.56) | 3 | 4.8(0.99-14.04) | 1 | 6.7(0.17-37.33) |
| Symptoms, Signs and Ill-Defined Conditions | 47 | 3.30#(2.43-4.39) | 13 | 8.15#(4.34-13.94) | 18 | 2.75#(1.63-4.35) | 15 | 3.09#(1.73-5.1) | 1 | 0.81(0.02-4.49) |
| Accidents\Adverse Effects\Homicide\Legal intervention | 134 | 2.11#(1.77-2.5) | 20 | 2.61#(1.6-4.03) | 73 | 2.37#(1.86-2.98) | 32 | 1.61#(1.1-2.27) | 9 | 1.78(0.82-3.38) |
| Suicide\Self-Inflicted Injury | 31 | 1.75#(1.19-2.49) | 5 | 2.19(0.71-5.11) | 21 | 2.37#(1.46-3.62) | 4 | 0.75(0.21-1.93) | 1 | 0.83(0.02-4.61) |
| Other Cause of Death | 362 | 1.79#(1.61-1.99) | 62 | 3.04#(2.33-3.9) | 150 | 1.68#(1.42-1.97) | 118 | 1.65#(1.37-1.98) | 32 | 1.55#(1.06-2.2) |

Supplementary Table 10. Standardized-mortality ratios (SMRs) for each cause of death following RCC diagnosis in other marital status patients.

|  | Timing of Death After Diagnosis | | | | | | | | | |
| --- | --- | --- | --- | --- | --- | --- | --- | --- | --- | --- |
|  | All Y ears | | <1 y | | 1 to <5 y | | 5 to <10 y | | ≥10 y | |
| All Causes of Death | 11,757 | 2.74#(2.69-2.79) | 3,579 | 6.96#(6.73-7.19) | 4,975 | 2.41#(2.35-2.48) | 2,598 | 1.88#(1.81-1.96) | 605 | 1.84#(1.69-1.99) |
| All Malignant Cancers | 6,789 | 7.43#(7.25-7.61) | 2,840 | 24.09#(23.22-25) | 2,845 | 6.28#(6.05-6.52) | 915 | 3.26#(3.05-3.48) | 189 | 3.01#(2.6-3.47) |
| Oral Cavity/Pharynx/eye/endocrine | 24 | 1.39(0.89-2.07) | 5 | 2.31(0.75-5.38) | 10 | 1.17(0.56-2.16) | 7 | 1.31(0.53-2.7) | 2 | 1.63(0.2-5.9) |
| Digestive System | 316 | 1.35#(1.21-1.51) | 54 | 1.82#(1.37-2.38) | 135 | 1.17(0.98-1.39) | 101 | 1.40#(1.14-1.7) | 26 | 1.60#(1.04-2.34) |
| Respiratory System | 372 | 1.48#(1.34-1.64) | 65 | 1.92#(1.48-2.45) | 176 | 1.39#(1.2-1.62) | 102 | 1.36#(1.11-1.66) | 29 | 1.82#(1.22-2.61) |
| Bones and Joints/soft tissue/skin | 33 | 1.33(0.92-1.87) | 14 | 4.50#(2.46-7.54) | 12 | 0.98(0.51-1.72) | 6 | 0.78(0.29-1.69) | 1 | 0.56(0.01-3.15) |
| Breast | 30 | 0.48#(0.32-0.68) | 4 | 0.5(0.14-1.27) | 8 | 0.26#(0.11-0.51) | 11 | 0.56(0.28-1.01) | 7 | 1.64(0.66-3.37) |
| Genital System | 64 | 0.66#(0.51-0.85) | 12 | 0.99(0.51-1.72) | 30 | 0.64#(0.43-0.91) | 15 | 0.50#(0.28-0.83) | 7 | 1.01(0.41-2.08) |
| Kidney and Renal Pelvis | 5,429 | 262.31#(255.38-269.39) | 2,491 | 939.39#(902.86-977.02) | 2,271 | 221.60#(212.58-230.91) | 570 | 89.61#(82.4-97.27) | 97 | 67.57#(54.8-82.43) |
| Other Urinary Organs | 70 | 2.51#(1.95-3.17) | 30 | 8.94#(6.03-12.76) | 30 | 2.23#(1.5-3.18) | 8 | 0.9(0.39-1.76) | 2 | 0.92(0.11-3.34) |
| Brain/Other Nervous System | 37 | 1.98#(1.39-2.73) | 11 | 4.60#(2.29-8.23) | 16 | 1.72(0.98-2.79) | 10 | 1.75(0.84-3.22) | 0 | 0(0-2.9) |
| lymphandblood | 110 | 1.2(0.99-1.45) | 23 | 1.99#(1.26-2.98) | 47 | 1.05(0.77-1.39) | 35 | 1.23(0.85-1.71) | 5 | 0.77(0.25-1.79) |
| Miscellaneous Malignant Cancer | 304 | 4.35#(3.88-4.87) | 131 | 14.71#(12.3-17.45) | 110 | 3.20#(2.63-3.86) | 50 | 2.32#(1.72-3.05) | 13 | 2.64#(1.4-4.51) |
| In situ, benign or unknown behavior neoplasm | 45 | 1.70#(1.24-2.27) | 11 | 3.46#(1.73-6.2) | 18 | 1.41(0.84-2.23) | 13 | 1.52(0.81-2.6) | 3 | 1.48(0.31-4.33) |
| Noncancer | 4,923 | 1.47#(1.43-1.51) | 728 | 1.85#(1.72-1.99) | 2,112 | 1.32#(1.27-1.38) | 1,670 | 1.53#(1.46-1.61) | 413 | 1.56#(1.41-1.72) |
| infections | 173 | 1.70#(1.46-1.98) | 28 | 2.21#(1.47-3.19) | 74 | 1.49#(1.17-1.87) | 53 | 1.67#(1.25-2.18) | 18 | 2.48#(1.47-3.92) |
| Diabetes Mellitus | 312 | 2.48#(2.21-2.77) | 42 | 2.66#(1.92-3.6) | 144 | 2.33#(1.97-2.75) | 106 | 2.70#(2.21-3.26) | 20 | 2.20#(1.34-3.4) |
| Alzheimers (ICD-9 and 10 only) | 169 | 0.81#(0.69-0.94) | 13 | 0.63(0.34-1.08) | 59 | 0.63#(0.48-0.81) | 78 | 1.04(0.83-1.3) | 19 | 0.92(0.55-1.43) |
| Diseases of Heart | 1,648 | 1.49#(1.42-1.57) | 261 | 1.94#(1.71-2.19) | 709 | 1.33#(1.24-1.44) | 547 | 1.55#(1.42-1.68) | 131 | 1.54#(1.29-1.83) |
| Hypertension without Heart Disease | 134 | 2.33#(1.95-2.76) | 17 | 2.68#(1.56-4.29) | 52 | 1.93#(1.44-2.53) | 54 | 2.78#(2.09-3.63) | 11 | 2.26#(1.13-4.04) |
| Cerebrovascular Diseases | 363 | 1.39#(1.25-1.54) | 45 | 1.45#(1.06-1.95) | 173 | 1.40#(1.2-1.62) | 116 | 1.37#(1.13-1.64) | 29 | 1.38(0.93-1.99) |
| Diseases of Arteries | 70 | 1.47#(1.14-1.86) | 14 | 2.25#(1.23-3.77) | 24 | 1.02(0.66-1.52) | 27 | 1.84#(1.21-2.68) | 5 | 1.49(0.48-3.48) |
| Pneumonia and Influenza | 494 | 1.36#(1.24-1.48) | 63 | 1.43#(1.1-1.84) | 205 | 1.17#(1.01-1.34) | 191 | 1.63#(1.41-1.88) | 35 | 1.27(0.88-1.76) |
| Digestive | 81 | 1.88#(1.5-2.34) | 13 | 2.33#(1.24-3.98) | 36 | 1.67#(1.17-2.31) | 28 | 2.16#(1.44-3.13) | 4 | 1.41(0.38-3.6) |
| Nephritis\ Nephrotic Syndrome\Nephrosis | 289 | 3.26#(2.89-3.65) | 41 | 3.83#(2.75-5.2) | 117 | 2.73#(2.26-3.27) | 104 | 3.65#(2.99-4.43) | 27 | 4.00#(2.63-5.81) |
| Pregnancy\Childbirth\Puerperium | 10 | 2.84#(1.36-5.23) | 3 | 6.53#(1.35-19.08) | 4 | 2.27(0.62-5.81) | 3 | 2.83(0.58-8.27) | 0 | 0(0-15.81) |
| Symptoms, Signs and Ill-Defined Conditions | 76 | 1.40#(1.1-1.75) | 15 | 2.32#(1.3-3.83) | 38 | 1.45#(1.02-1.99) | 19 | 1.07(0.64-1.67) | 4 | 1.06(0.29-2.71) |
| Accidents\Adverse Effects\Homicide\Legal intervention | 172 | 1.37#(1.17-1.59) | 24 | 1.59#(1.02-2.37) | 74 | 1.21(0.95-1.52) | 56 | 1.39#(1.05-1.81) | 18 | 1.88#(1.11-2.96) |
| Suicide\Self-Inflicted Injury | 34 | 1.50#(1.04-2.09) | 8 | 2.58#(1.12-5.09) | 16 | 1.37(0.79-2.23) | 9 | 1.37(0.63-2.6) | 1 | 0.71(0.02-3.97) |
| Other Cause of Death | 898 | 1.22#(1.14-1.3) | 141 | 1.75#(1.47-2.06) | 387 | 1.12#(1.01-1.24) | 279 | 1.12(0.99-1.26) | 91 | 1.48#(1.19-1.82) |

Supplementary Table 11. Standardized-mortality ratios (SMRs) for each cause of death following RCC diagnosis in patients with surgery.

|  | Timing of Death After Diagnosis | | | | | | | | | |
| --- | --- | --- | --- | --- | --- | --- | --- | --- | --- | --- |
|  | All Y ears | | <1 y | | 1 to <5 y | | 5 to <10 y | | ≥10 y | |
| All Causes of Death | 27,661 | 2.04#(2.02-2.07） | 4,606 | 3.60#(3.49-3.7） | 12,793 | 2.13#(2.09-2.16） | 8,067 | 1.67#(1.63-1.7） | 2,195 | 1.56#(1.5-1.63） |
| All Malignant Cancers | 15,751 | 4.69#(4.62-4.76） | 3,485 | 9.92#(9.59-10.25） | 7,981 | 5.12#(5.01-5.24） | 3,445 | 3.01#(2.91-3.11） | 840 | 2.75#(2.56-2.94） |
| Oral Cavity/Pharynx/eye/endocrine | 60 | 0.84(0.64-1.08） | 8 | 1.08(0.47-2.13） | 25 | 0.76(0.49-1.12） | 18 | 0.74(0.44-1.18） | 9 | 1.38(0.63-2.61） |
| Digestive System | 863 | 0.99(0.92-1.05） | 57 | 0.63#(0.48-0.82） | 358 | 0.88#(0.79-0.98） | 336 | 1.12#(1.01-1.25） | 112 | 1.40#(1.15-1.68） |
| Respiratory System | 924 | 0.95(0.89-1.01） | 51 | 0.47#(0.35-0.62） | 423 | 0.92(0.83-1.01） | 350 | 1.09(0.98-1.21） | 100 | 1.23#(1-1.5） |
| Bones and Joints/soft tissue/skin | 96 | 0.95(0.77-1.16） | 15 | 1.46(0.82-2.4） | 39 | 0.84(0.6-1.15） | 32 | 0.92(0.63-1.3） | 10 | 1.05(0.5-1.93） |
| Breast | 61 | 0.41#(0.31-0.53） | 1 | 0.06#(0-0.35） | 17 | 0.25#(0.14-0.39） | 29 | 0.58#(0.39-0.83） | 14 | 1.06(0.58-1.79） |
| Genital System | 190 | 0.56#(0.48-0.65） | 11 | 0.34#(0.17-0.6） | 85 | 0.56#(0.45-0.69） | 68 | 0.57#(0.44-0.72） | 26 | 0.76(0.5-1.11） |
| Kidney and Renal Pelvis | 12,381 | 148.13#(145.53-150.76） | 3,187 | 363.90#(351.38-376.76） | 6,500 | 167.53#(163.48-171.65） | 2,232 | 78.50#(75.27-81.82） | 462 | 60.87#(55.44-66.68） |
| Other Urinary Organs | 153 | 1.41#(1.2-1.65） | 24 | 2.42#(1.55-3.59） | 80 | 1.68#(1.33-2.09） | 42 | 1.07(0.77-1.45） | 7 | 0.61(0.24-1.25） |
| Brain/Other Nervous System | 125 | 1.61#(1.34-1.91） | 8 | 0.96(0.41-1.89） | 62 | 1.70#(1.3-2.17） | 45 | 1.73#(1.26-2.31） | 10 | 1.46(0.7-2.68） |
| lymphandblood | 303 | 0.92(0.82-1.03） | 24 | 0.72(0.46-1.07） | 127 | 0.84(0.7-1） | 116 | 1.01(0.83-1.21） | 36 | 1.14(0.8-1.58） |
| Miscellaneous Malignant Cancer | 595 | 2.36#(2.17-2.56） | 99 | 3.81#(3.1-4.64） | 265 | 2.28#(2.02-2.58） | 177 | 2.04#(1.75-2.37） | 54 | 2.28#(1.71-2.97） |
| In situ, benign or unknown behavior neoplasm | 121 | 1.40#(1.16-1.67） | 13 | 1.64(0.87-2.81） | 54 | 1.42#(1.07-1.85） | 44 | 1.40#(1.02-1.88） | 10 | 1.09(0.52-2.01） |
| Noncancer | 11,789 | 1.17#(1.15-1.19） | 1,108 | 1.20#(1.13-1.28） | 4,758 | 1.08#(1.05-1.11） | 4,578 | 1.25#(1.21-1.29） | 1,345 | 1.24#(1.17-1.3） |
| infections | 491 | 1.46#(1.33-1.59） | 65 | 1.89#(1.46-2.41） | 205 | 1.33#(1.15-1.52） | 172 | 1.48#(1.26-1.71） | 49 | 1.53#(1.13-2.03） |
| Diabetes Mellitus | 766 | 1.77#(1.65-1.9） | 75 | 1.74#(1.37-2.18） | 324 | 1.65#(1.47-1.84） | 286 | 1.91#(1.69-2.14） | 81 | 1.94#(1.54-2.41） |
| Alzheimers (ICD-9 and 10 only) | 353 | 0.76#(0.68-0.84） | 4 | 0.13#(0.04-0.34） | 104 | 0.58#(0.48-0.71） | 182 | 0.97(0.83-1.12） | 63 | 0.93(0.72-1.19） |
| Diseases of Heart | 3,879 | 1.14#(1.11-1.18） | 389 | 1.22#(1.1-1.35） | 1,573 | 1.05(1-1.1） | 1,488 | 1.22#(1.16-1.29） | 429 | 1.20#(1.09-1.32） |
| Hypertension without Heart Disease | 331 | 2.12#(1.9-2.36） | 25 | 1.94#(1.25-2.86） | 125 | 1.89#(1.57-2.25） | 134 | 2.28#(1.91-2.7） | 47 | 2.58#(1.9-3.43） |
| Cerebrovascular Diseases | 777 | 1.09#(1.02-1.17） | 79 | 1.24(0.98-1.54） | 341 | 1.11(0.99-1.23） | 268 | 1.03(0.91-1.17） | 89 | 1.13(0.9-1.39） |
| Diseases of Arteries | 157 | 1.09(0.93-1.28） | 19 | 1.31(0.79-2.04） | 64 | 0.98(0.76-1.25） | 59 | 1.18(0.9-1.53） | 15 | 1.08(0.6-1.77） |
| Pneumonia and Influenza | 1,058 | 0.92#(0.87-0.98） | 70 | 0.66#(0.52-0.84） | 415 | 0.82#(0.74-0.9） | 461 | 1.12#(1.02-1.22） | 112 | 0.93(0.77-1.12） |
| Digestive | 246 | 1.27#(1.12-1.44） | 26 | 1.21(0.79-1.78） | 116 | 1.26#(1.04-1.51） | 85 | 1.34#(1.07-1.66） | 19 | 1.18(0.71-1.84） |
| Nephritis\ Nephrotic Syndrome\Nephrosis | 754 | 2.78#(2.59-2.99） | 65 | 2.62#(2.02-3.34） | 303 | 2.54#(2.26-2.84） | 291 | 2.97#(2.64-3.33） | 95 | 3.33#(2.69-4.07） |
| Pregnancy\Childbirth\Puerperium | 34 | 2.38#(1.65-3.32） | 3 | 1.87(0.38-5.45） | 15 | 2.19#(1.23-3.62） | 12 | 2.58#(1.33-4.5） | 4 | 3.35(0.91-8.58） |
| Symptoms, Signs and Ill-Defined Conditions | 168 | 1.19#(1.01-1.38） | 22 | 1.73#(1.08-2.62） | 75 | 1.2(0.95-1.51） | 62 | 1.18(0.91-1.52） | 9 | 0.64(0.29-1.21） |
| Accidents\Adverse Effects\Homicide\Legal intervention | 502 | 1.06(0.97-1.16） | 52 | 1.08(0.81-1.42） | 224 | 1.03(0.9-1.18） | 173 | 1.06(0.91-1.23） | 53 | 1.16(0.87-1.52） |
| Suicide\Self-Inflicted Injury | 125 | 1.01(0.84-1.2） | 11 | 0.76(0.38-1.36） | 64 | 1.06(0.82-1.35） | 41 | 1.04(0.75-1.41） | 9 | 0.94(0.43-1.78） |
| Other Cause of Death | 2,148 | 1.02(0.98-1.07） | 203 | 1.17#(1.01-1.34） | 810 | 0.91#(0.85-0.98） | 864 | 1.09#(1.02-1.17） | 271 | 1.12(0.99-1.26） |

Supplementary Table 12. Standardized-mortality ratios (SMRs) for each cause of death following RCC diagnosis in patients without surgery.

|  | Timing of Death After Diagnosis | | | | | | | | | |
| --- | --- | --- | --- | --- | --- | --- | --- | --- | --- | --- |
|  | All Y ears | | <1 y | | 1 to <5 y | | 5 to <10 y | | ≥10 y | |
| All Causes of Death | 11,969 | 8.78#（8.62-8.93） | 7,136 | 23.26#（22.72-23.8） | 4,038 | 5.32#（5.15-5.48） | 703 | 2.68#（2.48-2.88） | 92 | 2.64#（2.13-3.23） |
| All Malignant Cancers | 9,345 | 35.74#（35.02-36.47） | 6,235 | 98.87#（96.43-101.36） | 2,806 | 19.40#（18.69-20.13） | 271 | 5.70#（5.04-6.42） | 33 | 5.27#（3.63-7.41） |
| Oral Cavity/Pharynx/eye/endocrine | 11 | 2.26#（1.13-4.05） | 8 | 6.89#（2.97-13.58） | 2 | 0.74（0.09-2.69） | 1 | 1.12（0.03-6.23） | 0 | 0（0-31.05） |
| Digestive System | 199 | 3.00#（2.6-3.45） | 100 | 6.27#（5.11-7.63） | 76 | 2.07#（1.63-2.59） | 20 | 1.66#（1.01-2.56） | 3 | 1.89（0.39-5.51） |
| Respiratory System | 281 | 4.10#（3.63-4.61） | 153 | 8.80#（7.46-10.31） | 97 | 2.57#（2.08-3.13） | 27 | 2.27#（1.5-3.31） | 4 | 2.66（0.72-6.8） |
| Bones and Joints/soft tissue/skin | 30 | 3.86#（2.6-5.51） | 22 | 12.01#（7.53-18.18） | 7 | 1.62（0.65-3.33） | 0 | 0（0-2.57） | 1 | 5.39（0.14-30.01） |
| Breast | 10 | 0.85（0.41-1.56） | 5 | 1.84（0.6-4.3） | 1 | 0.15#（0-0.86） | 3 | 1.32（0.27-3.84） | 1 | 3.39（0.09-18.89） |
| Genital System | 61 | 1.90#（1.45-2.44） | 31 | 4.15#（2.82-5.89） | 21 | 1.18（0.73-1.81） | 6 | 1（0.37-2.17） | 3 | 3.5（0.72-10.21） |
| Kidney and Renal Pelvis | 8,114 | 1,314.22#（1285.78-1343.13） | 5,511 | 3,695.30#（3598.37-3794.18） | 2,398 | 701.60#（673.8-730.25） | 189 | 169.05#（145.81-194.95） | 16 | 109.04#（62.33-177.07） |
| Other Urinary Organs | 77 | 7.37#（5.82-9.21） | 44 | 18.64#（13.54-25.02） | 32 | 5.50#（3.76-7.76） | 1 | 0.5（0.01-2.79） | 0 | 0（0-13.69） |
| Brain/Other Nervous System | 16 | 3.45#（1.97-5.6） | 10 | 8.78#（4.21-16.14） | 5 | 1.96（0.64-4.57） | 1 | 1.2（0.03-6.7） | 0 | 0（0-33.47） |
| lymphandblood | 137 | 4.89#（4.1-5.78） | 63 | 9.51#（7.31-12.17） | 58 | 3.73#（2.83-4.82） | 14 | 2.71#（1.48-4.54） | 2 | 2.96（0.36-10.7） |
| Miscellaneous Malignant Cancer | 409 | 19.66#（17.8-21.67） | 288 | 58.15#（51.63-65.27） | 109 | 9.46#（7.76-11.41） | 9 | 2.36#（1.08-4.48） | 3 | 5.98#（1.23-17.48） |
| In situ, benign or unknown behavior neoplasm | 54 | 6.08#（4.57-7.94） | 25 | 12.52#（8.1-18.48） | 20 | 4.04#（2.47-6.24） | 8 | 4.70#（2.03-9.26） | 1 | 4.49（0.11-25.04） |
| Noncancer | 2,570 | 2.35#（2.26-2.44） | 876 | 3.62#（3.39-3.87） | 1,212 | 1.99#（1.88-2.1） | 424 | 1.99#（1.8-2.19） | 58 | 2.04#（1.55-2.64） |
| infections | 115 | 3.81#（3.15-4.58） | 52 | 7.45#（5.56-9.77） | 45 | 2.69#（1.96-3.59） | 17 | 3.00#（1.75-4.8） | 1 | 1.32（0.03-7.37） |
| Diabetes Mellitus | 142 | 3.80#（3.2-4.48） | 51 | 5.86#（4.37-7.71） | 68 | 3.29#（2.55-4.17） | 23 | 3.27#（2.08-4.91） | 0 | 0（0-3.84） |
| Alzheimers (ICD-9 and 10 only) | 92 | 1.30#（1.04-1.59） | 24 | 1.75#（1.12-2.6） | 48 | 1.21（0.89-1.6） | 15 | 0.98（0.55-1.61） | 5 | 2.31（0.75-5.39） |
| Diseases of Heart | 875 | 2.33#（2.18-2.49） | 288 | 3.38#（3-3.79） | 417 | 1.99#（1.8-2.19） | 146 | 2.04#（1.73-2.4） | 24 | 2.55#（1.64-3.8） |
| Hypertension without Heart Disease | 52 | 2.78#（2.08-3.65） | 17 | 4.36#（2.54-6.99） | 22 | 2.11#（1.32-3.2） | 13 | 3.38#（1.8-5.78） | 0 | 0（0-6.88） |
| Cerebrovascular Diseases | 166 | 1.97#（1.68-2.29） | 47 | 2.51#（1.84-3.33） | 81 | 1.72#（1.37-2.14） | 33 | 2.01#（1.38-2.82） | 5 | 2.26（0.73-5.27） |
| Diseases of Arteries | 37 | 2.32#（1.63-3.19） | 14 | 3.63#（1.99-6.1） | 14 | 1.57（0.86-2.64） | 9 | 3.15#（1.44-5.98） | 0 | 0（0-10.27） |
| Pneumonia and Influenza | 278 | 2.40#（2.13-2.7） | 94 | 3.56#（2.88-4.36） | 135 | 2.09#（1.75-2.48） | 46 | 2.10#（1.54-2.8） | 3 | 1.05（0.22-3.07） |
| Digestive | 48 | 4.65#（3.42-6.16） | 14 | 5.39#（2.94-9.04） | 26 | 4.58#（2.99-6.71） | 8 | 4.41#（1.9-8.69） | 0 | 0（0-15.27） |
| Nephritis\ Nephrotic Syndrome\Nephrosis | 105 | 3.53#（2.88-4.27） | 41 | 6.14#（4.4-8.32） | 45 | 2.71#（1.98-3.63） | 15 | 2.62#（1.47-4.32） | 4 | 5.11#（1.39-13.09） |
| Pregnancy\Childbirth\Puerperium | 8 | 9.22#（3.98-18.16） | 3 | 14.09#（2.91-41.18） | 4 | 8.37#（2.28-21.43） | 1 | 6.43（0.16-35.8） | 0 | 0（0-172.3） |
| Symptoms, Signs and Ill-Defined Conditions | 50 | 2.71#（2.01-3.58） | 20 | 4.87#（2.97-7.51） | 22 | 2.12#（1.33-3.21） | 7 | 1.98（0.8-4.08） | 1 | 2.48（0.06-13.85） |
| Accidents\Adverse Effects\Homicide\Legal intervention | 80 | 2.10#（1.66-2.61） | 22 | 2.58#（1.62-3.91） | 47 | 2.22#（1.63-2.95） | 10 | 1.35（0.65-2.48） | 1 | 0.98（0.02-5.47） |
| Suicide\Self-Inflicted Injury | 15 | 2.45#（1.37-4.05） | 10 | 6.38#（3.06-11.73） | 5 | 1.49（0.48-3.48） | 0 | 0（0-3.53） | 0 | 0（0-25.38） |
| Other Cause of Death | 507 | 2.11#（1.93-2.3） | 179 | 3.55#（3.05-4.11） | 233 | 1.73#（1.52-1.97） | 81 | 1.65#（1.31-2.05） | 14 | 2.13#（1.17-3.58） |

Supplementary Table 13. Standardized-mortality ratios (SMRs) for each cause of death following RCC diagnosis in patients with radiation.

|  | Timing of Death After Diagnosis | | | | | | | | | |
| --- | --- | --- | --- | --- | --- | --- | --- | --- | --- | --- |
|  | All Y ears | | <1 y | | 1 to <5 y | | 5 to <10 y | | ≥10 y | |
| All Causes of Death | 4,581 | 28.47#（27.65-29.3） | 2,666 | 54.38#（52.33-56.48） | 1,713 | 21.48#（20.48-22.52） | 176 | 6.54#（5.61-7.58） | 26 | 4.96#（3.24-7.27） |
| All Malignant Cancers | 4,309 | 102.56#（99.52-105.67） | 2,547 | 193.32#（185.89-200.98） | 1,595 | 75.87#（72.19-79.68） | 148 | 22.40#（18.94-26.32） | 19 | 15.70#（9.46-24.53） |
| Oral Cavity/Pharynx/eye/endocrine | 2 | 2.16（0.26-7.79） | 1 | 3.49（0.09-19.43） | 1 | 2.14（0.05-11.9） | 0 | 0（0-25.25） | 0 | 0（0-139.94） |
| Digestive System | 29 | 2.63#（1.76-3.78） | 13 | 3.79#（2.02-6.48） | 9 | 1.62（0.74-3.08） | 5 | 2.88（0.94-6.73） | 2 | 6.27（0.76-22.65） |
| Respiratory System | 52 | 4.13#（3.08-5.42） | 30 | 7.41#（5-10.58） | 16 | 2.53#（1.45-4.11） | 5 | 2.64（0.86-6.17） | 1 | 3.03（0.08-16.9） |
| Bones and Joints/soft tissue/skin | 13 | 9.81#（5.22-16.77） | 6 | 14.81#（5.43-32.22） | 7 | 10.50#（4.22-21.63） | 0 | 0（0-17.08） | 0 | 0（0-97.79） |
| Breast | 2 | 1.38（0.17-4.99） | 1 | 2.05（0.05-11.45） | 1 | 1.44（0.04-8.04） | 0 | 0（0-17.2） | 0 | 0（0-68.13） |
| Genital System | 22 | 5.55#（3.48-8.4） | 14 | 11.51#（6.29-19.32） | 7 | 3.56#（1.43-7.34） | 1 | 1.51（0.04-8.44） | 0 | 0（0-29.65） |
| Kidney and Renal Pelvis | 4,009 | 3,656.69#（3544.37-3771.67） | 2,368 | 6,955.85#（6678.48-7241.78） | 1,501 | 2,716.55#（2580.84-2857.54） | 127 | 733.37#（611.38-872.58） | 13 | 430.45#（229.2-736.08） |
| Other Urinary Organs | 22 | 16.20#（10.15-24.53） | 14 | 35.24#（19.26-59.12） | 8 | 11.80#（5.09-23.25） | 0 | 0（0-15.42） | 0 | 0（0-85.15） |
| Brain/Other Nervous System | 7 | 6.85#（2.75-14.11） | 4 | 12.55#（3.42-32.15） | 1 | 1.94（0.05-10.78） | 2 | 12.63#（1.53-45.61） | 0 | 0（0-128.19） |
| lymphandblood | 14 | 3.42#（1.87-5.73） | 5 | 3.97#（1.29-9.26） | 6 | 2.93#（1.08-6.39） | 2 | 2.99（0.36-10.79） | 1 | 8.08（0.2-45.02） |
| Miscellaneous Malignant Cancer | 137 | 43.37#（36.41-51.27） | 91 | 92.41#（74.4-113.46） | 38 | 24.08#（17.04-33.05） | 6 | 11.92#（4.37-25.94） | 2 | 21.61#（2.62-78.07） |
| In situ, benign or unknown behavior neoplasm | 15 | 14.57#（8.15-24.03） | 10 | 32.64#（15.65-60.03） | 5 | 9.82#（3.19-22.91） | 0 | 0（0-20.57） | 0 | 0（0-106.69） |
| Noncancer | 257 | 2.18#（1.92-2.46） | 109 | 3.07#（2.52-3.7） | 113 | 1.94#（1.6-2.33） | 28 | 1.39（0.92-2.01） | 7 | 1.75（0.7-3.61） |
| infections | 24 | 5.93#（3.8-8.83） | 13 | 10.15#（5.41-17.36） | 10 | 4.98#（2.39-9.16） | 1 | 1.57（0.04-8.77） | 0 | 0（0-30.42） |
| Diabetes Mellitus | 10 | 1.93（0.93-3.56） | 3 | 1.88（0.39-5.49） | 4 | 1.55（0.42-3.96） | 2 | 2.41（0.29-8.7） | 1 | 6.29（0.16-35.06） |
| Alzheimers (ICD-9 and 10 only) | 4 | 0.87（0.24-2.23） | 2 | 1.66（0.2-5.99） | 1 | 0.46（0.01-2.54） | 1 | 1.03（0.03-5.74） | 0 | 0（0-15.96） |
| Diseases of Heart | 72 | 1.77#（1.39-2.23） | 33 | 2.64#（1.82-3.71） | 26 | 1.3（0.85-1.9） | 10 | 1.48（0.71-2.71） | 3 | 2.31（0.48-6.74） |
| Hypertension without Heart Disease | 4 | 2.38（0.65-6.1） | 1 | 2.06（0.05-11.47） | 3 | 3.63（0.75-10.62） | 0 | 0（0-12.17） | 0 | 0（0-58） |
| Cerebrovascular Diseases | 18 | 2.26#（1.34-3.57） | 3 | 1.24（0.25-3.61） | 10 | 2.57#（1.23-4.72） | 5 | 3.66#（1.19-8.53） | 0 | 0（0-12.93） |
| Diseases of Arteries | 4 | 2.27（0.62-5.81） | 1 | 1.78（0.04-9.9） | 3 | 3.45（0.71-10.08） | 0 | 0（0-13.32） | 0 | 0（0-71.87） |
| Pneumonia and Influenza | 25 | 1.86#（1.2-2.74） | 13 | 3.22#（1.71-5.5） | 10 | 1.5（0.72-2.77） | 2 | 0.86（0.1-3.12） | 0 | 0（0-8.02） |
| Digestive | 4 | 1.53（0.42-3.92） | 1 | 1.2（0.03-6.66） | 3 | 2.26（0.47-6.59） | 0 | 0（0-9.66） | 0 | 0（0-54.88） |
| Nephritis\ Nephrotic Syndrome\Nephrosis | 14 | 4.54#（2.48-7.62） | 5 | 5.37#（1.75-12.54） | 9 | 5.90#（2.7-11.2） | 0 | 0（0-7.03） | 0 | 0（0-36.12） |
| Pregnancy\Childbirth\Puerperium | 3 | 16.34#（3.37-47.76） | 1 | 16.78（0.42-93.47） | 2 | 21.60#（2.62-78.02） | 0 | 0（0-140.38） | 0 | 0（0-726.72） |
| Symptoms, Signs and Ill-Defined Conditions | 4 | 2.46（0.67-6.3） | 1 | 2（0.05-11.14） | 2 | 2.48（0.3-8.97） | 1 | 3.69（0.09-20.55） | 0 | 0（0-74） |
| Accidents\Adverse Effects\Homicide\Legal intervention | 10 | 1.71（0.82-3.15） | 3 | 1.64（0.34-4.79） | 6 | 2.06（0.75-4.47） | 0 | 0（0-4.07） | 1 | 5.61（0.14-31.27） |
| Suicide\Self-Inflicted Injury | 5 | 2.85（0.92-6.65） | 2 | 3.5（0.42-12.64） | 3 | 3.33（0.69-9.75） | 0 | 0（0-15.05） | 0 | 0（0-93.72） |
| Other Cause of Death | 56 | 2.39#（1.8-3.1） | 27 | 4.01#（2.64-5.84） | 21 | 1.82#（1.13-2.78） | 6 | 1.4（0.51-3.05） | 2 | 2.26（0.27-8.18） |

Supplementary Table 14. Standardized-mortality ratios (SMRs) for each cause of death following RCC diagnosis in patients without radiation.

|  | Timing of Death After Diagnosis | | | | | | | | | |
| --- | --- | --- | --- | --- | --- | --- | --- | --- | --- | --- |
|  | All Y ears | | <1 y | | 1 to <5 y | | 5 to <10 y | | ≥10 y | |
| All Causes of Death | 35,049 | 2.38#（2.35-2.4） | 9,076 | 5.90#（5.78-6.02） | 15,118 | 2.26#（2.22-2.29） | 8,594 | 1.69#（1.66-1.73） | 2,261 | 1.58#（1.51-1.64） |
| All Malignant Cancers | 20,787 | 5.81#（5.73-5.89） | 7,173 | 17.87#（17.46-18.29） | 9,192 | 5.47#（5.36-5.58） | 3,568 | 3.01#（2.91-3.11） | 854 | 2.75#（2.57-2.94） |
| Oral Cavity/Pharynx/eye/endocrine | 69 | 0.92（0.71-1.16） | 15 | 1.81#（1.01-2.99） | 26 | 0.74（0.48-1.08） | 19 | 0.76（0.46-1.19） | 9 | 1.36（0.62-2.58） |
| Digestive System | 1,033 | 1.11#（1.04-1.18） | 144 | 1.40#（1.18-1.64） | 425 | 0.97（0.88-1.07） | 351 | 1.14#（1.02-1.26） | 113 | 1.39#（1.14-1.67） |
| Respiratory System | 1,153 | 1.12#（1.06-1.19） | 174 | 1.43#（1.23-1.66） | 504 | 1.02（0.94-1.12） | 372 | 1.12#（1.01-1.24） | 103 | 1.25#（1.02-1.52） |
| Bones and Joints/soft tissue/skin | 113 | 1.05（0.87-1.26） | 31 | 2.64#（1.8-3.75） | 39 | 0.78（0.55-1.07） | 32 | 0.89（0.61-1.25） | 11 | 1.14（0.57-2.04） |
| Breast | 69 | 0.44#（0.34-0.55） | 5 | 0.28#（0.09-0.64） | 17 | 0.23#（0.13-0.36） | 32 | 0.61#（0.42-0.87） | 15 | 1.12（0.63-1.85） |
| Genital System | 229 | 0.62#（0.54-0.71） | 28 | 0.72（0.48-1.04） | 99 | 0.59#（0.48-0.72） | 73 | 0.58#（0.46-0.73） | 29 | 0.83（0.56-1.19） |
| Kidney and Renal Pelvis | 16,486 | 185.95#（183.12-188.81） | 6,330 | 638.83#（623.19-654.76） | 7,397 | 177.54#（173.52-181.63） | 2,294 | 78.08#（74.92-81.34） | 465 | 60.34#（54.98-66.08） |
| Other Urinary Organs | 208 | 1.77#（1.54-2.03） | 54 | 4.54#（3.41-5.92） | 104 | 1.97#（1.61-2.38） | 43 | 1.05（0.76-1.41） | 7 | 0.6（0.24-1.23） |
| Brain/Other Nervous System | 134 | 1.65#（1.38-1.95） | 14 | 1.53（0.84-2.57） | 66 | 1.71#（1.32-2.18） | 44 | 1.65#（1.2-2.21） | 10 | 1.44（0.69-2.65） |
| lymphandblood | 426 | 1.20#（1.09-1.32） | 82 | 2.12#（1.69-2.63） | 179 | 1.09（0.94-1.26） | 128 | 1.07（0.89-1.27） | 37 | 1.15（0.81-1.59） |
| Miscellaneous Malignant Cancer | 867 | 3.21#（3-3.43） | 296 | 9.89#（8.79-11.08） | 336 | 2.67#（2.39-2.97） | 180 | 2.00#（1.72-2.31） | 55 | 2.28#（1.72-2.97） |
| In situ, benign or unknown behavior neoplasm | 160 | 1.69#（1.44-1.98） | 28 | 2.91#（1.94-4.21） | 69 | 1.62#（1.26-2.05） | 52 | 1.58#（1.18-2.07） | 11 | 1.18（0.59-2.11） |
| Noncancer | 14,102 | 1.27#（1.25-1.29） | 1,875 | 1.66#（1.59-1.74） | 5,857 | 1.18#（1.15-1.21） | 4,974 | 1.29#（1.25-1.33） | 1,396 | 1.25#（1.19-1.32） |
| infections | 582 | 1.60#（1.47-1.74） | 104 | 2.60#（2.12-3.15） | 240 | 1.42#（1.24-1.61） | 188 | 1.55#（1.33-1.78） | 50 | 1.53#（1.14-2.02） |
| Diabetes Mellitus | 898 | 1.94#（1.81-2.07） | 123 | 2.45#（2.04-2.93） | 388 | 1.81#（1.63-1.99） | 307 | 1.96#（1.75-2.2） | 80 | 1.88#（1.49-2.34） |
| Alzheimers (ICD-9 and 10 only) | 441 | 0.83#（0.76-0.91） | 26 | 0.61#（0.4-0.89） | 151 | 0.70#（0.59-0.82） | 196 | 0.97（0.84-1.11） | 68 | 0.98（0.76-1.24） |
| Diseases of Heart | 4,682 | 1.26#（1.22-1.29） | 644 | 1.64#（1.52-1.78） | 1,964 | 1.16#（1.11-1.21） | 1,624 | 1.27#（1.21-1.33） | 450 | 1.23#（1.12-1.35） |
| Hypertension without Heart Disease | 379 | 2.19#（1.98-2.42） | 41 | 2.51#（1.8-3.41） | 144 | 1.90#（1.6-2.24） | 147 | 2.36#（2-2.78） | 47 | 2.52#（1.85-3.35） |
| Cerebrovascular Diseases | 925 | 1.18#（1.1-1.25） | 123 | 1.53#（1.27-1.83） | 412 | 1.17#（1.06-1.29） | 296 | 1.08（0.96-1.21） | 94 | 1.16（0.94-1.42） |
| Diseases of Arteries | 190 | 1.20#（1.04-1.39） | 32 | 1.80#（1.23-2.54） | 75 | 1.02（0.8-1.28） | 68 | 1.30#（1.01-1.64） | 15 | 1.05（0.59-1.74） |
| Pneumonia and Influenza | 1,311 | 1.05（0.99-1.11） | 151 | 1.18（1-1.38） | 540 | 0.96（0.88-1.04） | 505 | 1.17#（1.07-1.27） | 115 | 0.94（0.77-1.13） |
| Digestive | 290 | 1.44#（1.28-1.62） | 39 | 1.68#（1.2-2.3） | 139 | 1.44#（1.21-1.7） | 93 | 1.44#（1.16-1.76） | 19 | 1.17（0.7-1.82） |
| Nephritis\ Nephrotic Syndrome\Nephrosis | 845 | 2.84#（2.65-3.04） | 101 | 3.31#（2.69-4.02） | 339 | 2.52#（2.26-2.8） | 306 | 2.96#（2.64-3.31） | 99 | 3.39#（2.75-4.12） |
| Pregnancy\Childbirth\Puerperium | 39 | 2.60#（1.85-3.56） | 5 | 2.84（0.92-6.63） | 17 | 2.35#（1.37-3.77） | 13 | 2.72#（1.45-4.64） | 4 | 3.31（0.9-8.47） |
| Symptoms, Signs and Ill-Defined Conditions | 214 | 1.35#（1.18-1.55） | 41 | 2.51#（1.8-3.41） | 95 | 1.32#（1.07-1.61） | 68 | 1.22（0.95-1.55） | 10 | 0.69（0.33-1.27） |
| Accidents\Adverse Effects\Homicide\Legal intervention | 572 | 1.13#（1.04-1.23） | 71 | 1.30#（1.01-1.63） | 265 | 1.13（1-1.27） | 183 | 1.08（0.93-1.25） | 53 | 1.14（0.85-1.49） |
| Suicide\Self-Inflicted Injury | 135 | 1.05（0.88-1.25） | 19 | 1.23（0.74-1.91） | 66 | 1.05（0.81-1.34） | 41 | 1.02（0.73-1.38） | 9 | 0.93（0.42-1.76） |
| Other Cause of Death | 2,599 | 1.12#（1.08-1.17） | 355 | 1.63#（1.46-1.81） | 1,022 | 1.01（0.95-1.07） | 939 | 1.12#（1.05-1.2） | 283 | 1.14#（1.01-1.28） |

Supplementary Table 15. Standardized-mortality ratios (SMRs) for each cause of death following RCC diagnosis in patients with chemotherapy.

|  | Timing of Death After Diagnosis | | | | | | | | | |
| --- | --- | --- | --- | --- | --- | --- | --- | --- | --- | --- |
|  | All Y ears | | <1 y | | 1 to <5 y | | 5 to <10 y | | ≥10 y | |
| All Causes of Death | 7,976 | 20.49#（20.04-20.94） | 4,018 | 39.73#（38.51-40.97） | 3,521 | 17.96#（17.37-18.56） | 390 | 5.02#（4.54-5.55） | 47 | 3.25#（2.39-4.33） |
| All Malignant Cancers | 7,442 | 71.85#（70.22-73.5） | 3,811 | 135.47#（131.2-139.84） | 3,278 | 61.62#（59.53-63.76） | 325 | 17.07#（15.27-19.04） | 28 | 8.71#（5.79-12.58） |
| Oral Cavity/Pharynx/eye/endocrine | 5 | 2.15（0.7-5.03） | 3 | 4.84（1-14.15） | 2 | 1.66（0.2-6） | 0 | 0（0-8.65） | 0 | 0（0-52.53） |
| Digestive System | 54 | 1.97#（1.48-2.57） | 25 | 3.39#（2.2-5.01） | 17 | 1.2（0.7-1.92） | 11 | 2.16#（1.08-3.87） | 1 | 1.17（0.03-6.54） |
| Respiratory System | 83 | 2.68#（2.14-3.33） | 39 | 4.48#（3.19-6.12） | 36 | 2.25#（1.58-3.12） | 6 | 1.11（0.41-2.42） | 2 | 2.35（0.28-8.48） |
| Bones and Joints/soft tissue/skin | 12 | 3.71#（1.92-6.49） | 8 | 9.27#（4-18.27） | 4 | 2.4（0.65-6.13） | 0 | 0（0-6.17） | 0 | 0（0-36.97） |
| Breast | 1 | 0.28（0.01-1.54） | 0 | 0（0-3.64） | 1 | 0.55（0.01-3.09） | 0 | 0（0-5.39） | 0 | 0（0-30.2） |
| Genital System | 19 | 1.95#（1.18-3.05） | 8 | 3.15#（1.36-6.21） | 9 | 1.83（0.84-3.48） | 1 | 0.52（0.01-2.91） | 1 | 2.73（0.07-15.2） |
| Kidney and Renal Pelvis | 6,883 | 2,551.17#（2491.26-2612.17） | 3,536 | 4,842.52#（4684.21-5004.82） | 3,049 | 2,183.23#（2106.41-2262.12） | 277 | 564.36#（499.85-634.88） | 21 | 261.21#（161.7-399.29） |
| Other Urinary Organs | 51 | 15.49#（11.53-20.37） | 22 | 26.29#（16.47-39.8） | 27 | 16.14#（10.63-23.48） | 2 | 3.03（0.37-10.96） | 0 | 0（0-29.99） |
| Brain/Other Nervous System | 8 | 3.13#（1.35-6.17） | 1 | 1.45（0.04-8.09） | 5 | 3.75#（1.22-8.74） | 2 | 4.36（0.53-15.75） | 0 | 0（0-50.58） |
| lymphandblood | 110 | 10.97#（9.02-13.22） | 49 | 18.29#（13.53-24.18） | 45 | 8.78#（6.41-11.75） | 14 | 7.39#（4.04-12.39） | 2 | 6.07（0.74-21.94） |
| Miscellaneous Malignant Cancer | 216 | 27.95#（24.35-31.94） | 120 | 57.53#（47.7-68.79） | 83 | 20.97#（16.71-26） | 12 | 8.35#（4.31-14.58） | 1 | 4.04（0.1-22.54） |
| In situ, benign or unknown behavior neoplasm | 16 | 6.43#（3.67-10.44） | 11 | 17.31#（8.64-30.96） | 5 | 3.99#（1.3-9.31） | 0 | 0（0-7.29） | 0 | 0（0-39.01） |
| Noncancer | 518 | 1.83#（1.67-1.99） | 196 | 2.71#（2.34-3.12） | 238 | 1.68#（1.47-1.91） | 65 | 1.12（0.86-1.43） | 19 | 1.71#（1.03-2.66） |
| infections | 39 | 3.93#（2.79-5.37） | 18 | 6.72#（3.98-10.62） | 15 | 2.98#（1.67-4.91） | 6 | 3.21#（1.18-6.98） | 0 | 0（0-11.09） |
| Diabetes Mellitus | 26 | 2.02#（1.32-2.96） | 7 | 2.06（0.83-4.25） | 10 | 1.52（0.73-2.8） | 8 | 3.22#（1.39-6.35） | 1 | 2.23（0.06-12.45） |
| Alzheimers (ICD-9 and 10 only) | 4 | 0.38#（0.1-0.98） | 0 | 0（0-1.64） | 2 | 0.41（0.05-1.5） | 1 | 0.37（0.01-2.06） | 1 | 1.56（0.04-8.68） |
| Diseases of Heart | 151 | 1.56#（1.32-1.83） | 62 | 2.46#（1.89-3.16） | 63 | 1.30#（1-1.67） | 20 | 1.03（0.63-1.59） | 6 | 1.63（0.6-3.55） |
| Hypertension without Heart Disease | 6 | 1.46（0.54-3.19） | 2 | 2.03（0.25-7.32） | 3 | 1.5（0.31-4.37） | 1 | 1.09（0.03-6.1） | 0 | 0（0-19.45） |
| Cerebrovascular Diseases | 40 | 2.11#（1.51-2.88） | 14 | 2.89#（1.58-4.85） | 21 | 2.25#（1.4-3.45） | 3 | 0.75（0.16-2.2） | 2 | 2.5（0.3-9.02） |
| Diseases of Arteries | 8 | 1.94（0.84-3.83） | 2 | 1.79（0.22-6.45） | 6 | 2.90#（1.07-6.32） | 0 | 0（0-4.69） | 0 | 0（0-26.06） |
| Pneumonia and Influenza | 38 | 1.17（0.83-1.6） | 14 | 1.67（0.92-2.81） | 19 | 1.16（0.7-1.81） | 4 | 0.61（0.17-1.55） | 1 | 0.81（0.02-4.52） |
| Digestive | 6 | 0.91（0.33-1.98） | 0 | 0（0-2.06） | 6 | 1.73（0.64-3.77） | 0 | 0（0-3.22） | 0 | 0（0-21.07） |
| Nephritis\ Nephrotic Syndrome\Nephrosis | 27 | 3.61#（2.38-5.25） | 9 | 4.70#（2.15-8.91） | 11 | 2.94#（1.47-5.27） | 3 | 1.95（0.4-5.71） | 4 | 13.30#（3.62-34.06） |
| Pregnancy\Childbirth\Puerperium | 6 | 12.90#（4.73-28.07） | 3 | 23.25#（4.79-67.95） | 3 | 12.37#（2.55-36.16） | 0 | 0（0-45.73） | 0 | 0（0-281.67） |
| Symptoms, Signs and Ill-Defined Conditions | 21 | 5.56#（3.44-8.5） | 11 | 11.35#（5.67-20.32） | 9 | 4.80#（2.19-9.11） | 1 | 1.26（0.03-7.02） | 0 | 0（0-26.71） |
| Accidents\Adverse Effects\Homicide\Legal intervention | 26 | 1.79#（1.17-2.63） | 3 | 0.78（0.16-2.28） | 15 | 2.02#（1.13-3.32） | 6 | 2.21（0.81-4.82） | 2 | 4.06（0.49-14.67） |
| Suicide\Self-Inflicted Injury | 12 | 2.71#（1.4-4.74） | 9 | 7.36#（3.37-13.97） | 3 | 1.28（0.26-3.73） | 0 | 0（0-4.99） | 0 | 0（0-33.51） |
| Other Cause of Death | 108 | 1.91#（1.57-2.31） | 42 | 3.06#（2.21-4.14） | 52 | 1.86#（1.39-2.44） | 12 | 0.97（0.5-1.7） | 2 | 0.82（0.1-2.96） |

Supplementary Table 16. Standardized-mortality ratios (SMRs) for each cause of death following RCC diagnosis in patients without chemotherapy.

|  | Timing of Death After Diagnosis | | | | | | | | | |
| --- | --- | --- | --- | --- | --- | --- | --- | --- | --- | --- |
|  | All Y ears | | <1 y | | 1 to <5 y | | 5 to <10 y | | ≥10 y | |
| All Causes of Death | 31,654 | 2.18#（2.16-2.2） | 7,724 | 5.20#（5.08-5.31） | 13,310 | 2.02#（1.99-2.06） | 8,380 | 1.67#（1.63-1.7） | 2,240 | 1.57#（1.51-1.64） |
| All Malignant Cancers | 17,654 | 5.02#（4.95-5.09） | 5,909 | 15.29#（14.91-15.69） | 7,509 | 4.55#（4.45-4.66） | 3,391 | 2.89#（2.79-2.99） | 845 | 2.74#（2.55-2.93） |
| Oral Cavity/Pharynx/eye/endocrine | 66 | 0.9（0.69-1.14） | 13 | 1.63（0.87-2.8） | 25 | 0.72（0.47-1.07） | 19 | 0.77（0.46-1.2） | 9 | 1.37（0.62-2.59） |
| Digestive System | 1,008 | 1.10#（1.04-1.17） | 132 | 1.33#（1.11-1.58） | 417 | 0.97（0.88-1.07） | 345 | 1.13#（1.01-1.25） | 114 | 1.41#（1.16-1.69） |
| Respiratory System | 1,122 | 1.11#（1.05-1.18） | 165 | 1.41#（1.2-1.64） | 484 | 1（0.91-1.1） | 371 | 1.13#（1.02-1.25） | 102 | 1.25#（1.02-1.51） |
| Bones and Joints/soft tissue/skin | 114 | 1.08（0.89-1.3） | 29 | 2.57#（1.72-3.7） | 42 | 0.86（0.62-1.16） | 32 | 0.9（0.61-1.26） | 11 | 1.15（0.57-2.05） |
| Breast | 70 | 0.45#（0.35-0.57） | 6 | 0.34#（0.12-0.74） | 17 | 0.23#（0.13-0.37） | 32 | 0.62#（0.42-0.88） | 15 | 1.13（0.63-1.86） |
| Genital System | 232 | 0.64#（0.56-0.73） | 34 | 0.9（0.63-1.26） | 97 | 0.59#（0.48-0.72） | 73 | 0.59#（0.46-0.74） | 28 | 0.81（0.54-1.17） |
| Kidney and Renal Pelvis | 13,612 | 156.36#（153.74-159.01） | 5,162 | 542.28#（527.59-557.28） | 5,849 | 143.29#（139.64-147.01） | 2,144 | 73.77#（70.68-76.97） | 457 | 59.69#（54.34-65.42） |
| Other Urinary Organs | 179 | 1.55#（1.33-1.79） | 46 | 4.01#（2.94-5.35） | 85 | 1.64#（1.31-2.03） | 41 | 1.01（0.72-1.37） | 7 | 0.6（0.24-1.23） |
| Brain/Other Nervous System | 133 | 1.66#（1.39-1.97） | 17 | 1.94#（1.13-3.1） | 62 | 1.64#（1.26-2.1） | 44 | 1.66#（1.21-2.23） | 10 | 1.45（0.7-2.67） |
| lymphandblood | 330 | 0.95（0.85-1.06） | 38 | 1.02（0.72-1.4） | 140 | 0.87（0.73-1.03） | 116 | 0.98（0.81-1.18） | 36 | 1.13（0.79-1.56） |
| Miscellaneous Malignant Cancer | 788 | 2.97#（2.77-3.18） | 267 | 9.26#（8.18-10.44） | 291 | 2.36#（2.09-2.64） | 174 | 1.95#（1.67-2.27） | 56 | 2.34#（1.76-3.03） |
| In situ, benign or unknown behavior neoplasm | 159 | 1.71#（1.45-2） | 27 | 2.91#（1.92-4.23） | 69 | 1.65#（1.29-2.09） | 52 | 1.59#（1.19-2.09） | 11 | 1.18（0.59-2.12） |
| Noncancer | 13,841 | 1.27#（1.25-1.29） | 1,788 | 1.64#（1.56-1.72） | 5,732 | 1.17#（1.14-1.2） | 4,937 | 1.29#（1.26-1.33） | 1,384 | 1.25#（1.19-1.32） |
| infections | 567 | 1.59#（1.46-1.72） | 99 | 2.56#（2.08-3.12） | 235 | 1.41#（1.24-1.61） | 183 | 1.52#（1.31-1.76） | 50 | 1.54#（1.14-2.03） |
| Diabetes Mellitus | 882 | 1.93#（1.81-2.07） | 119 | 2.46#（2.04-2.94） | 382 | 1.81#（1.63-2） | 301 | 1.95#（1.73-2.18） | 80 | 1.89#（1.5-2.36） |
| Alzheimers (ICD-9 and 10 only) | 441 | 0.84#（0.76-0.92） | 28 | 0.67#（0.45-0.97） | 150 | 0.70#（0.6-0.83） | 196 | 0.98（0.84-1.12） | 67 | 0.97（0.75-1.23） |
| Diseases of Heart | 4,603 | 1.25#（1.22-1.29） | 615 | 1.62#（1.5-1.76） | 1,927 | 1.16#（1.11-1.21） | 1,614 | 1.27#（1.21-1.34） | 447 | 1.23#（1.12-1.35） |
| Hypertension without Heart Disease | 377 | 2.21#（1.99-2.45） | 40 | 2.53#（1.81-3.45） | 144 | 1.93#（1.63-2.27） | 146 | 2.37#（2-2.79） | 47 | 2.53#（1.86-3.37） |
| Cerebrovascular Diseases | 903 | 1.16#（1.09-1.24） | 112 | 1.44#（1.18-1.73） | 401 | 1.16#（1.05-1.28） | 298 | 1.1（0.98-1.23） | 92 | 1.14（0.92-1.4） |
| Diseases of Arteries | 186 | 1.20#（1.03-1.38） | 31 | 1.80#（1.22-2.55） | 72 | 1（0.78-1.26） | 68 | 1.31#（1.02-1.66） | 15 | 1.06（0.59-1.75） |
| Pneumonia and Influenza | 1,298 | 1.06#（1-1.12） | 150 | 1.21#（1.03-1.42） | 531 | 0.96（0.88-1.04） | 503 | 1.17#（1.07-1.28） | 114 | 0.94（0.77-1.12） |
| Digestive | 288 | 1.46#（1.3-1.64） | 40 | 1.80#（1.29-2.45） | 136 | 1.44#（1.21-1.7） | 93 | 1.45#（1.17-1.78） | 19 | 1.17（0.71-1.83） |
| Nephritis\ Nephrotic Syndrome\Nephrosis | 832 | 2.84#（2.65-3.04） | 97 | 3.28#（2.66-4） | 337 | 2.55#（2.28-2.83） | 303 | 2.96#（2.64-3.32） | 95 | 3.27#（2.65-4） |
| Pregnancy\Childbirth\Puerperium | 36 | 2.45#（1.72-3.39） | 3 | 1.77（0.37-5.18） | 16 | 2.26#（1.29-3.67） | 13 | 2.75#（1.46-4.7） | 4 | 3.33（0.91-8.52） |
| Symptoms, Signs and Ill-Defined Conditions | 197 | 1.26#（1.09-1.45） | 31 | 1.95#（1.33-2.77） | 88 | 1.24（1-1.53） | 68 | 1.23（0.96-1.57） | 10 | 0.7（0.33-1.28） |
| Accidents\Adverse Effects\Homicide\Legal intervention | 556 | 1.12#（1.03-1.22） | 71 | 1.35#（1.05-1.7） | 256 | 1.11（0.98-1.25） | 177 | 1.06（0.91-1.22） | 52 | 1.13（0.84-1.48） |
| Suicide\Self-Inflicted Injury | 128 | 1.02（0.85-1.21） | 12 | 0.81（0.42-1.41） | 66 | 1.07（0.83-1.37） | 41 | 1.03（0.74-1.4） | 9 | 0.93（0.43-1.77） |
| Other Cause of Death | 2,547 | 1.12#（1.07-1.16） | 340 | 1.61#（1.45-1.79） | 991 | 0.99（0.93-1.06） | 933 | 1.13#（1.05-1.2） | 283 | 1.15#（1.02-1.29） |

Supplementary Table 17. Standardized-mortality ratios (SMRs) for each cause of death following RCC diagnosis in patients diagnosis in 2004-2007.

|  | Timing of Death After Diagnosis | | | | | | | | | |
| --- | --- | --- | --- | --- | --- | --- | --- | --- | --- | --- |
|  | All Y ears | | <1 y | | 1 to <5 y | | 5 to <10 y | | ≥10 y | |
| All Causes of Death | 15,454 | 2.38#(2.34-2.42) | 3,745 | 7.83#(7.58-8.09) | 5,221 | 2.46#(2.39-2.53) | 4,302 | 1.71#(1.65-1.76) | 2,186 | 1.60#(1.53-1.67) |
| All Malignant Cancers | 9,251 | 5.94#(5.82-6.06) | 3,129 | 24.84#(23.98-25.73) | 3,428 | 6.32#(6.11-6.54) | 1,867 | 3.15#(3.01-3.29) | 827 | 2.79#(2.6-2.99) |
| Oral Cavity/Pharynx/eye/endocrine | 31 | 1(0.68-1.42) | 7 | 2.93#(1.18-6.04) | 7 | 0.68(0.27-1.39) | 8 | 0.67(0.29-1.32) | 9 | 1.42(0.65-2.7) |
| Digestive System | 486 | 1.23#(1.12-1.34) | 52 | 1.67#(1.25-2.19) | 141 | 1.04(0.88-1.23) | 185 | 1.22#(1.05-1.41) | 108 | 1.39#(1.14-1.68) |
| Respiratory System | 531 | 1.17#(1.07-1.27) | 66 | 1.66#(1.28-2.11) | 177 | 1.06(0.91-1.23) | 187 | 1.1(0.95-1.27) | 101 | 1.29#(1.05-1.56) |
| Bones and Joints/soft tissue/skin | 53 | 1.15(0.86-1.5) | 12 | 3.46#(1.79-6.05) | 17 | 1.1(0.64-1.77) | 14 | 0.78(0.42-1.3) | 10 | 1.09(0.52-2) |
| Breast | 38 | 0.55#(0.39-0.76) | 0 | 0.00#(0-0.64) | 10 | 0.41#(0.2-0.76) | 14 | 0.54#(0.29-0.9) | 14 | 1.1(0.6-1.84) |
| Genital System | 112 | 0.69#(0.57-0.83) | 15 | 1.2(0.67-1.98) | 31 | 0.57#(0.38-0.8) | 39 | 0.63#(0.45-0.86) | 27 | 0.81(0.53-1.18) |
| Kidney and Renal Pelvis | 7,299 | 191.89#(187.52-196.35) | 2,811 | 933.69#(899.49-968.86) | 2,810 | 215.09#(207.21-223.2) | 1,226 | 83.90#(79.27-88.73) | 452 | 61.50#(55.96-67.44) |
| Other Urinary Organs | 71 | 1.37#(1.07-1.73) | 19 | 5.28#(3.18-8.25) | 33 | 2.00#(1.37-2.8) | 12 | 0.59(0.3-1.02) | 7 | 0.62(0.25-1.28) |
| Brain/Other Nervous System | 63 | 1.89#(1.45-2.42) | 9 | 3.42#(1.56-6.5) | 22 | 1.94#(1.22-2.94) | 23 | 1.80#(1.14-2.69) | 9 | 1.36(0.62-2.58) |
| lymphandblood | 180 | 1.15(0.99-1.33) | 28 | 2.30#(1.53-3.32) | 59 | 1.11(0.84-1.43) | 57 | 0.95(0.72-1.23) | 36 | 1.17(0.82-1.63) |
| Miscellaneous Malignant Cancer | 387 | 3.27#(2.95-3.61) | 110 | 11.53#(9.47-13.89) | 121 | 2.96#(2.45-3.53) | 102 | 2.27#(1.85-2.76) | 54 | 2.35#(1.76-3.06) |
| In situ, benign or unknown behavior neoplasm | 75 | 1.78#(1.4-2.23) | 12 | 4.05#(2.09-7.07) | 26 | 1.92#(1.25-2.81) | 26 | 1.56#(1.02-2.29) | 11 | 1.23(0.62-2.21) |
| Noncancer | 6,128 | 1.25#(1.22-1.28) | 604 | 1.73#(1.59-1.87) | 1,767 | 1.13#(1.07-1.18) | 2,409 | 1.26#(1.21-1.31) | 1,348 | 1.27#(1.2-1.34) |
| infections | 261 | 1.66#(1.46-1.87) | 30 | 2.45#(1.65-3.5) | 84 | 1.57#(1.25-1.94) | 99 | 1.64#(1.33-1.99) | 48 | 1.54#(1.14-2.05) |
| Diabetes Mellitus | 392 | 1.97#(1.78-2.17) | 40 | 2.58#(1.85-3.52) | 118 | 1.76#(1.45-2.1) | 156 | 2.05#(1.74-2.4) | 78 | 1.93#(1.52-2.4) |
| Alzheimers (ICD-9 and 10 only) | 206 | 0.86#(0.75-0.99) | 6 | 0.48(0.18-1.04) | 40 | 0.63#(0.45-0.86) | 94 | 0.98(0.79-1.2) | 66 | 0.99(0.77-1.26) |
| Diseases of Heart | 2,049 | 1.22#(1.17-1.27) | 237 | 1.84#(1.61-2.09) | 561 | 1(0.92-1.09) | 810 | 1.26#(1.17-1.35) | 441 | 1.26#(1.15-1.39) |
| Hypertension without Heart Disease | 153 | 2.05#(1.74-2.4) | 10 | 2.16#(1.04-3.98) | 35 | 1.58#(1.1-2.2) | 63 | 2.09#(1.61-2.68) | 45 | 2.52#(1.84-3.38) |
| Cerebrovascular Diseases | 419 | 1.17#(1.06-1.29) | 44 | 1.64#(1.19-2.21) | 141 | 1.21#(1.02-1.43) | 149 | 1.09(0.92-1.28) | 85 | 1.1(0.88-1.36) |
| Diseases of Arteries | 81 | 1.09(0.87-1.36) | 8 | 1.24(0.53-2.44) | 26 | 0.97(0.63-1.42) | 32 | 1.17(0.8-1.66) | 15 | 1.1(0.62-1.82) |
| Pneumonia and Influenza | 583 | 1.05(0.96-1.14) | 48 | 1.19(0.88-1.57) | 179 | 0.98(0.85-1.14) | 247 | 1.14#(1-1.29) | 109 | 0.93(0.76-1.12) |
| Digestive | 115 | 1.45#(1.2-1.75) | 11 | 1.73(0.86-3.09) | 44 | 1.63#(1.19-2.19) | 42 | 1.39#(1-1.88) | 18 | 1.16(0.69-1.83) |
| Nephritis\ Nephrotic Syndrome\Nephrosis | 368 | 2.77#(2.49-3.07) | 34 | 3.54#(2.45-4.95) | 104 | 2.38#(1.95-2.89) | 136 | 2.63#(2.2-3.11) | 94 | 3.37#(2.72-4.13) |
| Pregnancy\Childbirth\Puerperium | 12 | 2.01#(1.04-3.51) | 1 | 1.97(0.05-10.99) | 2 | 0.96(0.12-3.47) | 5 | 2.24(0.73-5.22) | 4 | 3.47(0.95-8.88) |
| Symptoms, Signs and Ill-Defined Conditions | 105 | 1.45#(1.18-1.75) | 15 | 3.06#(1.71-5.05) | 42 | 1.79#(1.29-2.41) | 38 | 1.25(0.89-1.72) | 10 | 0.72(0.35-1.33) |
| Accidents\Adverse Effects\Homicide\Legal intervention | 227 | 1.1(0.96-1.26) | 20 | 1.31(0.8-2.02) | 73 | 1.1(0.86-1.38) | 81 | 1.02(0.81-1.27) | 53 | 1.19(0.89-1.56) |
| Suicide\Self-Inflicted Injury | 52 | 1.04(0.78-1.37) | 7 | 1.67(0.67-3.44) | 17 | 0.97(0.56-1.55) | 19 | 1(0.6-1.57) | 9 | 0.97(0.44-1.84) |
| Other Cause of Death | 1,105 | 1.10#(1.03-1.16) | 93 | 1.53#(1.23-1.87) | 301 | 1.02(0.91-1.14) | 438 | 1.06(0.96-1.17) | 273 | 1.15#(1.02-1.29) |

Supplementary Table 18. Standardized-mortality ratios (SMRs) for each cause of death following RCC diagnosis in patients diagnosis in 2008-2011.

|  | Timing of Death After Diagnosis | | | | | | | | | |
| --- | --- | --- | --- | --- | --- | --- | --- | --- | --- | --- |
|  | All Y ears | | <1 y | | 1 to <5 y | | 5 to <10 y | | ≥10 y | |
| All Causes of Death | 13,787 | 2.62#(2.58-2.66) | 3,930 | 7.41#(7.18-7.65) | 5,818 | 2.44#(2.38-2.5) | 3,938 | 1.73#(1.68-1.78) | 101 | 1.45#(1.18-1.76) |
| All Malignant Cancers | 8,638 | 6.72#(6.58-6.86) | 3,245 | 23.30#(22.5-24.11) | 3,717 | 6.16#(5.96-6.36) | 1,630 | 3.09#(2.95-3.25) | 46 | 2.94#(2.15-3.92) |
| Oral Cavity/Pharynx/eye/endocrine | 27 | 0.99(0.65-1.44) | 4 | 1.4(0.38-3.59) | 12 | 0.95(0.49-1.66) | 11 | 0.95(0.48-1.71) | 0 | 0(0-10.91) |
| Digestive System | 351 | 1.04(0.94-1.16) | 49 | 1.38#(1.02-1.82) | 139 | 0.89(0.75-1.05) | 156 | 1.12(0.95-1.3) | 7 | 1.69(0.68-3.48) |
| Respiratory System | 404 | 1.1(0.99-1.21) | 69 | 1.62#(1.26-2.05) | 166 | 0.93(0.8-1.08) | 166 | 1.16(0.99-1.35) | 3 | 0.72(0.15-2.1) |
| Bones and Joints/soft tissue/skin | 40 | 1.03(0.73-1.4) | 9 | 2.19#(1-4.16) | 14 | 0.77(0.42-1.29) | 16 | 1(0.57-1.62) | 1 | 2.1(0.05-11.69) |
| Breast | 24 | 0.42#(0.27-0.62) | 3 | 0.47(0.1-1.38) | 3 | 0.11#(0.02-0.32) | 17 | 0.73(0.43-1.18) | 1 | 1.5(0.04-8.38) |
| Genital System | 82 | 0.63#(0.5-0.78) | 10 | 0.75(0.36-1.38) | 37 | 0.63#(0.44-0.86) | 33 | 0.59#(0.4-0.82) | 2 | 1.14(0.14-4.13) |
| Kidney and Renal Pelvis | 7,052 | 220.14#(215.03-225.33) | 2,902 | 841.27#(810.94-872.45) | 3,079 | 204.49#(197.33-211.85) | 1,045 | 79.52#(74.78-84.5) | 26 | 67.03#(43.79-98.21) |
| Other Urinary Organs | 88 | 2.10#(1.68-2.58) | 21 | 5.11#(3.16-7.81) | 39 | 2.06#(1.46-2.82) | 28 | 1.53#(1.01-2.2) | 0 | 0(0-6.39) |
| Brain/Other Nervous System | 50 | 1.68#(1.25-2.22) | 4 | 1.27(0.34-3.24) | 23 | 1.66#(1.05-2.49) | 22 | 1.78#(1.12-2.7) | 1 | 2.82(0.07-15.73) |
| lymphandblood | 177 | 1.40#(1.2-1.62) | 33 | 2.46#(1.69-3.46) | 78 | 1.32#(1.04-1.65) | 64 | 1.21(0.93-1.55) | 2 | 1.25(0.15-4.52) |
| Miscellaneous Malignant Cancer | 343 | 3.56#(3.19-3.95) | 141 | 13.63#(11.48-16.08) | 127 | 2.84#(2.36-3.37) | 72 | 1.79#(1.4-2.26) | 3 | 2.49(0.51-7.28) |
| In situ, benign or unknown behavior neoplasm | 57 | 1.69#(1.28-2.19) | 7 | 2.1(0.84-4.33) | 29 | 1.90#(1.27-2.72) | 21 | 1.44(0.89-2.2) | 0 | 0(0-8.12) |
| Noncancer | 5,092 | 1.29#(1.26-1.33) | 678 | 1.75#(1.62-1.89) | 2,072 | 1.17#(1.12-1.22) | 2,287 | 1.32#(1.26-1.37) | 55 | 1.02(0.77-1.33) |
| infections | 200 | 1.53#(1.32-1.75) | 39 | 2.79#(1.98-3.81) | 81 | 1.33#(1.05-1.65) | 78 | 1.43#(1.13-1.79) | 2 | 1.23(0.15-4.44) |
| Diabetes Mellitus | 305 | 1.83#(1.63-2.05) | 42 | 2.45#(1.76-3.31) | 127 | 1.68#(1.4-1.99) | 133 | 1.87#(1.57-2.22) | 3 | 1.39(0.29-4.05) |
| Alzheimers (ICD-9 and 10 only) | 144 | 0.77#(0.65-0.9) | 7 | 0.48#(0.19-1) | 40 | 0.53#(0.38-0.73) | 95 | 1(0.81-1.22) | 2 | 0.63(0.08-2.28) |
| Diseases of Heart | 1,722 | 1.31#(1.25-1.37) | 226 | 1.69#(1.47-1.92) | 754 | 1.27#(1.18-1.36) | 730 | 1.28#(1.19-1.38) | 12 | 0.68(0.35-1.19) |
| Hypertension without Heart Disease | 138 | 2.22#(1.86-2.62) | 15 | 2.68#(1.5-4.42) | 49 | 1.81#(1.34-2.39) | 72 | 2.51#(1.97-3.17) | 2 | 2.2(0.27-7.95) |
| Cerebrovascular Diseases | 327 | 1.18#(1.06-1.32) | 40 | 1.47#(1.05-2) | 142 | 1.16(0.98-1.37) | 136 | 1.11(0.93-1.31) | 9 | 2.32#(1.06-4.4) |
| Diseases of Arteries | 62 | 1.13(0.87-1.45) | 11 | 1.81(0.91-3.25) | 19 | 0.75(0.45-1.17) | 32 | 1.42(0.97-2.01) | 0 | 0(0-5.37) |
| Pneumonia and Influenza | 486 | 1.10#(1-1.2) | 55 | 1.25(0.94-1.63) | 192 | 0.96(0.83-1.1) | 233 | 1.21#(1.06-1.38) | 6 | 1.01(0.37-2.2) |
| Digestive | 105 | 1.42#(1.16-1.72) | 11 | 1.38(0.69-2.47) | 49 | 1.42#(1.05-1.87) | 44 | 1.44#(1.05-1.94) | 1 | 1.19(0.03-6.62) |
| Nephritis\ Nephrotic Syndrome\Nephrosis | 321 | 3.04#(2.72-3.39) | 41 | 3.89#(2.79-5.27) | 127 | 2.66#(2.22-3.17) | 148 | 3.22#(2.72-3.78) | 5 | 3.47#(1.13-8.09) |
| Pregnancy\Childbirth\Puerperium | 17 | 3.11#(1.81-4.98) | 2 | 3.33(0.4-12.02) | 9 | 3.54#(1.62-6.71) | 6 | 2.66(0.98-5.79) | 0 | 0(0-59.62) |
| Symptoms, Signs and Ill-Defined Conditions | 62 | 1.09(0.84-1.4) | 11 | 1.87(0.93-3.34) | 23 | 0.84(0.53-1.25) | 28 | 1.23(0.82-1.78) | 0 | 0(0-5.49) |
| Accidents\Adverse Effects\Homicide\Legal intervention | 212 | 1.16#(1.01-1.32) | 25 | 1.34(0.86-1.97) | 93 | 1.12(0.91-1.38) | 93 | 1.17(0.94-1.43) | 1 | 0.43(0.01-2.42) |
| Suicide\Self-Inflicted Injury | 54 | 1.14(0.85-1.48) | 7 | 1.3(0.52-2.67) | 27 | 1.19(0.78-1.72) | 20 | 1.07(0.65-1.65) | 0 | 0(0-7.38) |
| Other Cause of Death | 937 | 1.12#(1.05-1.2) | 146 | 1.93#(1.63-2.27) | 340 | 0.92(0.83-1.03) | 439 | 1.16#(1.05-1.27) | 12 | 1.01(0.52-1.77) |

Supplementary Table 19. Standardized-mortality ratios (SMRs) for each cause of death following RCC diagnosis in patients diagnosis in 2012-2015.

|  | Timing of Death After Diagnosis | | | | | | | | | |
| --- | --- | --- | --- | --- | --- | --- | --- | --- | --- | --- |
|  | All Y ears | | <1 y | | 1 to <5 y | | 5 to <10 y | | ≥10 y | |
| All Causes of Death | 10,389 | 3.30#(3.23-3.36) | 4,067 | 7.02#(6.81-7.24) | 5,792 | 2.55#(2.49-2.62) | 530 | 1.74#(1.6-1.9) | 0 | 0(0-0) |
| All Malignant Cancers | 7,207 | 9.27#(9.05-9.48) | 3,346 | 22.42#(21.66-23.19) | 3,642 | 6.54#(6.33-6.76) | 219 | 3.05#(2.66-3.48) | 0 | 0(0-0) |
| Oral Cavity/Pharynx/eye/endocrine | 13 | 0.73(0.39-1.25) | 5 | 1.5(0.49-3.5) | 8 | 0.63(0.27-1.23) | 0 | 0(0-2.28) | 0 | 0(0-0) |
| Digestive System | 225 | 1.07(0.94-1.22) | 56 | 1.41#(1.06-1.83) | 154 | 1.02(0.87-1.2) | 15 | 0.78(0.43-1.28) | 0 | 0(0-0) |
| Respiratory System | 270 | 1.25#(1.1-1.4) | 69 | 1.59#(1.24-2.02) | 177 | 1.15(0.99-1.33) | 24 | 1.23(0.79-1.84) | 0 | 0(0-0) |
| Bones and Joints/soft tissue/skin | 33 | 1.39(0.96-1.95) | 16 | 3.51#(2.01-5.7) | 15 | 0.88(0.49-1.45) | 2 | 0.92(0.11-3.31) | 0 | 0(0-0) |
| Breast | 9 | 0.27#(0.12-0.5) | 3 | 0.46(0.09-1.34) | 5 | 0.21#(0.07-0.48) | 1 | 0.32(0.01-1.77) | 0 | 0(0-0) |
| Genital System | 57 | 0.73#(0.55-0.94) | 17 | 1.18(0.69-1.9) | 38 | 0.68#(0.48-0.93) | 2 | 0.26#(0.03-0.96) | 0 | 0(0-0) |
| Kidney and Renal Pelvis | 6,144 | 312.14#(304.38-320.04) | 2,985 | 787.80#(759.79-816.58) | 3,009 | 213.47#(205.91-221.23) | 150 | 83.39#(70.58-97.85) | 0 | 0(0-0) |
| Other Urinary Organs | 71 | 2.83#(2.21-3.57) | 28 | 6.10#(4.06-8.82) | 40 | 2.21#(1.58-3.01) | 3 | 1.23(0.25-3.59) | 0 | 0(0-0) |
| Brain/Other Nervous System | 28 | 1.45(0.96-2.09) | 5 | 1.36(0.44-3.17) | 22 | 1.58(0.99-2.39) | 1 | 0.57(0.01-3.19) | 0 | 0(0-0) |
| lymphandblood | 83 | 1.1(0.88-1.37) | 26 | 1.82#(1.19-2.66) | 48 | 0.89(0.66-1.18) | 9 | 1.27(0.58-2.41) | 0 | 0(0-0) |
| Miscellaneous Malignant Cancer | 274 | 4.70#(4.16-5.29) | 136 | 12.32#(10.34-14.57) | 126 | 3.02#(2.51-3.59) | 12 | 2.20#(1.14-3.85) | 0 | 0(0-0) |
| In situ, benign or unknown behavior neoplasm | 43 | 2.18#(1.58-2.93) | 19 | 5.25#(3.16-8.19) | 19 | 1.34(0.81-2.09) | 5 | 2.6(0.84-6.06) | 0 | 0(0-0) |
| Noncancer | 3,139 | 1.33#(1.29-1.38) | 702 | 1.65#(1.53-1.77) | 2,131 | 1.26#(1.2-1.31) | 306 | 1.33#(1.18-1.48) | 0 | 0(0-0) |
| infections | 145 | 1.83#(1.55-2.16) | 48 | 3.18#(2.35-4.22) | 85 | 1.50#(1.2-1.85) | 12 | 1.63(0.84-2.85) | 0 | 0(0-0) |
| Diabetes Mellitus | 211 | 2.04#(1.77-2.34) | 44 | 2.30#(1.67-3.09) | 147 | 1.97#(1.67-2.32) | 20 | 2.05#(1.25-3.16) | 0 | 0(0-0) |
| Alzheimers (ICD-9 and 10 only) | 95 | 0.87(0.7-1.06) | 15 | 0.88(0.49-1.45) | 72 | 0.9(0.71-1.14) | 8 | 0.66(0.28-1.29) | 0 | 0(0-0) |
| Diseases of Heart | 983 | 1.27#(1.19-1.36) | 214 | 1.52#(1.32-1.73) | 675 | 1.22#(1.13-1.31) | 94 | 1.25#(1.01-1.53) | 0 | 0(0-0) |
| Hypertension without Heart Disease | 92 | 2.44#(1.97-2.99) | 17 | 2.59#(1.51-4.14) | 63 | 2.30#(1.77-2.95) | 12 | 3.15#(1.63-5.5) | 0 | 0(0-0) |
| Cerebrovascular Diseases | 197 | 1.23#(1.06-1.41) | 42 | 1.47#(1.06-1.98) | 139 | 1.20#(1.01-1.42) | 16 | 0.99(0.57-1.61) | 0 | 0(0-0) |
| Diseases of Arteries | 51 | 1.66#(1.23-2.18) | 14 | 2.40#(1.31-4.03) | 33 | 1.50#(1.03-2.11) | 4 | 1.36(0.37-3.48) | 0 | 0(0-0) |
| Pneumonia and Influenza | 267 | 1.02(0.9-1.15) | 61 | 1.28(0.98-1.64) | 179 | 0.95(0.82-1.1) | 27 | 1.05(0.69-1.53) | 0 | 0(0-0) |
| Digestive | 74 | 1.47#(1.15-1.84) | 18 | 1.86#(1.1-2.94) | 49 | 1.35(1-1.78) | 7 | 1.59(0.64-3.28) | 0 | 0(0-0) |
| Nephritis\ Nephrotic Syndrome\Nephrosis | 170 | 2.74#(2.34-3.18) | 31 | 2.74#(1.86-3.89) | 117 | 2.62#(2.16-3.14) | 22 | 3.62#(2.27-5.48) | 0 | 0(0-0) |
| Pregnancy\Childbirth\Puerperium | 13 | 3.49#(1.86-5.98) | 3 | 4.21(0.87-12.32) | 8 | 2.98#(1.29-5.87) | 2 | 6.18(0.75-22.32) | 0 | 0(0-0) |
| Symptoms, Signs and Ill-Defined Conditions | 51 | 1.67#(1.24-2.19) | 16 | 2.65#(1.51-4.3) | 32 | 1.48#(1.01-2.08) | 3 | 1.05(0.22-3.07) | 0 | 0(0-0) |
| Accidents\Adverse Effects\Homicide\Legal intervention | 143 | 1.17(0.98-1.38) | 29 | 1.28(0.86-1.84) | 105 | 1.18(0.97-1.43) | 9 | 0.8(0.37-1.53) | 0 | 0(0-0) |
| Suicide\Self-Inflicted Injury | 34 | 1.04(0.72-1.45) | 7 | 1.08(0.43-2.22) | 25 | 1.07(0.69-1.57) | 2 | 0.73(0.09-2.65) | 0 | 0(0-0) |
| Other Cause of Death | 613 | 1.23#(1.14-1.33) | 143 | 1.62#(1.37-1.91) | 402 | 1.12#(1.01-1.23) | 68 | 1.36#(1.06-1.73) | 0 | 0(0-0) |

Supplementary Table 20. Standardized-mortality ratios (SMRs) for each cause of death following RCC diagnosis in patients with clear cell histology.

|  | Timing of Death After Diagnosis | | | | | | | | | |
| --- | --- | --- | --- | --- | --- | --- | --- | --- | --- | --- |
|  | All Y ears | | <1 y | | 1 to <5 y | | 5 to <10 y | | ≥10 y | |
| All Causes of Death | 31,809 | 2.79#(2.76-2.82) | 9,306 | 7.60#(7.45-7.76) | 13,591 | 2.61#(2.57-2.66) | 7,083 | 1.82#(1.78-1.86) | 1,829 | 1.68#(1.6-1.76) |
| All Malignant Cancers | 20,423 | 7.37#(7.27-7.48) | 7,719 | 24.14#(23.6-24.68) | 8,858 | 6.79#(6.65-6.93) | 3,120 | 3.44#(3.32-3.56) | 726 | 3.06#(2.84-3.29) |
| Oral Cavity/Pharynx/eye/endocrine | 55 | 0.95(0.72-1.24) | 10 | 1.52(0.73-2.8) | 21 | 0.77(0.48-1.18) | 18 | 0.94(0.56-1.49) | 6 | 1.19(0.44-2.58) |
| Digestive System | 839 | 1.17#(1.09-1.25) | 125 | 1.53#(1.27-1.82) | 341 | 1.01(0.91-1.12) | 281 | 1.19#(1.06-1.34) | 92 | 1.48#(1.2-1.82) |
| Respiratory System | 942 | 1.18#(1.11-1.26) | 147 | 1.52#(1.28-1.78) | 420 | 1.1(1-1.21) | 295 | 1.16#(1.03-1.3) | 80 | 1.27#(1.01-1.58) |
| Bones and Joints/soft tissue/skin | 95 | 1.13(0.92-1.38) | 28 | 2.96#(1.97-4.28) | 35 | 0.89(0.62-1.24) | 24 | 0.86(0.55-1.28) | 8 | 1.09(0.47-2.14) |
| Breast | 58 | 0.45#(0.34-0.58) | 5 | 0.33#(0.11-0.78) | 15 | 0.25#(0.14-0.41) | 25 | 0.59#(0.38-0.88) | 13 | 1.19(0.63-2.04) |
| Genital System | 188 | 0.67#(0.58-0.77) | 32 | 1.04(0.71-1.47) | 79 | 0.61#(0.48-0.76) | 54 | 0.57#(0.43-0.74) | 23 | 0.88(0.56-1.32) |
| Kidney and Renal Pelvis | 17,045 | 248.42#(244.71-252.18) | 7,059 | 892.00#(871.31-913.06) | 7,455 | 230.35#(225.15-235.64) | 2,116 | 94.14#(90.17-98.24) | 415 | 70.83#(64.18-77.99) |
| Other Urinary Organs | 115 | 1.27#(1.05-1.53) | 20 | 2.11#(1.29-3.26) | 59 | 1.44#(1.1-1.86) | 30 | 0.96(0.65-1.37) | 6 | 0.68(0.25-1.48) |
| Brain/Other Nervous System | 110 | 1.71#(1.41-2.07) | 15 | 2.02#(1.13-3.33) | 56 | 1.84#(1.39-2.39) | 31 | 1.49#(1.01-2.11) | 8 | 1.48(0.64-2.92) |
| lymphandblood | 250 | 0.91(0.8-1.03) | 25 | 0.81(0.52-1.2) | 104 | 0.82#(0.67-0.99) | 92 | 1.01(0.81-1.23) | 29 | 1.19(0.8-1.71) |
| Miscellaneous Malignant Cancer | 726 | 3.48#(3.23-3.74) | 253 | 10.62#(9.35-12.01) | 273 | 2.79#(2.47-3.15) | 154 | 2.24#(1.9-2.62) | 46 | 2.50#(1.83-3.34) |
| In situ, benign or unknown behavior neoplasm | 130 | 1.77#(1.48-2.11) | 27 | 3.51#(2.31-5.11) | 59 | 1.78#(1.35-2.29) | 37 | 1.46#(1.03-2.01) | 7 | 0.99(0.4-2.03) |
| Noncancer | 11,256 | 1.31#(1.29-1.34) | 1,560 | 1.74#(1.66-1.83) | 4,674 | 1.21#(1.18-1.25) | 3,926 | 1.33#(1.29-1.37) | 1,096 | 1.30#(1.22-1.37) |
| infections | 461 | 1.66#(1.51-1.82) | 91 | 2.91#(2.34-3.57) | 186 | 1.44#(1.24-1.66) | 143 | 1.55#(1.31-1.83) | 41 | 1.66#(1.19-2.26) |
| Diabetes Mellitus | 731 | 2.06#(1.91-2.22) | 110 | 2.79#(2.29-3.36) | 311 | 1.89#(1.68-2.11) | 246 | 2.08#(1.83-2.36) | 64 | 1.99#(1.53-2.55) |
| Alzheimers (ICD-9 and 10 only) | 360 | 0.87#(0.78-0.96) | 23 | 0.67(0.42-1) | 124 | 0.73#(0.61-0.87) | 157 | 1(0.85-1.17) | 56 | 1.05(0.79-1.36) |
| Diseases of Heart | 3,754 | 1.31#(1.27-1.35) | 531 | 1.71#(1.57-1.86) | 1,570 | 1.20#(1.14-1.26) | 1,283 | 1.31#(1.24-1.39) | 370 | 1.34#(1.2-1.48) |
| Hypertension without Heart Disease | 278 | 2.11#(1.87-2.37) | 32 | 2.51#(1.72-3.55) | 98 | 1.69#(1.37-2.06) | 119 | 2.52#(2.09-3.02) | 29 | 2.05#(1.37-2.95) |
| Cerebrovascular Diseases | 760 | 1.25#(1.16-1.34) | 100 | 1.57#(1.28-1.91) | 347 | 1.27#(1.14-1.42) | 240 | 1.14#(1-1.29) | 73 | 1.18(0.93-1.49) |
| Diseases of Arteries | 150 | 1.23#(1.04-1.44) | 26 | 1.83#(1.2-2.69) | 62 | 1.09(0.83-1.4) | 52 | 1.29(0.97-1.7) | 10 | 0.92(0.44-1.7) |
| Pneumonia and Influenza | 1,066 | 1.10#(1.03-1.16) | 124 | 1.21#(1-1.44) | 436 | 0.99(0.9-1.08) | 414 | 1.24#(1.12-1.36) | 92 | 0.98(0.79-1.2) |
| Digestive | 239 | 1.52#(1.33-1.72) | 29 | 1.55#(1.04-2.23) | 114 | 1.50#(1.24-1.81) | 82 | 1.63#(1.3-2.03) | 14 | 1.11(0.6-1.86) |
| Nephritis\ Nephrotic Syndrome\Nephrosis | 614 | 2.71#(2.5-2.94) | 81 | 3.39#(2.69-4.22) | 250 | 2.44#(2.14-2.76) | 215 | 2.76#(2.4-3.16) | 68 | 3.11#(2.41-3.94) |
| Pregnancy\Childbirth\Puerperium | 33 | 2.80#(1.93-3.94) | 4 | 2.82(0.77-7.22) | 13 | 2.29#(1.22-3.91) | 12 | 3.22#(1.66-5.62) | 4 | 4.25#(1.16-10.88) |
| Symptoms, Signs and Ill-Defined Conditions | 176 | 1.43#(1.23-1.66) | 37 | 2.85#(2-3.92) | 80 | 1.43#(1.13-1.78) | 54 | 1.26(0.95-1.65) | 5 | 0.45(0.15-1.06) |
| Accidents\Adverse Effects\Homicide\Legal intervention | 455 | 1.16#(1.06-1.27) | 59 | 1.35#(1.03-1.74) | 219 | 1.20#(1.04-1.37) | 139 | 1.07(0.9-1.26) | 38 | 1.07(0.76-1.47) |
| Suicide\Self-Inflicted Injury | 108 | 1.06(0.87-1.28) | 15 | 1.18(0.66-1.95) | 54 | 1.08(0.81-1.41) | 34 | 1.08(0.75-1.51) | 5 | 0.66(0.22-1.55) |
| Other Cause of Death | 2,071 | 1.15#(1.1-1.2) | 298 | 1.71#(1.52-1.92) | 810 | 1.03(0.96-1.1) | 736 | 1.14#(1.06-1.23) | 227 | 1.20#(1.05-1.36) |

Supplementary Table 21. Standardized-mortality ratios (SMRs) for each cause of death following RCC diagnosis in patients with Papillary histology.

|  | Timing of Death After Diagnosis | | | | | | | | | |
| --- | --- | --- | --- | --- | --- | --- | --- | --- | --- | --- |
|  | All Y ears | | <1 y | | 1 to <5 y | | 5 to <10 y | | ≥10 y | |
| All Causes of Death | 3,274 | 1.79#(1.73-1.86) | 592 | 3.31#(3.05-3.58) | 1,524 | 1.83#(1.74-1.93) | 910 | 1.42#(1.33-1.52) | 248 | 1.41#(1.24-1.6) |
| All Malignant Cancers | 1,605 | 3.49#(3.32-3.66) | 412 | 8.44#(7.64-9.29) | 809 | 3.73#(3.47-3.99) | 300 | 1.94#(1.72-2.17) | 84 | 2.14#(1.7-2.65) |
| Oral Cavity/Pharynx/eye/endocrine | 7 | 0.69(0.28-1.42) | 2 | 1.84(0.22-6.63) | 4 | 0.83(0.23-2.12) | 0 | 0(0-1.08) | 1 | 1.17(0.03-6.5) |
| Digestive System | 103 | 0.83(0.68-1.01) | 7 | 0.54(0.22-1.11) | 46 | 0.79(0.58-1.05) | 34 | 0.82(0.57-1.14) | 16 | 1.53(0.87-2.48) |
| Respiratory System | 126 | 0.93(0.78-1.11) | 10 | 0.66(0.32-1.21) | 58 | 0.89(0.68-1.15) | 43 | 0.98(0.71-1.31) | 15 | 1.42(0.8-2.35) |
| Bones and Joints/soft tissue/skin | 10 | 0.76(0.36-1.39) | 1 | 0.74(0.02-4.12) | 5 | 0.82(0.26-1.9) | 3 | 0.66(0.14-1.94) | 1 | 0.84(0.02-4.67) |
| Breast | 5 | 0.38#(0.12-0.89) | 0 | 0(0-2.48) | 1 | 0.16#(0-0.87) | 3 | 0.7(0.14-2.05) | 1 | 1(0.03-5.57) |
| Genital System | 29 | 0.60#(0.4-0.85) | 2 | 0.42(0.05-1.53) | 12 | 0.54#(0.28-0.95) | 10 | 0.58(0.28-1.07) | 5 | 1.06(0.34-2.47) |
| Kidney and Renal Pelvis | 1,192 | 102.23#(96.51-108.2) | 378 | 306.49#(276.37-339) | 621 | 112.98#(104.27-122.23) | 154 | 39.19#(33.25-45.89) | 39 | 38.98#(27.72-53.28) |
| Other Urinary Organs | 19 | 1.25(0.75-1.95) | 1 | 0.71(0.02-3.94) | 10 | 1.47(0.71-2.71) | 8 | 1.46(0.63-2.88) | 0 | 0(0-2.36) |
| Brain/Other Nervous System | 24 | 2.44#(1.57-3.63) | 0 | 0(0-3.48) | 9 | 1.92(0.88-3.64) | 14 | 4.29#(2.35-7.2) | 1 | 1.23(0.03-6.86) |
| lymphandblood | 37 | 0.82(0.58-1.13) | 2 | 0.44(0.05-1.57) | 19 | 0.91(0.55-1.42) | 15 | 0.97(0.54-1.59) | 1 | 0.24(0.01-1.36) |
| Miscellaneous Malignant Cancer | 53 | 1.53#(1.14-2) | 9 | 2.47#(1.13-4.69) | 24 | 1.48(0.95-2.2) | 16 | 1.36(0.78-2.21) | 4 | 1.31(0.36-3.36) |
| In situ, benign or unknown behavior neoplasm | 19 | 1.66#(1-2.59) | 2 | 1.87(0.23-6.76) | 9 | 1.76(0.8-3.34) | 7 | 1.71(0.69-3.52) | 1 | 0.87(0.02-4.83) |
| Noncancer | 1,650 | 1.22#(1.16-1.28) | 178 | 1.38#(1.18-1.6) | 706 | 1.16#(1.07-1.25) | 603 | 1.26#(1.16-1.36) | 163 | 1.21#(1.03-1.41) |
| infections | 90 | 1.83#(1.47-2.25) | 15 | 2.84#(1.59-4.68) | 37 | 1.59#(1.12-2.2) | 32 | 1.94#(1.33-2.74) | 6 | 1.42(0.52-3.1) |
| Diabetes Mellitus | 88 | 1.41#(1.13-1.73) | 6 | 0.94(0.35-2.05) | 43 | 1.48#(1.07-1.99) | 32 | 1.49#(1.02-2.11) | 7 | 1.25(0.5-2.57) |
| Alzheimers (ICD-9 and 10 only) | 43 | 0.78(0.57-1.06) | 1 | 0.26(0.01-1.45) | 11 | 0.50#(0.25-0.9) | 26 | 1.2(0.78-1.75) | 5 | 0.67(0.22-1.57) |
| Diseases of Heart | 515 | 1.10#(1.01-1.2) | 63 | 1.38#(1.06-1.76) | 224 | 1.06(0.92-1.2) | 183 | 1.12(0.96-1.29) | 45 | 0.99(0.72-1.32) |
| Hypertension without Heart Disease | 60 | 2.70#(2.06-3.47) | 7 | 3.51#(1.41-7.24) | 32 | 3.25#(2.22-4.59) | 13 | 1.61(0.86-2.75) | 8 | 3.46#(1.5-6.82) |
| Cerebrovascular Diseases | 95 | 1(0.81-1.23) | 10 | 1.12(0.54-2.06) | 42 | 0.99(0.71-1.34) | 34 | 1.01(0.7-1.41) | 9 | 0.93(0.43-1.77) |
| Diseases of Arteries | 25 | 1.3(0.84-1.92) | 3 | 1.51(0.31-4.4) | 10 | 1.12(0.54-2.06) | 8 | 1.22(0.53-2.4) | 4 | 2.29(0.62-5.87) |
| Pneumonia and Influenza | 134 | 0.91(0.76-1.08) | 12 | 0.86(0.45-1.51) | 54 | 0.81(0.61-1.06) | 57 | 1.09(0.82-1.41) | 11 | 0.76(0.38-1.35) |
| Digestive | 29 | 1.14(0.77-1.64) | 3 | 1.04(0.21-3.04) | 14 | 1.13(0.62-1.9) | 9 | 1.1(0.5-2.09) | 3 | 1.55(0.32-4.52) |
| Nephritis\ Nephrotic Syndrome\Nephrosis | 151 | 3.82#(3.23-4.48) | 12 | 3.18#(1.64-5.56) | 58 | 3.24#(2.46-4.19) | 60 | 4.28#(3.27-5.51) | 21 | 5.45#(3.37-8.33) |
| Pregnancy\Childbirth\Puerperium | 6 | 3.42#(1.26-7.45) | 1 | 4.97(0.13-27.7) | 4 | 4.68#(1.27-11.97) | 1 | 1.78(0.05-9.93) | 0 | 0(0-27.39) |
| Symptoms, Signs and Ill-Defined Conditions | 20 | 1.1(0.67-1.69) | 3 | 1.7(0.35-4.98) | 7 | 0.84(0.34-1.73) | 5 | 0.77(0.25-1.79) | 5 | 2.99(0.97-6.97) |
| Accidents\Adverse Effects\Homicide\Legal intervention | 75 | 1.17(0.92-1.47) | 8 | 1.19(0.51-2.35) | 36 | 1.2(0.84-1.66) | 24 | 1.12(0.72-1.66) | 7 | 1.23(0.5-2.54) |
| Suicide\Self-Inflicted Injury | 15 | 0.98(0.55-1.62) | 1 | 0.56(0.01-3.12) | 7 | 0.94(0.38-1.93) | 4 | 0.82(0.22-2.11) | 3 | 2.6(0.54-7.61) |
| Other Cause of Death | 304 | 1.11(0.99-1.25) | 33 | 1.38(0.95-1.94) | 127 | 1.06(0.89-1.27) | 115 | 1.14(0.95-1.37) | 29 | 0.99(0.66-1.42) |

Supplementary Table 22. Standardized-mortality ratios (SMRs) for each cause of death following RCC diagnosis in patients with chromophobe histology.

|  | Timing of Death After Diagnosis | | | | | | | | | |
| --- | --- | --- | --- | --- | --- | --- | --- | --- | --- | --- |
|  | All Y ears | | <1 y | | 1 to <5 y | | 5 to <10 y | | ≥10 y | |
| All Causes of Death | 914 | 1.08#(1.01-1.16) | 109 | 1.48#(1.21-1.78) | 374 | 1.02(0.92-1.13) | 320 | 1.03(0.92-1.15) | 111 | 1.19(0.98-1.44) |
| All Malignant Cancers | 395 | 1.95#(1.77-2.16) | 75 | 3.84#(3.02-4.81) | 175 | 1.91#(1.64-2.21) | 108 | 1.52#(1.24-1.83) | 37 | 1.88#(1.32-2.59) |
| Oral Cavity/Pharynx/eye/endocrine | 4 | 0.96(0.26-2.45) | 1 | 2.52(0.06-14.02) | 1 | 0.53(0.01-2.95) | 0 | 0(0-2.5) | 2 | 4.81(0.58-17.39) |
| Digestive System | 45 | 0.86(0.63-1.15) | 1 | 0.2(0.01-1.12) | 22 | 0.93(0.58-1.41) | 17 | 0.92(0.54-1.47) | 5 | 0.98(0.32-2.28) |
| Respiratory System | 34 | 0.60#(0.41-0.83) | 3 | 0.51(0.11-1.5) | 11 | 0.41#(0.21-0.74) | 13 | 0.67(0.35-1.14) | 7 | 1.37(0.55-2.83) |
| Bones and Joints/soft tissue/skin | 5 | 0.83(0.27-1.93) | 0 | 0(0-6.52) | 3 | 1.11(0.23-3.24) | 2 | 0.92(0.11-3.34) | 0 | 0(0-5.99) |
| Breast | 3 | 0.29#(0.06-0.84) | 0 | 0(0-3.48) | 0 | 0.00#(0-0.76) | 3 | 0.83(0.17-2.44) | 0 | 0(0-4.05) |
| Genital System | 16 | 0.75(0.43-1.22) | 1 | 0.52(0.01-2.91) | 7 | 0.75(0.3-1.55) | 8 | 1.04(0.45-2.05) | 0 | 0(0-1.61) |
| Kidney and Renal Pelvis | 240 | 48.85#(42.87-55.44) | 62 | 131.34#(100.7-168.37) | 112 | 50.34#(41.45-60.58) | 53 | 30.58#(22.91-40) | 13 | 26.92#(14.34-46.04) |
| Other Urinary Organs | 2 | 0.31(0.04-1.11) | 1 | 1.82(0.05-10.15) | 0 | 0(0-1.32) | 0 | 0(0-1.51) | 1 | 1.32(0.03-7.36) |
| Brain/Other Nervous System | 6 | 1.3(0.48-2.82) | 2 | 4.38(0.53-15.83) | 2 | 0.94(0.11-3.39) | 1 | 0.62(0.02-3.47) | 1 | 2.31(0.06-12.88) |
| lymphandblood | 20 | 1.01(0.61-1.55) | 0 | 0(0-2) | 9 | 1.02(0.46-1.93) | 5 | 0.7(0.23-1.64) | 6 | 2.95#(1.08-6.42) |
| Miscellaneous Malignant Cancer | 20 | 1.32(0.8-2.03) | 4 | 2.78(0.76-7.11) | 8 | 1.17(0.51-2.31) | 6 | 1.11(0.41-2.41) | 2 | 1.3(0.16-4.71) |
| In situ, benign or unknown behavior neoplasm | 6 | 1.12(0.41-2.45) | 0 | 0(0-8.16) | 1 | 0.44(0.01-2.43) | 4 | 2.01(0.55-5.14) | 1 | 1.66(0.04-9.26) |
| Noncancer | 513 | 0.81#(0.74-0.88) | 34 | 0.63#(0.44-0.88) | 198 | 0.73#(0.63-0.84) | 208 | 0.87(0.76-1) | 73 | 1(0.79-1.26) |
| infections | 18 | 0.86(0.51-1.35) | 0 | 0(0-1.86) | 10 | 1.06(0.51-1.96) | 5 | 0.67(0.22-1.56) | 3 | 1.41(0.29-4.12) |
| Diabetes Mellitus | 31 | 1.17(0.8-1.66) | 3 | 1.22(0.25-3.57) | 11 | 0.93(0.46-1.66) | 11 | 1.16(0.58-2.08) | 6 | 2.21(0.81-4.8) |
| Alzheimers (ICD-9 and 10 only) | 19 | 0.61#(0.36-0.94) | 0 | 0(0-1.88) | 6 | 0.5(0.18-1.1) | 8 | 0.62(0.27-1.23) | 5 | 1.07(0.35-2.51) |
| Diseases of Heart | 171 | 0.81#(0.69-0.94) | 11 | 0.6(0.3-1.07) | 71 | 0.78#(0.61-0.98) | 73 | 0.93(0.73-1.17) | 16 | 0.67(0.38-1.08) |
| Hypertension without Heart Disease | 19 | 1.88#(1.13-2.94) | 0 | 0(0-4.66) | 3 | 0.71(0.15-2.08) | 9 | 2.32#(1.06-4.41) | 7 | 5.71#(2.3-11.77) |
| Cerebrovascular Diseases | 29 | 0.64#(0.43-0.92) | 4 | 1.06(0.29-2.71) | 10 | 0.52#(0.25-0.96) | 10 | 0.59(0.28-1.08) | 5 | 0.94(0.31-2.2) |
| Diseases of Arteries | 7 | 0.79(0.32-1.62) | 0 | 0(0-4.46) | 3 | 0.76(0.16-2.22) | 3 | 0.94(0.19-2.74) | 1 | 1.08(0.03-6.03) |
| Pneumonia and Influenza | 35 | 0.50#(0.35-0.69) | 1 | 0.17#(0-0.93) | 14 | 0.46#(0.25-0.77) | 13 | 0.50#(0.27-0.85) | 7 | 0.9(0.36-1.85) |
| Digestive | 7 | 0.61(0.24-1.25) | 2 | 1.72(0.21-6.22) | 3 | 0.56(0.12-1.64) | 0 | 0.00#(0-0.93) | 2 | 1.91(0.23-6.88) |
| Nephritis\ Nephrotic Syndrome\Nephrosis | 39 | 2.27#(1.61-3.1) | 4 | 2.71(0.74-6.94) | 15 | 2.02#(1.13-3.33) | 15 | 2.35#(1.32-3.88) | 5 | 2.6(0.84-6.06) |
| Pregnancy\Childbirth\Puerperium | 0 | 0(0-4) | 0 | 0(0-38.47) | 0 | 0(0-8.55) | 0 | 0(0-11.82) | 0 | 0(0-44.88) |
| Symptoms, Signs and Ill-Defined Conditions | 11 | 1.19(0.6-2.13) | 0 | 0(0-4.72) | 3 | 0.76(0.16-2.22) | 8 | 2.27(0.98-4.48) | 0 | 0(0-3.82) |
| Accidents\Adverse Effects\Homicide\Legal intervention | 28 | 0.93(0.62-1.35) | 3 | 1.07(0.22-3.12) | 8 | 0.6(0.26-1.18) | 12 | 1.13(0.58-1.97) | 5 | 1.61(0.52-3.76) |
| Suicide\Self-Inflicted Injury | 8 | 1.05(0.45-2.07) | 1 | 1.22(0.03-6.82) | 6 | 1.65(0.61-3.6) | 1 | 0.4(0.01-2.21) | 0 | 0(0-5.74) |
| Other Cause of Death | 91 | 0.68#(0.54-0.83) | 5 | 0.48(0.16-1.12) | 35 | 0.63#(0.44-0.87) | 40 | 0.77(0.55-1.05) | 11 | 0.67(0.34-1.21) |

Supplementary Table 23. Standardized-mortality ratios (SMRs) for each cause of death following RCC diagnosis in patients with other histology type.

|  | Timing of Death After Diagnosis | | | | | | | | | |
| --- | --- | --- | --- | --- | --- | --- | --- | --- | --- | --- |
|  | All Y ears | | <1 y | | 1 to <5 y | | 5 to <10 y | | ≥10 y | |
| All Causes of Death | 3,633 | 4.37#(4.23-4.51) | 1,735 | 15.66#(14.93-16.42) | 1,342 | 3.53#(3.35-3.73) | 457 | 1.75#(1.59-1.92) | 99 | 1.25#(1.02-1.52) |
| All Malignant Cancers | 2,673 | 14.16#(13.63-14.71) | 1,514 | 57.43#(54.57-60.39) | 945 | 10.68#(10.01-11.38) | 188 | 3.25#(2.8-3.75) | 26 | 1.62#(1.06-2.38) |
| Oral Cavity/Pharynx/eye/endocrine | 5 | 1.33(0.43-3.11) | 3 | 5.91#(1.22-17.26) | 1 | 0.57(0.01-3.19) | 1 | 0.86(0.02-4.77) | 0 | 0(0-11.22) |
| Digestive System | 75 | 1.55#(1.22-1.94) | 24 | 3.59#(2.3-5.35) | 25 | 1.1(0.71-1.63) | 24 | 1.61#(1.03-2.4) | 2 | 0.49(0.06-1.76) |
| Respiratory System | 103 | 1.94#(1.58-2.35) | 44 | 5.67#(4.12-7.62) | 31 | 1.22(0.83-1.74) | 26 | 1.64#(1.07-2.4) | 2 | 0.49(0.06-1.75) |
| Bones and Joints/soft tissue/skin | 16 | 2.87#(1.64-4.66) | 8 | 10.73#(4.63-21.13) | 3 | 1.17(0.24-3.43) | 3 | 1.71(0.35-4.99) | 2 | 3.91(0.47-14.11) |
| Breast | 5 | 0.62(0.2-1.45) | 1 | 0.86(0.02-4.79) | 2 | 0.52(0.06-1.89) | 1 | 0.41(0.01-2.31) | 1 | 1.6(0.04-8.89) |
| Genital System | 18 | 0.85(0.51-1.35) | 7 | 2.45(0.98-5.04) | 8 | 0.82(0.35-1.61) | 2 | 0.31(0.04-1.1) | 1 | 0.52(0.01-2.89) |
| Kidney and Renal Pelvis | 2,018 | 441.62#(422.56-461.32) | 1,199 | 1,902.79#(1796.6-2013.62) | 710 | 332.98#(308.94-358.4) | 98 | 69.38#(56.33-84.55) | 11 | 27.87#(13.91-49.88) |
| Other Urinary Organs | 94 | 14.04#(11.35-17.19) | 46 | 53.59#(39.24-71.48) | 43 | 14.30#(10.35-19.26) | 5 | 2.32(0.75-5.42) | 0 | 0(0-5.47) |
| Brain/Other Nervous System | 1 | 0.26(0.01-1.45) | 1 | 1.89(0.05-10.54) | 0 | 0(0-2.05) | 0 | 0(0-3.1) | 0 | 0(0-11.31) |
| lymphandblood | 133 | 6.92#(5.79-8.2) | 60 | 22.83#(17.42-29.39) | 53 | 5.96#(4.46-7.8) | 18 | 3.01#(1.78-4.75) | 2 | 1.17(0.14-4.21) |
| Miscellaneous Malignant Cancer | 205 | 14.16#(12.29-16.24) | 121 | 60.26#(50-72) | 69 | 10.23#(7.96-12.95) | 10 | 2.24#(1.08-4.13) | 5 | 3.94#(1.28-9.2) |
| In situ, benign or unknown behavior neoplasm | 20 | 3.71#(2.26-5.73) | 9 | 12.78#(5.84-24.25) | 5 | 2.05(0.67-4.78) | 4 | 2.32(0.63-5.94) | 2 | 3.8(0.46-13.74) |
| Noncancer | 940 | 1.48#(1.38-1.57) | 212 | 2.53#(2.2-2.9) | 392 | 1.36#(1.23-1.5) | 265 | 1.31#(1.16-1.48) | 71 | 1.13(0.88-1.43) |
| infections | 37 | 1.85#(1.3-2.54) | 11 | 3.98#(1.99-7.12) | 17 | 1.81#(1.06-2.9) | 9 | 1.46(0.67-2.77) | 0 | 0(0-2.12) |
| Diabetes Mellitus | 58 | 2.29#(1.74-2.96) | 7 | 2.03(0.82-4.18) | 27 | 2.28#(1.51-3.32) | 20 | 2.55#(1.56-3.94) | 4 | 1.79(0.49-4.58) |
| Alzheimers (ICD-9 and 10 only) | 23 | 0.69(0.44-1.04) | 4 | 1.06(0.29-2.7) | 11 | 0.78(0.39-1.39) | 6 | 0.53(0.2-1.16) | 2 | 0.47(0.06-1.71) |
| Diseases of Heart | 314 | 1.44#(1.28-1.61) | 72 | 2.43#(1.9-3.06) | 125 | 1.25#(1.04-1.49) | 95 | 1.40#(1.13-1.71) | 22 | 1.05(0.66-1.6) |
| Hypertension without Heart Disease | 26 | 2.53#(1.65-3.71) | 3 | 2.36(0.49-6.89) | 14 | 3.04#(1.66-5.1) | 6 | 1.8(0.66-3.92) | 3 | 2.82(0.58-8.23) |
| Cerebrovascular Diseases | 59 | 1.26(0.96-1.62) | 12 | 1.91(0.98-3.33) | 23 | 1.08(0.68-1.62) | 17 | 1.16(0.68-1.86) | 7 | 1.51(0.61-3.12) |
| Diseases of Arteries | 12 | 1.29(0.66-2.25) | 4 | 2.92(0.79-7.47) | 3 | 0.68(0.14-1.99) | 5 | 1.8(0.59-4.21) | 0 | 0(0-4.62) |
| Pneumonia and Influenza | 101 | 1.43#(1.17-1.74) | 27 | 2.88#(1.9-4.19) | 46 | 1.43#(1.05-1.91) | 23 | 1.03(0.65-1.55) | 5 | 0.74(0.24-1.73) |
| Digestive | 19 | 2.08#(1.25-3.25) | 6 | 4.70#(1.73-10.24) | 11 | 2.54#(1.27-4.55) | 2 | 0.72(0.09-2.59) | 0 | 0(0-5.04) |
| Nephritis\ Nephrotic Syndrome\Nephrosis | 55 | 3.10#(2.34-4.04) | 9 | 3.83#(1.75-7.26) | 25 | 3.06#(1.98-4.52) | 16 | 2.88#(1.64-4.67) | 5 | 3.01(0.98-7.03) |
| Pregnancy\Childbirth\Puerperium | 3 | 4.21(0.87-12.3) | 1 | 9.62(0.24-53.57) | 2 | 5.87(0.71-21.21) | 0 | 0(0-17.43) | 0 | 0(0-65.48) |
| Symptoms, Signs and Ill-Defined Conditions | 11 | 1.15(0.57-2.06) | 2 | 1.55(0.19-5.6) | 7 | 1.59(0.64-3.27) | 2 | 0.66(0.08-2.37) | 0 | 0(0-4.42) |
| Accidents\Adverse Effects\Homicide\Legal intervention | 24 | 0.92(0.59-1.37) | 4 | 1.16(0.32-2.98) | 8 | 0.67(0.29-1.32) | 8 | 0.98(0.42-1.93) | 4 | 1.63(0.44-4.18) |
| Suicide\Self-Inflicted Injury | 9 | 1.61(0.74-3.06) | 4 | 5.00#(1.36-12.81) | 2 | 0.75(0.09-2.71) | 2 | 1.19(0.14-4.3) | 1 | 2.27(0.06-12.62) |
| Other Cause of Death | 189 | 1.41#(1.21-1.62) | 46 | 2.80#(2.05-3.73) | 71 | 1.2(0.93-1.51) | 54 | 1.22(0.92-1.59) | 18 | 1.27(0.75-2.01) |
